# Supplementary material for: Detecting Risk Of Postural hypotension (DROP): derivation and validation of a prediction score for primary care
Source: BMJ Open. 2018 Apr 20;8(4):e020740. doi: 10.1136/bmjopen-2017-020740 (PMC5914723; doi:10.1136/bmjopen-2017-020740)
Supplement: Supplementary file 1 [file bmjopen-2017-020740supp001.pdf]

Appendix: Literature search for factors associated with postural hypotension

Demographics:	Increasing age[1-9]
	Female gender[10]
	Nursing home residence[11-15]
Medical History:	Hypertension[7-10 16-20] and uncontrolled hypertension[6 21 22]
	Diabetes and diabetic complications[17 23-28]
	Chronic Kidney Disease[10 29 30]
	Stroke[31-36]
	Ischaemic heart disease[36 37]
	Heart failure[38 39]
	Parkinson's disease[40-42]
	Cognitive impairment[43-50]
	Depression[51]
Medications:	Antiarrhythmic drugs[11]
	Antihypertensives[4 9-11 52-55] (negative association with ACE inhibitors)[10]
	Psychotropic agents (antipsychotics, sedatives, antidepressants)[23 53 56]
	Anticholinesterase inhibitors[50]
Biochemical:	Vitamin D deficiency (conflicting evidence)[1 23 57 58]
Frailty:[59 60]	Falls[61]
	Get up and go test[11]
	Reduced calf mass index[54 62]
	Activity of Daily Living disability score[1 11]
	Cumulative illness Rating Scale for Geriatrics score[23]
Environmental:	Seasons – prevalence higher in summer and in heatwaves[63 64]
	Time of day – higher in mornings[65-68]

References

1. Soysal P, Yay A, Isik AT. Does vitamin D deficiency increase orthostatic hypotension risk in the elderly patients? *Archives of Gerontology and Geriatrics* 2014;59(1):July. doi: http://dx.doi.org/10.1016/j.archger.2014.03.008
2. Moret F, Jaccard-Ruedin H, Bula C, et al. The high diagnostic yield of an outpatient geriatric clinic. *Journal of the American Geriatrics Society Conference: 2012 Annual Scientific Meeting of the American Geriatrics Society Seattle, WA United States Conference Start: 20120503 Conference End: 20120505 Conference Publication: (var pagings)* 2012;60(pp S175):April. doi: http://dx.doi.org/10.1111/j.1532-5415.2012.04000.x
3. Robertson D, Desjardin JA, Lichtenstein MJ. Distribution and observed associations of orthostatic blood pressure changes in elderly general medicine outpatients. *American Journal of the Medical Sciences* 1998;315(5):1998. doi: http://dx.doi.org/10.1097/00000441-199805000-00001
4. Poon IO, Braun U. High prevalence of orthostatic hypotension and its correlation with potentially causative medications among elderly veterans. *Journal of Clinical Pharmacy and Therapeutics* 2005;30(2):April. doi: http://dx.doi.org/10.1111/j.1365-2710.2005.00629.x
5. Mendez CA, Melgarejo JD, Lee JH, et al. Orthostatic hypotension in latino elderly: Findings from the maracaibo ageing study. *Journal of Hypertension Conference: 25th European Meeting on Hypertension and Cardiovascular Protection, ESH 2015 Milan Italy Conference Start: 20150612 Conference End: 20150615 Conference Publication: (var pagings)* 2015;33(pp e219):June. doi: http://dx.doi.org/10.1097/01.hjh.0000468017.03671.63
6. Barochiner J, Alfie J, Aparicio L, et al. Orthostatic hypotension in treated hypertensive patients. *Romanian journal of internal medicine = Revue roumaine de medecine interne* 2012;50(3):2012-2Sep.
7. Shin C, Abbott RD, Lee H, et al. Prevalence and correlates of orthostatic hypotension in middle-aged men and women in Korea: The Korean Health and Genome Study. *Journal of Human Hypertension* 2004;18(10):October. doi: http://dx.doi.org/10.1038/sj.jhh.1001732
8. Harris T, Lipsitz LA, Kleinman JC, et al. Postural change in blood pressure associated with age and systolic blood pressure. The National Health and Nutrition Examination Survey II. *Journals of Gerontology* 1991;46(5):1991.
9. Kamaruzzaman S, Watt H, Carson C, et al. The association between orthostatic hypotension and medication use in the British Women's Heart and Health Study. *Age and Ageing* 2010;39(1):afp192. doi: http://dx.doi.org/10.1093/ageing/afp192
10. Fedorowski A, Burri P, Melander O. Orthostatic hypotension in genetically related hypertensive and normotensive individuals. *Journal of Hypertension* 2009;27(5):May. doi: http://dx.doi.org/10.1097/HJH.0b013e3283279860
11. Boland E, Brackelaire G, Anani Y, et al. Postprandial hypotension among patients from an acute geriatric ward: Results from a pilot study. *European Geriatric Medicine Conference: 11th International Congress of the European Union Geriatric Medicine Society, EUGMS 2015 Oslo Norway Conference Start: 20150916 Conference End: 20150918 Conference Publication: (var pagings)* 2015;6(pp S64):September.
12. Hartog LC, Cizmar-Sweelssen M, Knipscheer A, et al. The association between orthostatic hypotension, falling and successful rehabilitation in a nursing home population. *Archives of Gerontology and Geriatrics* 2015;61(2):01. doi: http://dx.doi.org/10.1016/j.archger.2015.05.005
13. Asensio LE, Aguilera AAC, Corral MACC, et al. Prevalence of orthostatic hypotension in a series of elderly institutionalized patients. *Europace Conference: EHRA Europace 2011 Madrid Spain Conference Start: 20110626 Conference End: 20110629 Conference Publication: (var pagings)* 2011;13 doi: http://dx.doi.org/10.1093/europace/eur231

14. Matusik P, Nowak J, Tomaszewski K, et al. Hypertension among the elderly on the basis of nursing home residents population. [Polish]. *Polski Przeglad Kardiologiczny* 2010;12(3):2010.
15. Wu J-S, Yang Y-C, Lu F-H, et al. Population-based study on the prevalence and correlates of orthostatic hypotension/hypertension and orthostatic dizziness. *Hypertension Research* 2008;31(5):May. doi: http://dx.doi.org/10.1291/hypres.31.897
16. Saez T, Suarez C, Sierra MJ, et al. Orthostatic hypotension in the elderly and its association with the antihypertensive treatment. [Spanish]. *Medicina Clinica* 2000;114(14):15.
17. Wu JS, Yang YC, Lu FH, et al. Population-based study on the prevalence and risk factors of orthostatic hypotension in subjects with pre-diabetes and diabetes. *Diabetes Care* 2009;32(1):69-74. doi: http://dx.doi.org/10.2337/dc08-1389
18. Fan X-H, Sun K, Zhou X-L, et al. Association of orthostatic hypertension and hypotension with target organ damage in middle and old-aged hypertensive patients. [Chinese]. *National Medical Journal of China* 2011;91(4):04. doi: http://dx.doi.org/10.3760/cma.j.issn.0376-2491.2011.04.002
19. Barochiner J, Alfie J, Aparicio LS, et al. Prevalence and clinical profile of resistant hypertension among treated hypertensive subjects. *Clinical and Experimental Hypertension* 2013;35(6):2013. doi: http://dx.doi.org/10.3109/10641963.2012.739236
20. Bouhanick B, Meliani S, Doucet J, et al. Orthostatic hypotension is associated with more severe hypertension in elderly autonomous diabetic patients from the French Gerodiab study at inclusion. *Annales de Cardiologie et d Angeiologie* 2014;63(3):176-82. doi: http://dx.doi.org/10.1016/j.ancard.2014.05.013
21. Auseon A, Ooi WL, Hossain M, et al. Blood pressure behavior in the nursing home: Implications for diagnosis and treatment of hypertension. *Journal of the American Geriatrics Society* 1999;47(3):March.
22. Masuo K, Mikami H, Ogihara T, et al. Changes in frequency of orthostatic hypotension in elderly hypertensive patients under medications. *American Journal of Hypertension* 1996;9(3):March. doi: http://dx.doi.org/10.1016/0895-7061%2895%2900348-7
23. Paccalin M. Factors associated with orthostatic hypotension in hospitalized elderly patients. *European Geriatric Medicine Conference: 11th International Congress of the European Union Geriatric Medicine Society, EUGMS 2015 Oslo Norway Conference Start: 20150916 Conference End: 20150918 Conference Publication: (var pagings)* 2015;6(pp S62):September.
24. van Hateren KJJ, Kleefstra N, Blanker MH, et al. Orthostatic hypotension, diabetes, and falling in older patients: A cross-sectional study. *British Journal of General Practice* 2012;62(603):October. doi: http://dx.doi.org/10.3399/bjgp12X656838
25. Gupta A, Gilden J. Prevalence of diabetes in patients admitted to the hospital with primary diagnosis of orthostatic hypotension. *Diabetes Conference: 73rd Scientific Sessions of the American Diabetes Association Chicago, IL United States Conference Start: 20130621 Conference End: 20130625 Conference Publication: (var pagings)* 2013;62(pp A148):July. doi: http://dx.doi.org/10.2337/db13-388-679
26. Eze CO, Onwuekwe IO, Agu CE, et al. The prevalence of orthostatic hypotension in type 2 diabetes mellitus patients in a diabetic clinic in Enugu South-East Nigeria. *Nigerian journal of medicine : journal of the National Association of Resident Doctors of Nigeria* 2013;22(3):2013-2Sep.
27. Lanthier L, Touchette M, Bourget P, et al. [Evaluation of circadian variation of blood pressure by ambulatory blood pressure monitoring in an elderly diabetic population with or without orthostatic hypotension]. [French]. *Geriatrie et psychologie neuropsychiatrie du vieillissement* 2011;9(1):Mar.
28. Rota E, Quadri R, Fanti E, et al. Clinical and electrophysiological correlations in type 2 diabetes mellitus at diagnosis. *Diabetes Research and Clinical Practice* 2007;76(1):April. doi: http://dx.doi.org/10.1016/j.diabres.2006.07.027

29. Aghera D, McFadden C, Hunter K. Orthostatic hypotension in elderly individuals with chronic kidney disease(CKD). *American Journal of Kidney Diseases Conference: National Kidney Foundation 2015 Spring Clinical Meetings, NKF SCM15 Dallas, TX United States Conference Start: 20150325 Conference End: 20150329 Conference Publication: (var pagings)* 2015;65(4):April.
30. Bhat S, Hegde S, Szpunar S, et al. Prevalence of orthostatic variation in blood pressure among stable outpatient chronic kidney disease population. *American Journal of Kidney Diseases Conference: National Kidney Foundation 2013 Spring Clinical Meetings Orlando, FL United States Conference Start: 20130402 Conference End: 20130406 Conference Publication: (var pagings)* 2013;61(4):April. doi: http://dx.doi.org/10.1053/j.ajkd.2013.02.049
31. Phipps MS, Schmid AA, Kapoor JR, et al. Orthostatic hypotension among outpatients with ischemic stroke. *Journal of the Neurological Sciences* 2012;314(1-2):15. doi: http://dx.doi.org/10.1016/j.jns.2011.10.031
32. Ryan DJ, Kenny RA, Christensen S, et al. Ischaemic stroke or TIA in older subjects associated with impaired dynamic blood pressure control in the absence of severe large artery stenosis. *Age and Ageing* 2015;44(4):afv011. doi: http://dx.doi.org/10.1093/ageing/afv011
33. Eguchi K, Kario K, Hoshide S, et al. Greater change of orthostatic blood pressure is related to silent cerebral infarct and cardiac overload in hypertensive subjects. *Hypertension Research* 2004;27(4):April. doi: http://dx.doi.org/10.1291/hypres.27.235
34. Kario K, Shimada K. Orthostatic blood pressure changes and silent cerebrovascular disease. *Cardiology Review* 2003;20(4):01.
35. Kwok CS, Ong ACL, Potter JF, et al. TIA, stroke and orthostatic hypotension: A disease spectrum related to ageing vasculature? *International Journal of Clinical Practice* 2014;68(6):June. doi: http://dx.doi.org/10.1111/ijcp.12373
36. Rutan GH, Hermanson B, Bild DE, et al. Orthostatic hypotension in older adults. The Cardiovascular Health Study. CHS Collaborative Research Group. *Hypertension* 1992;19(6:Pt 1):t-19.
37. Lin ZQ, Pan CM, Li WH, et al. [The correlation between postural hypotension and myocardial infarction in the elderly population]. [Chinese]. *Zhonghua nei ke za zhi [Chinese journal of internal medicine]* 2012;51(7):Jul.
38. Potocka-Plazak K, Plazak W. Orthostatic hypotension in elderly women with congestive heart failure. *Aging - Clinical and Experimental Research* 2001;13(5):2001.
39. McGann PE. Comorbidity in heart failure in the elderly. *Clinics in Geriatric Medicine* 2000;16(3):2000.
40. Rascol O, Perez-Lloret S, Damier P, et al. Falls in ambulatory non-demented patients with Parkinson's disease. *Journal of Neural Transmission* 2015;122(10):07. doi: http://dx.doi.org/10.1007/s00702-015-1396-2
41. Senard JM, Rai S, Lapeyre-Mestre M, et al. Prevalence of orthostatic hypotension in Parkinson's disease. *Journal of Neurology Neurosurgery and Psychiatry* 1997;63(5):November.
42. Palma J-A, Gomez-Esteban JC, Norcliffe-Kaufmann L, et al. Orthostatic Hypotension in Parkinson Disease: How Much You Fall or How Low You Go? *Movement Disorders* 2015;30(5):15. doi: http://dx.doi.org/10.1002/mds.26079
43. Elmstahl S, Widerstrom E. Orthostatic intolerance predicts mild cognitive impairment: incidence of mild cognitive impairment and dementia from the Swedish general population cohort Good Aging in Skane. *Clinical interventions in aging* 2014;9(pp 1993-2002):2014. doi: http://dx.doi.org/10.2147/CIA.S72316
44. Frewen J, Savva GM, Boyle G, et al. Cognitive performance in orthostatic hypotension: Findings from a nationally representative sample. *Journal of the American Geriatrics Society* 2014;62(1):January. doi: http://dx.doi.org/10.1111/jgs.12592
45. Frewen J, Finucane C, Savva GM, et al. Orthostatic hypotension is associated with lower cognitive performance in adults aged 50 plus with supine hypertension. *The journals of gerontology*

Series A, Biological sciences and medical sciences 2014;69(7):Jul. doi: http://dx.doi.org/10.1093/gerona/glt171

46. Traykova M, Stankova T, Mehrabian S, et al. High prevalence of orthostatic hypotension in vascular and degenerative dementia. *European Journal of Medical Research Conference: 21st European Students' Conference Promising Medical Scientists Willing to Look Beyond Berlin Germany Conference Start: 20101013 Conference End: 20101017 Conference Publication: (var pagings)* 2010;15(pp 126-127):13.
47. Yap PL, Niti M, Yap KB, et al. Orthostatic hypotension, hypotension and cognitive status: early comorbid markers of primary dementia? *Dementia & Geriatric Cognitive Disorders* 2008;26(3):239-46. doi: http://dx.doi.org/10.1159/000160955
48. Sonnesyn H, Nilsen DW, Rongve A, et al. High prevalence of orthostatic hypotension in mild dementia. *Dementia and Geriatric Cognitive Disorders* 2009;28(4):November. doi: http://dx.doi.org/10.1159/000247586
49. Campbell AJ, Reinken J. Postural hypotension in old age: Prevalence, associations and prognosis. *Journal of Clinical and Experimental Gerontology* 1985;7(2):1985.
50. Isik AT, Soysal P, Mas M. Orthostatic hypotension and long-term effects of acheis on the orthostatic hypotension in elderly patients with alzheimer disease. *Alzheimer's and Dementia Conference: Alzheimer's Association International Conference 2014 Copenhagen Denmark Conference Start: 20140712 Conference End: 20140717 Conference Publication: (var pagings)* 2014;10(pp P774):July.
51. Regan CO, Kearney PM, Cronin H, et al. Oscillometric measure of blood pressure detects association between orthostatic hypotension and depression in population based study of older adults. *BMC psychiatry* 2013;13(pp 266):2013. doi: http://dx.doi.org/10.1186/1471-244X-13-266
52. Ruwald MH, Hansen ML, Lamberts M, et al. Comparison of incidence, predictors, and the impact of co-morbidity and polypharmacy on the risk of recurrent syncope in patients <85 versus >85 years of age. *American Journal of Cardiology* 2013;112(10):15. doi: http://dx.doi.org/10.1016/j.amjcard.2013.07.041
53. Pepersack T, Gilles C, Petrovic M, et al. Prevalence of orthostatic hypotension and relationship with drug use amongst older patients. *Acta clinica Belgica* 2013;68(2):2013-2Apr.
54. Kobayashi K, Yamada S. Development of a simple index, calf mass index, for screening for orthostatic hypotension in community-dwelling elderly. *Archives of Gerontology and Geriatrics* 2012;54(2):March. doi: http://dx.doi.org/10.1016/j.archger.2011.04.003
55. Di SC, Milazzo V, Bruno G, et al. Prevalence of orthostatic hypotension in a cohort of patients under antihypertensive therapy. *High Blood Pressure and Cardiovascular Prevention Conference: 2013 National Congress of the Italian Society of Hypertension, SIIA 2013 Rome Italy Conference Start: 20131003 Conference End: 20131005 Conference Publication: (var pagings)* 2013;20(3):September. doi: http://dx.doi.org/10.1007/s40292-013-0021-4
56. Rozenfeld S, Bastos Camacho LA, Peixoto VR. Medication as a risk factor for falls in older women in Brazil. *Revista Panamericana de Salud Publica/Pan American Journal of Public Health* 2003;13(6):01.
57. Soysal P, Yay A, Isik AT. Does 25-hydroxyvitamin D deficiency increase orthostatic hypotension risk in the elderly patients? *European Geriatric Medicine Conference: 10th International Congress of the European Union Geriatric Medicine Society - Geriatric Medicine Crossing Borders, EUGMS 2014 Rotterdam Netherlands Conference Start: 20140917 Conference End: 20140919 Conference* 2014;5(pp S120):September.
58. Veronese N, Bolzetta F, De RM, et al. Serum 25-hydroxyvitamin D and orthostatic hypotension in old people: The Pro.V.A. study. *Hypertension* 2014;64(3):September. doi: http://dx.doi.org/10.1161/HYPERTENSIONAHA.114.03143

59. O'Connell MDL, Savva GM, Fan CW, et al. Orthostatic hypotension, orthostatic intolerance and frailty: The Irish Longitudinal Study on Aging-TILDA. *Archives of Gerontology and Geriatrics* 2015;60(3):01. doi: http://dx.doi.org/10.1016/j.archger.2015.01.008
60. Rockwood MR, Howlett SE, Rockwood K. Orthostatic hypotension (OH) and mortality in relation to age, blood pressure and frailty. *Archives of Gerontology & Geriatrics* 2012;54(3):e255-e60. doi: http://dx.doi.org/10.1016/j.archger.2011.12.009
61. Lagro J, Laurenssen NCW, Schalk BWM, et al. Diastolic blood pressure drop after standing as a clinical sign for increased mortality in older falls clinic patients. *Journal of Hypertension* 2012;30(6):June. doi: http://dx.doi.org/10.1097/HJH.0b013e328352b9fd
62. Madhavan G, Goddard AA, McLeod KJ. Prevalence and Etiology of Delayed Orthostatic Hypotension in Adult Women. *Archives of Physical Medicine and Rehabilitation* 2008;89(9):September. doi: http://dx.doi.org/10.1016/j.apmr.2008.02.021
63. Weiss A, Beloosesky Y, Grinblat J, et al. Seasonal changes in orthostatic hypotension among elderly admitted patients. *Aging Clinical and Experimental Research* 2006;18(1):February.
64. Pathak A, Lapeyre-Mestre M, Montastruc J-L, et al. Heat-related morbidity in patients with orthostatic hypotension and primary autonomic failure. *Movement Disorders* 2005;20(9):September. doi: http://dx.doi.org/10.1002/mds.20571
65. Ooi WL, Barrett S, Hossain M, et al. Patterns of orthostatic blood pressure change and their clinical correlates in a frail, elderly population. *Journal of the American Medical Association* 1997;277(16):23.
66. Ward C, Kenny RA. Reproducibility of orthostatic hypotension in symptomatic elderly. *American Journal of Medicine* 1996;100(4):April. doi: http://dx.doi.org/10.1016/S0002-9343%2897%2989517-4
67. Weiss A, Grossman E, Beloosesky Y, et al. Orthostatic hypotension in acute geriatric ward: Is it a consistent finding? *Archives of Internal Medicine* 2002;162(20):15. doi: http://dx.doi.org/10.1001/archinte.162.20.2369
68. Youde JH, Manktelow B, Ward-Close S, et al. Measuring postural changes in blood pressure in the healthy elderly. *Blood Pressure Monitoring* 1999;4(1):1999.

Medline and Embase Search Strategy

Date of search 20th October 2015

	Searches	Results
1	postural hypotension.mp. [mp=ti, ab, hw, tn, ot, dm, mf, dv, kw, nm, kf, px, rx, an, ui]	3109
2	orthostatic hypotension.mp. [mp=ti, ab, hw, tn, ot, dm, mf, dv, kw, nm, kf, px, rx, an, ui]	22694
3	1 or 2	21615
4	prevalence.mp. [mp=ti, ab, hw, tn, ot, dm, mf, dv, kw, nm, kf, px, rx, an, ui]	1202953
5	3 and 4	1678
6	limit 5 to humans	1565
7	limit 6 to aged <65+ years> [Limit not valid in Ovid MEDLINE(R),Ovid MEDLINE(R) In-Process; records were retained]	661
8	remove duplicates from 7	470
