# Supplementary material for: Detecting Risk Of Postural hypotension (DROP): derivation and validation of a prediction score for primary care
Source: BMJ Open. 2018 Apr 20;8(4):e020740. doi: 10.1136/bmjopen-2017-020740 (PMC5914723; doi:10.1136/bmjopen-2017-020740)
Supplement: Author's manuscript [file bmjopen-2017-020740.draft_revisions.pdf]

BMJ Open is committed to open peer review. As part of this commitment we make the peer review history of every article we publish publicly available.

When an article is published we post the peer reviewers' comments and the authors' responses online. We also post the versions of the paper that were used during peer review. These are the versions that the peer review comments apply to.

The versions of the paper that follow are the versions that were submitted during the peer review process. They are not the versions of record or the final published versions. They should not be cited or distributed as the published version of this manuscript.

BMJ Open is an open access journal and the full, final, typeset and author-corrected version of record of the manuscript is available on our site with no access controls, subscription charges or pay-per-view fees (http://bmjopen.bmj.com).

If you have any questions on BMJ Open's open peer review process please email info.bmjopen@bmj.com

BMJ Open

Detecting Risks Of Postural Hypotension (DROP): derivation and validation of a prediction score for primary care

Journal:	*BMJ Open*
Manuscript ID	bmjopen-2017-020740
Article Type:	Research
Date Submitted by the Author:	21-Nov-2017
Complete List of Authors:	Clark, Christopher; University of Exeter, Primary Care Research Group<br>Thomas, Daniel; University of Exeter, Primary Care Research Group<br>Warren, Fiona; University of Exeter Medical School, Institute of Health Research<br>Llewellyn, David; University of Exeter, Mental Health Research Group<br>Ferrucci, Luigi; Harbor Hospital, Baltimore, National Institute on Aging NIA-ASTRA Unit<br>Campbell, John; University of Exeter, Primary Care;
**Primary Subject Heading**:	General practice / Family practice
Secondary Subject Heading:	Cardiovascular medicine, Diagnostics, General practice / Family practice
Keywords:	Hypertension < CARDIOLOGY, PRIMARY CARE, PREVENTIVE MEDICINE

SCHOLARONE™
Manuscripts

For peer review only

Detecting Risks Of Postural Hypotension (DROP): derivation and validation of a prediction score for primary care

Clark CE, Thomas D, Warren F, Llewellyn D, Ferrucci L, Campbell JL

Christopher E Clark[1], Daniel Thomas[1], Fiona C Warren[1], David J. Llewellyn[2], Luigi Ferrucci[3], John L Campbell[1]

1. Primary Care Research Group
Institute of Health Research
University of Exeter Medical School
Smeall Building, St Luke's Campus
Magdalen Rd, Exeter, Devon, England EX1 2LU

2. Mental Health Research Group
Institute of Health Research
University of Exeter Medical School
College House, St Luke's Campus
Magdalen Rd, Exeter, Devon, England EX1 2LU

3. National Institute on Aging, Baltimore, Maryland, USA
251 Bayview Blvd. Room 04C228
Baltimore, MD 21224 - USA

Abstract: 296 words
Text: 3040 words
Tables: 5
Figures: 3
Appendices: 1

Address for correspondence: Dr C E Clark, as above, email: c.e.clark@exeter.ac.uk

For peer review only - http://bmjopen.bmj.com/site/about/guidelines.xhtml

Abstract

Objectives

Falls are a common problem in older people. Postural hypotension contributes to falls but is often asymptomatic. In the absence of symptoms, postural hypotension is only infrequently checked for in clinical practice. We undertook this study to derive, validate and explore the prospective associations of a prediction tool to identify people likely to have unrecognised postural hypotension.

Design and setting

Cross-sectional and prospective multivariable cohort analysis.

Participants

1317 participants of the InCHIANTI study, a population based cohort representative of the older Italian population.

Primary outcome measures

Predictive value of score to suggest presence of postural hypotension,

Methods

Subjects were randomised 1:1 to derivation or validation cohorts. Within the derivation cohort univariable associations for candidate predictors of postural hypotension were tested. Variables with P<0.1 entered multivariable linear regression models. Factors retaining multivariable significance were incorporated into unweighted and weighted DROP scores. These scores were tested in the validation cohort against prediction of postural hypotension, cognitive decline and mortality over nine years' follow up.

Results

Postural hypotension was present in 203 (15.4%) of participants. Factors predicting postural hypotension were: digoxin use, Parkinson's disease, hypertension, stroke or cardiovascular disease, and an inter-arm systolic blood pressure difference. Area under the curve was consistent at 0.65 for all models, with significant odds ratios (OR) of 1.8 to 2.4 per unit increase in score for predicting postural hypotension. For a DROP score ≥1, five cases need to be tested to identify one with postural hypotension.

Increasing DROP scores predicted mortality (OR 1.8 to 2.8 per unit rise) and increasing rates of decline of Mini Mental State Examination score (ANOVA p<0.001) over 9 years of follow up.

Conclusions

The DROP score provides a simple method to identify people likely to have postural hypotension, and increased risks to health, who require further evaluation.

(296 words)

For peer review only

For peer review only - http://bmjopen.bmj.com/site/about/guidelines.xhtml

Strengths and limitations of this study

- This study, to derive and validate a score ("DROP score") predicting the presence of postural hypotension, was undertaken using data from a well-established cohort representative of a free living older population in Italy. Comprehensive recording of baseline variables by the InCHIANTI investigators allowed a large number of previously reported risk markers for PH to be tested in the analyses.

- The study was undertaken according to TRIPOD guidelines and randomised splitting of the cohort allowed internal validation of the findings to be undertaken.

- The findings confirmed that a simple risk marker based score can predict who may benefit from testing for postural hypotension. Sensitivity, specificity and area under the curve results were modest; the study requires replication in a larger cohort before implementation of the DROP score can be recommended in practice.

- Guidelines advise testing for postural hypotension in the elderly and those with diabetes. Neither of these risk markers appeared significant in the current analyses. Refinement of the scoring system within a larger cohort more representative of the older UK population is planned to explore this further.

For peer review only

For peer review only - http://bmjopen.bmj.com/site/about/guidelines.xhtml

Introduction

Falls are a major cause of morbidity and mortality in older people; 35% of people older than 65 and 50% of people older than 80 fall at least once a year.[1 2] Falls are the leading cause of disability and the leading cause of death from injury among people over 75 in the UK, and cost the NHS around £2.3 billion per year.[3] Postural or orthostatic hypotension is a major risk factor for falls,[4 5] and is independently associated with increased mortality rates.[6-8] Postural hypotension has also been associated with dementia and cognitive impairment, and may have more subtle adverse effects on wellbeing and cognition.[9]

Postural hypotension is commonly defined as a fall of either ≥20mmHg in systolic blood pressure or ≥10mmHg in diastolic blood pressure, from sitting or lying, within three minutes of standing up.[10] Reported prevalences of postural hypotension vary widely, and are sensitive to both care setting, occurring in over half of patients admitted to care of the elderly,[11-13] and to the presence of co-morbidity. General adult population prevalence appears to be around 7%,[14 15] rising to 11 to 15% in persons 65 years old and older,[16-18] and 19% in those aged over 80 or older.[15] Prevalence is reported to be higher in the presence of hypertension,[19-23] stroke,[24 25] myocardial infarction,[25 26] and diabetes.[22 27]

Guidelines vary in recommendations for the detection of postural hypotension. The National Institute for Health and Care Excellence (NICE) recommends testing in the presence of symptoms whist the European Society for Hypertension also recommends testing in the elderly and in the presence of diabetes.[1 28] Unfortunately most individuals with postural hypotension are asymptomatic,[7] and we have found that, in practice, postural hypotension is seldom looked for in patients who do not report postural symptoms.[29] Anecdotally, testing is not undertaken due to time constraints; screening for postural hypotension is not supported in the literature, being regarded as lacking an evidence base, and primary care workloads are rising. [30 31] Risks of hospitalisation, nursing home admission or mortality can already be predicted by the electronic frailty index (eFI), a score derived from existing information in primary care computer records, and incorporated into many general practice computing systems. However the association of eFI with, and its ability to predict, postural hypotension (which itself is poorly tested for and recorded in primary care) is unclear,[32] and comparable frailty indices have not been found to be predictive of postural hypotension.[33] To address this gap in care we hypothesised that a simple prediction score, based on easily recognised risk markers, might help clinicians identify those most likely to have postural hypotension thereby allowing a targeted implementation of sitting and standing blood pressure measurement in the absence of symptoms. We therefore undertook the current analysis, in a well-documented cohort known to be representative of an older population living in the community. Aims were to explore the feasibility of deriving and internally validating a prediction score, to assess its value and its prospective associations.

Methods

The study was conducted and reported in accordance with the TRIPOD statement.[34] We studied participants from the InCHIANTI study; a cohort study designed to explore declining mobility in later life. The Italian National Research Council on Aging ethical committee approved the InCHIANTI study protocol, and the current analysis proposals were approved by the investigating committee of the InCHIANTI study.

The InCHIANTI study methods have been described in detail elsewhere.[35] In brief, 1270 participants aged 65 years or more were randomly selected from the population registries of two villages: Greve

For peer review only - http://bmjopen.bmj.com/site/about/guidelines.xhtml

in Chianti, and Antella in Bagno a Ripoli. Additional people were randomly selected from these sites to complete recruitment of at least 30 men and 30 women for each age decile from age 20 to 29 upwards. Extensive baseline interviews and examinations were conducted at recruitment, between September 1998 and March 2000, and follow up data were obtained after three, six and nine years. blood pressure was initially measured supine, sequentially in both arms, to identify the higher reading arm, then a further two measurements were made on the higher reading arm. Subjects then stood and blood pressure was measured once after 1 minute and once more after 3 minutes standing. All measurements were obtained by research assistants using a standard mercury sphygmomanometer.

Baseline blood pressure was calculated as the mean of the second and third supine blood pressure readings.[36] Postural changes in blood pressure from lying to standing were calculated by subtraction of this mean from the standing blood pressure. Postural hypotension was considered to exist where there was as a reduction in blood pressure on standing of ≥20mmHg systolic or ≥10mmHg diastolic after 1 or after 3 minutes.[10] Hypertension was defined as use of antihypertensive drugs and/or a documented history of hypertension at recruitment.

For this analysis, participants were randomly allocated in a 1:1 ratio using a split-sample method,[37] stratified for gender and study site, to either a *derivation* or a *validation* group by a statistician (FW) blinded to postural hypotension status and medical history. A literature review was undertaken to identify potential risk markers for consideration in the analyses (appendix). These were mapped to variables available in the InCHIANTI dataset (table 1), which were then tested in the derivation cohort for univariable associations with postural hypotension, using t-tests or χ^2 tests as appropriate to the data. Variables signalling potential univariable associations (defined as p<0.1) were included in multivariable model analyses using an automated backward stepwise regression method.[38] We also included age (explored both continuously and as a dichotomous variable with cut-offs of 60, 65 and 70 years) and gender in all multivariable models. Prospective associations of postural hypotension with survival up to 9 years of follow-up were tested using Kaplan-Meier plots and Cox proportional hazard ratios. Cognitive decline was defined as a reduction in Mini Mental State Examination score (MMSE score) of 5 points or more from baseline, and rate of cognitive decline was defined as change in MMSE scores averaged per year of follow up.

Risk markers that retained significance in the multivariable models were used to derive both weighted and unweighted scores (DROP scores); weighted scores were derived by the addition of the multivariable Log (n) odds ratio (OR) for each marker present, whereas the unweighted model allocated one point for each risk marker present. Scores were tested in the validation cohort for ability to predict postural hypotension using ROC analysis, to predict future mortality using Cox proportional hazard ratios, and cognitive decline over nine years using ANOVA. All analyses were undertaken using IBM SPSS Statistics v24.0.0.2.

Results

Data for standing blood pressure existed for 1317 of the 1453 participants (91%) and they formed the cohort for this study. The derivation cohort (n=649) and validation cohort (n=668) were well matched for all important characteristics and putative risk markers (table 2); overall postural hypotension was present for 203 (15.4%) of participants at recruitment. Mean age of participants was 68.3 (standard deviation 15.5).

For peer review only - http://bmjopen.bmj.com/site/about/guidelines.xhtml

For the derivation cohort postural hypotension was associated, over 9 years of follow-up, with increased all-cause mortality (Hazard Ratio (HR) 1.9; 95% confidence interval (95%CI) 1.4 to 2.7), cardiovascular mortality (HR 2.1; 95%CI 1.2 to 3.4), and non-cardiovascular mortality (HR 2.0; 95%CI 1.3 to 3.0). Results of univariable testing are summarised in table 3. Using a cut off value of $p<0.1$ the following candidate predictors were entered into multivariable models: age (continuous, or dichotomous for age 60 or 70 cut offs), MMSE score, digoxin use, presence of hypertension, any cardiovascular disease (composite of history of myocardial infarction, angina pectoris or congestive heart failure), stroke, Parkinson's disease, hospital admission within the last year, WHO disability level, any disability in activities of daily living, systolic inter-arm difference (continuous or using ≥10mmHg cut off).

Backward stepwise regression analysis produced consistent findings with any permutation of discrete and continuous variables for age (which was not retained in any model) or for inter-arm difference (model 1 and model 2; table 4). Consequently, a dichotomous cut off for inter-arm difference of ≥10mmHg was selected for simplicity, and retained with five other factors to derive weighted (using log OR) and unweighted (score 1 for each factor present; possible range 0 to 6) *DROP* scores. The scores were tested in the validation cohort. Since inter-arm difference is not routinely measured a third model excluding inter-arm difference (model 3, table 4) was also used to derive DROP scores without this term (possible range 0 to 5).

All versions of the DROP score were found to predict postural hypotension in the validation cohort with similar areas under the curve of 0.65 but a trend to higher odds of postural hypotension with the exclusion of inter-arm difference from the model (Figure 1, table 5). Sensitivities and specificities of the unweighted DROP score without the inter-arm difference term were 76%, 16%, 5% and 53%, 91%, 99% respectively for cut-offs of ≥1, ≥2 and ≥3, although only 15 participants attained a DROP score of 3 and only one a score of 4. This equated to a number needed to test in order to detect one case of postural hypotension of 5, 5 and 2 for DROP scores of 1, 2 and 3 respectively. For the weighted DROP score without inter-arm difference a cut off value of 0.6 or more had a sensitivity of 74% and specificity of 55% for detection of postural hypotension. A similar pattern was seen for the DROP models including inter-arm difference; for an unweighted DROP score of 1 or more sensitivity and specificity for postural hypotension were 81% and 46% respectively predicting detection of one case of postural hypotension for every five tested. For the weighted score, a cut off value of 0.26 had a sensitivity of 81% and a specificity of 46% for detection of postural hypotension.

DROP scores were predictive of mortality over nine years of follow-up, with increasing ORs according to DROP score with adjustment for age (Figure 2). Classification by unweighted DROP scores was also predictive of declines in MMSE after nine years (Figure 3).

Discussion

Main findings

This analysis has confirmed that it is feasible, in a community living cohort of predominantly older people, to derive a score based on easily recognised risk markers, that can help to identify older persons that are likely to have postural hypotension and require further clinical evaluation. The score, consisting of six risk markers: use of digoxin, presence of Parkinson's disease, hypertension, cardiovascular disease, stroke, and a difference in systolic blood pressure between arms ≥10mmHg, performs similarly with or without weighting, therefore a simple additive score is preferred. Performance is also similar when the inter-arm term is omitted, further simplifying its application.

For peer review only - http://bmjopen.bmj.com/site/about/guidelines.xhtml

In this population, postural hypotension is associated with a doubling of risk of death over nine years of follow-up. The DROP score also predicts increasing future mortality from any cause and is associated with greater decline in Mini Mental State Examination scores.

Strengths and weaknesses

The cohort was chosen as representative of a free-living elderly population and the 15.4% prevalence of postural hypotension is consistent with figures ranging from 11 to 15% in other general elderly (over 65) populations.[16-18] Comprehensive recording of baseline variables allowed a large number of previously reported risk factors for postural hypotension to be tested. Since this was undertaken as a feasibility study no formal sample size calculation was undertaken, however there were sufficient events to support the multivariable analyses performed.[38] Although the relatively low numbers attaining DROP scores higher than 2 did lead to imprecision around the predictive values of those higher levels of scores. Re-analysis and external validation in a larger sized cohort could overcome this limitation. Blood pressures were measured supine and standing for this study whereas in practice sitting and standing measurements are commonly recommended.[36] These are less sensitive but more practical in primary care,[39] however a score derived in supine to standing cases of postural hypotension cannot be assumed to perform similarly in the sitting to standing setting. Therefore, we regard this analysis as a feasibility study that supports the concept of a simple pragmatic prediction score to aid daily practice, in need of refinement through larger scale analyses, and exploration in cohorts with sit to stand measurements.

Relevance to literature

Postural hypotension has previously been reported as a significant independent predictor of four year all-cause mortality in the Honolulu Heart programme.[6] It also predicted mortality in the Malmo Heart study,[8] but not in the Helsinki ageing study.[40] Frailty was associated with a higher prevalence of postural hypotension in the TILDA study, and adjustment for frailty may influence associations with mortality.[41 42] However no measures of frailty remained predictive of postural hypotension on inclusion in the current multivariable analyses, and a frailty index predicted postural *symptoms* but not postural hypotension within TILDA.[33]

Prevalence of postural hypotension rises with age.[15] Although those with postural hypotension in this study were on average five years older age was not a significant independent predictor of postural hypotension in our models. This may have been in part due to the skewed nature of the age profile in InCHIANTI, although sensitivity analyses excluding those under 65 made no difference (not reported). Prevalence of postural hypotension is elevated in association with a history of stroke or TIA,[43-45] cardiovascular disease,[24-26] diabetes,[22 27] or hypertension, which itself affects over 60% of the over 65 age group.[46] Thus the significant factors in our models were all age related conditions which seems the likely explanation for loss of age itself as an independent predictor due to collinearity. Parkinson's disease was the strongest predictor of postural hypotension in our analyses although, affecting only 1.1% of participants, it was also the least common factor. Postural hypotension has previously been reported to have prevalence approaching 50% in some groups of Parkinson's sufferers,[47 48] although only a third of those with postural hypotension report symptoms.[49]

The association of postural hypotension with presence of an inter-arm difference is, to our knowledge, a novel finding. We have previously associated inter-arm difference with white coat effects, which can confound detection of postural hypotension.[50 51] Arterial stiffness is a postulated cause of inter-arm difference,[52] and is also associated with postural hypotension;[53 54] thus inter-arm difference as a proxy measure of arterial stiffness might account for the observed association.

For peer review only - http://bmjopen.bmj.com/site/about/guidelines.xhtml

Although postural hypotension is associated with diabetes, and with other complications such as neuropathy, retinopathy and proteinuria,[55] there was no univariable association in this study. Prevalence of postural hypotension in diabetes is associated with complications and duration of disease;[56 57] in this cohort diabetes was present in only 6% of participants, whereas recent data suggest that 25% of adults over the age of 65 in the US have it.[58] Therefore a validation of our models in other larger representative populations is needed.

Postural hypotension has been associated with mild cognitive impariement.[59 60] and reduced cognitive performance.[61] Postural hypotension did not predict cognitive decline in a two year prospective study of older Finns,[62] but is predictive over longer follow up.[63] In the current analysis postural hypotension per se was not predictive of cognitive decline over nine years of follow up but the DROP score was. This seems plausible given that it includes a number of risk markers known to be associated with cognitive decline.

Relevance to clinical practice

Testing sitting (or lying) and standing blood pressure takes time and training. The skills of nurses measuring postural hypotension are variable when compared with guidelines;[64] incorrect arm positioning can underestimate postural hypotension,[65] and the alerting reaction can over-estimate it.[66] Early and accurate detection of postural hypotension is a pre-requisite to intervening with medication withdrawal to reduce postural blood pressure drops and their associated risks including falls. Currently symptoms appear to be the main trigger for testing.[29] This should continue, however, a tool to identify which *asymptomatic* patients to test may help to target additional testing to those most likely to benefit. A DROP score of one or more appears to have such potential, and may support proposals that individuals at elevated risk of postural hypotension should be tested.[67]

The strongly cardiovascular composition of the DROP score means that patients will commonly be taking antihypertensive drugs. Potential adverse effects of withdrawing antihypertensive medication to ameliorate postural hypotension are unclear, and medication withdrawal may concern clinicians, carers and patients. Risk of falls rises incrementally with each added orthostatic drug.[68] Prevalence of postural hypotension in hypertension is related to use of cardiovascular drugs (antihypertensive agents, vasodilators, diuretics),[69 70] alpha blockers,[71] and the number of antihypertensive drugs used,[72 73] and is associated with resistant or uncontrolled hypertension.[74 75] Successful treatment of blood pressure in the elderly is in fact associated with lower prevalence of postural hypotension,[76 77] but withdrawal of antihypertensive therapy improves postural hypotension.[78 79]

We retained Parkinson's disease in our models due to the strength of the association with postural hypotension, however, on clinical grounds, testing for postural hypotension would be better regarded as integral to any review in Parkinson's disease, given the high prevalence of postural hypotension in this condition.[49]

We sought to develop a pragmatic score to support busy clinicians, faced with a rising workload and increasingly multimorbid caseload.[31] Although measurement of blood pressure in both arms has become more frequent over time it is not part of a routine review.[29 80] Therefore we derived a DROP score omitting inter-arm difference, which performed with similar sensitivity and specificity. For the same reasons, we prefer the unweighted score as a practical aide memoire to recognition of the risk of postural hypotension.

Further research

This study has examined the feasibility of identifying whom should be tested with sitting and standing blood pressure measurements to detect asymptomatic postural hypotension. It seems that

For peer review only - http://bmjopen.bmj.com/site/about/guidelines.xhtml

a simple pragmatic scoring system can support this. We need to refine and externally validate this approach in larger samples more representative of UK primary care. Further work is needed to examine the feasibility and implications of medication review and antihypertensive withdrawal based on detection of postural hypotension in primary care.

Conclusion

We have described the derivation and validation of a score predicting the presence of postural hypotension. Initial testing suggests this approach to be feasible, and has identified the potential utility of the score in predicting mortality and cognitive decline over a nine year period of follow up. Further validation of the score in larger cohorts of individuals is warranted.

For peer review only

For peer review only - http://bmjopen.bmj.com/site/about/guidelines.xhtml

References

1. Falls: Assessment and prevention of falls in older people. London: National Institute for Health and Care Excellence 2013.
2. Blake AJ, Morgan K, Bendall MJ, et al. FALLS BY ELDERLY PEOPLE AT HOME: PREVALENCE AND ASSOCIATED FACTORS. *Age and Ageing* 1988;17(6):365-72. doi: 10.1093/ageing/17.6.365
3. The Importance of Vision in Preventing Falls. London: The College of Optometrists, British Geriatrics Society 2011.
4. Juraschek SP, Daya N, Appel LJ, et al. Orthostatic Hypotension in Middle-Age and Risk of Falls. *American Journal of Hypertension* 2017;30(2):188-95. doi: 10.1093/ajh/hpw108
5. McDonald C, Pearce M, Kerr SR, et al. A prospective study of the association between orthostatic hypotension and falls: Definition matters. *Age and Ageing* 2017;46(3):439-45. doi: http://dx.doi.org/10.1093/ageing/afw227
6. Masaki KH, Schatz IJ, Burchfiel CM, et al. Orthostatic hypotension predicts mortality in elderly men: The Honolulu Heart Program. *Circulation* 1998;98(21):24.
7. Benvenuto LJ, Krakoff LR. Morbidity and Mortality of Orthostatic Hypotension: Implications for Management of Cardiovascular Disease. *American Journal of Hypertension* 2011;24(2):135-44.
8. Fedorowski A, Stavenow L, Hedblad B, et al. Orthostatic hypotension predicts all-cause mortality and coronary events in middle-aged individuals (The Malmo Preventive Project). *European Heart Journal* 2010;31(1):85-91. doi: http://dx.doi.org/10.1093/eurheartj/ehp329
9. Mehrabian S, Duron E, Labouree F, et al. Relationship between orthostatic hypotension and cognitive impairment in the elderly. *J Neurol Sci* 2010;299(1-2):45-8. doi: 10.1016/j.jns.2010.08.056 [published Online First: 2010/09/22]
10. Freeman R, Wieling W, Axelrod FB, et al. Consensus statement on the definition of orthostatic hypotension, neurally mediated syncope and the postural tachycardia syndrome. *Clin Auton Res* 2011;21(2):69-72. doi: 10.1007/s10286-011-0119-5
11. Boland E, Brackelaire G, Anani Y, et al. Postprandial hypotension among patients from an acute geriatric ward: Results from a pilot study. *European Geriatric Medicine Conference: 11th International Congress of the European Union Geriatric Medicine Society, EUGMS 2015 Oslo Norway Conference Start: 20150916 Conference End: 20150918 Conference Publication: (var pagings)* 2015;6(pp S64):September.
12. Potocka-Plazak K, Plazak W. Orthostatic hypotension in elderly women with congestive heart failure. *Aging - Clinical and Experimental Research* 2001;13(5):2001.
13. McGann PE. Comorbidity in heart failure in the elderly. *Clinics in Geriatric Medicine* 2000;16(3):2000.
14. Vara-Gonzalez L, Munoz-Cacho P, De Castro SS. Postural changes in blood pressure in the general population of Cantabria (northern Spain). *Blood Pressure Monitoring* 2008;13(5):October. doi: http://dx.doi.org/10.1097/MBP.0b013e32830d4b33
15. Finucane C, O'Connell MD, Fan CW, et al. Age-related normative changes in phasic orthostatic blood pressure in a large population study: findings from The Irish Longitudinal Study on Ageing (TILDA). *Circulation* 2014;130(20):11. doi: http://dx.doi.org/10.1161/CIRCULATIONAHA.114.009831
16. Atli T, Keven K. Orthostatic hypotension in the healthy elderly. *Archives of Gerontology and Geriatrics* 2006;43(3):November/December. doi: http://dx.doi.org/10.1016/j.archger.2005.12.001
17. Mader SL, Josephson KR, Rubenstein LZ. Low prevalence of postural hypotension among community-dwelling elderly. *Journal of the American Medical Association* 1987;258(11):1987. doi: http://dx.doi.org/10.1001/jama.258.11.1511
18. Vara GL, Dominguez RR, Fernandez RM, et al. Prevalence of orthostatic hypotension in elderly hypertensive patients in primary care. [Spanish]. *Atencion primaria / Sociedad Espanola de Medicina de Familia y Comunitaria* 2001;28(3):2001-2Aug.

For peer review only - http://bmjopen.bmj.com/site/about/guidelines.xhtml

19. Shin C, Abbott RD, Lee H, et al. Prevalence and correlates of orthostatic hypotension in middle-aged men and women in Korea: The Korean Health and Genome Study. *Journal of Human Hypertension* 2004;18(10):October. doi: http://dx.doi.org/10.1038/sj.jhh.1001732
20. Saez T, Suarez C, Sierra MJ, et al. Orthostatic hypotension in the elderly and its association with the antihypertensive treatment. [Spanish]. *Medicina Clinica* 2000;114(14):15.
21. Harris T, Lipsitz LA, Kleinman JC, et al. Postural change in blood pressure associated with age and systolic blood pressure. The National Health and Nutrition Examination Survey II. *Journals of Gerontology* 1991;46(5):1991.
22. Wu JS, Yang YC, Lu FH, et al. Population-based study on the prevalence and risk factors of orthostatic hypotension in subjects with pre-diabetes and diabetes. *Diabetes Care* 2009;32(1):69-74. doi: http://dx.doi.org/10.2337/dc08-1389
23. Applegate WB, Davis BR, Black HR, et al. Prevalence of postural hypotension at baseline in the systolic hypertension in the elderly program (SHEP) cohort. *Journal of the American Geriatrics Society* 1991;39(11):1991.
24. Kwok CS, Ong ACL, Potter JF, et al. TIA, stroke and orthostatic hypotension: A disease spectrum related to ageing vasculature? *International Journal of Clinical Practice* 2014;68(6):June. doi: http://dx.doi.org/10.1111/ijcp.12373
25. Rutan GH, Hermanson B, Bild DE, et al. Orthostatic hypotension in older adults. The Cardiovascular Health Study. CHS Collaborative Research Group. *Hypertension* 1992;19(6:Pt 1):t-19.
26. Lin ZQ, Pan CM, Li WH, et al. [The correlation between postural hypotension and myocardial infarction in the elderly population]. [Chinese]. *Zhonghua nei ke za zhi [Chinese journal of internal medicine]* 2012;51(7):Jul.
27. Gupta A, Gilden J. Prevalence of diabetes in patients admitted to the hospital with primary diagnosis of orthostatic hypotension. *Diabetes Conference: 73rd Scientific Sessions of the American Diabetes Association Chicago, IL United States Conference Start: 20130621 Conference End: 20130625 Conference Publication: (var pagings)* 2013;62(pp A148):July. doi: http://dx.doi.org/10.2337/db13-388-679
28. Mancia G, Fagard R, Narkiewicz K, et al. 2013 ESH/ESC Guidelines for the management of arterial hypertension. *J Hypertens* 2013;31:1281-357.
29. Mejzner N, Clark CE, Smith LF, et al. Trends in the diagnosis and management of hypertension: repeated primary care survey in South West England. *British Journal of General Practice* 2017 doi: 10.3399/bjgp17X690461
30. Hale WA, Chambliss ML. Should primary care patients be screened for orthostatic hypotension? *The Journal of family practice* 1999;48(7):Jul.
31. Hobbs FD, Bankhead C, Mukhtar T, et al. Clinical workload in UK primary care: a retrospective analysis of 100 million consultations in England, 2007-14. *Lancet* 2016;387(10035):2323-30. doi: 10.1016/s0140-6736(16)00620-6 [published Online First: 2016/04/10]
32. Clegg A, Bates C, Young J, et al. Development and validation of an electronic frailty index using routine primary care electronic health record data. *Age and Ageing* 2016;45(3):353-60. doi: 10.1093/ageing/afw039
33. O'Connell MDL, Savva GM, Fan CW, et al. Orthostatic hypotension, orthostatic intolerance and frailty: The Irish Longitudinal Study on Aging-TILDA. *Archives of Gerontology and Geriatrics* 2015;60(3):507-13. doi: http://dx.doi.org/10.1016/j.archger.2015.01.008
34. Collins GS, Reitsma JB, Altman DG, et al. Transparent reporting of a multivariable prediction model for individual prognosis or diagnosis (TRIPOD): the TRIPOD statement. *BMJ : British Medical Journal* 2015;350 doi: 10.1136/bmj.g7594
35. Ferrucci L, Bandinelli S, Benvenuti E, et al. Subsystems contributing to the decline in ability to walk: bridging the gap between epidemiology and geriatric practice in the InCHIANTI study. *J AmGeriatrSoc* 2000;48(12):1618-25.

For peer review only - http://bmjopen.bmj.com/site/about/guidelines.xhtml

36. Daskalopoulou SS, Rabi DM, Zarnke KB, et al. The 2015 Canadian Hypertension Education Program recommendations for blood pressure measurement, diagnosis, assessment of risk, prevention, and treatment of hypertension. *Can J Cardiol* 2015;31(5):549-68. doi: 10.1016/j.cjca.2015.02.016
37. Steyerberg EW. Clinical prediction models : a practical approach to development, validation, and updating. New York ; London: Springer 2009.
38. Collett D. Modelling survival data in medical research. Boca Raton, Fla: Chapman & Hall/CRC 2003.
39. Cooke J, Carew S, O'Connor M, et al. Sitting and standing blood pressure measurements are not accurate for the diagnosis of orthostatic hypotension. *QJM: An International Journal of Medicine* 2009;102(5):335-39. doi: 10.1093/qjmed/hcp020
40. Tilvis RS, Hakala S-M, Valvanne J, et al. Postural hypotension and dizziness in a general aged population: A four- year follow-up of the Helsinki Aging Study. *Journal of the American Geriatrics Society* 1996;44(7):July.
41. Rockwood MR, Howlett SE, Rockwood K. Orthostatic hypotension (OH) and mortality in relation to age, blood pressure and frailty. *Archives of Gerontology & Geriatrics* 2012;54(3):e255-e60. doi: http://dx.doi.org/10.1016/j.archger.2011.12.009
42. O'Connell MDL, Savva GM, Fan CW, et al. Orthostatic hypotension, orthostatic intolerance and frailty: The Irish Longitudinal Study on Aging-TILDA. *Archives of Gerontology and Geriatrics* 2015;60(3):01. doi: http://dx.doi.org/10.1016/j.archger.2015.01.008
43. Phipps MS, Schmid AA, Kapoor JR, et al. Orthostatic hypotension among outpatients with ischemic stroke. *Journal of the Neurological Sciences* 2012;314(1-2):15. doi: http://dx.doi.org/10.1016/j.jns.2011.10.031
44. Eguchi K, Kario K, Hoshide S, et al. Greater change of orthostatic blood pressure is related to silent cerebral infarct and cardiac overload in hypertensive subjects. *Hypertension Research* 2004;27(4):April. doi: http://dx.doi.org/10.1291/hypres.27.235
45. Kario K, Shimada K. Orthostatic blood pressure changes and silent cerebrovascular disease. *Cardiology Review* 2003;20(4):01.
46. Falaschetti E, Mindell J, Knott C, et al. Hypertension management in England: a serial cross-sectional study from 1994 to 2011. *Lancet* 2014;383(9932):1912-19.
47. Rascol O, Perez-Lloret S, Damier P, et al. Falls in ambulatory non-demented patients with Parkinson's disease. *Journal of Neural Transmission* 2015;122(10):07. doi: http://dx.doi.org/10.1007/s00702-015-1396-2
48. Senard JM, Rai S, Lapeyre-Mestre M, et al. Prevalence of orthostatic hypotension in Parkinson's disease. *Journal of Neurology Neurosurgery and Psychiatry* 1997;63(5):November.
49. Palma J-A, Gomez-Esteban JC, Norcliffe-Kaufmann L, et al. Orthostatic Hypotension in Parkinson Disease: How Much You Fall or How Low You Go? *Movement Disorders* 2015;30(5):15. doi: http://dx.doi.org/10.1002/mds.26079
50. Schwartz CL, Clark C, Koshiaris C, et al. Interarm Difference in Systolic Blood Pressure in Different Ethnic Groups and Relationship to the "White Coat Effect": A Cross-Sectional Study. *Am J Hypertens* 2017 doi: 10.1093/ajh/hpx073
51. Kamaruzzaman S, Watt H, Carson C, et al. The association between orthostatic hypotension and medication use in the British Women's Heart and Health Study. *Age and Ageing* 2010;39(1):51-56. doi: http://dx.doi.org/10.1093/ageing/afp192
52. Clark CE. The interarm blood pressure difference: Do we know enough yet? *The Journal of Clinical Hypertension* 2017;19(5):462-65. doi: 10.1111/jch.12982
53. Liu K, Wang S, Wan S, et al. Arterial Stiffness, Central Pulsatile Hemodynamic Load, and Orthostatic Hypotension. *Journal of Clinical Hypertension* 2016;18(7):655-62. doi: http://dx.doi.org/10.1111/jch.12726

For peer review only - http://bmjopen.bmj.com/site/about/guidelines.xhtml

54. Meng Q, Wang S, Wang Y, et al. Arterial stiffness is a potential mechanism and promising indicator of orthostatic hypotension in the general population. *Vasa - European Journal of Vascular Medicine* 2014;43(6):423-32. doi: http://dx.doi.org/10.1024/0301-1526/a000389
55. Eze CO, Onwuekwe IO, Agu CE, et al. The prevalence of orthostatic hypotension in type 2 diabetes mellitus patients in a diabetic clinic in Enugu South-East Nigeria. *Nigerian journal of medicine : journal of the National Association of Resident Doctors of Nigeria* 2013;22(3):2013-2Sep.
56. Lanthier L, Touchette M, Bourget P, et al. [Evaluation of circadian variation of blood pressure by ambulatory blood pressure monitoring in an elderly diabetic population with or without orthostatic hypotension]. [French]. *Geriatrie et psychologie neuropsychiatrie du vieillissement* 2011;9(1):Mar.
57. Rota E, Quadri R, Fanti E, et al. Clinical and electrophysiological correlations in type 2 diabetes mellitus at diagnosis. *Diabetes Research and Clinical Practice* 2007;76(1):April. doi: http://dx.doi.org/10.1016/j.diabres.2006.07.027
58. Kirkman MS, Briscoe VJ, Clark N, et al. Diabetes in Older Adults. *Diabetes Care* 2012;35(12):2650-64. doi: 10.2337/dc12-1801
59. Elmstahl S, Widerstrom E. Orthostatic intolerance predicts mild cognitive impairment: incidence of mild cognitive impairment and dementia from the Swedish general population cohort Good Aging in Skane. *Clinical interventions in aging* 2014;9(pp 1993-2002):2014. doi: http://dx.doi.org/10.2147/CIA.S72316
60. Frewen J, Savva GM, Boyle G, et al. Cognitive performance in orthostatic hypotension: Findings from a nationally representative sample. *Journal of the American Geriatrics Society* 2014;62(1):January. doi: http://dx.doi.org/10.1111/jgs.12592
61. Frewen J, Finucane C, Savva GM, et al. Orthostatic hypotension is associated with lower cognitive performance in adults aged 50 plus with supine hypertension. *The journals of gerontology Series A, Biological sciences and medical sciences* 2014;69(7):Jul. doi: http://dx.doi.org/10.1093/gerona/glt171
62. Viramo P, Luukinen H, Koski K, et al. Orthostatic hypotension and cognitive decline in older people. *Journal of the American Geriatrics Society* 1999;47(5):May.
63. Wolters FJ, Mattace-Raso FUS, Koudstaal PJ, et al. Orthostatic Hypotension and the Long-Term Risk of Dementia: A Population-Based Study. *PLoS Medicine* 2016;13 (10) (no pagination)(e1002143) doi: http://dx.doi.org/10.1371/journal.pmed.1002143
64. Vloet LCM, Smits R, Frederiks CMA, et al. Evaluation of skills and knowledge on orthostatic blood pressure measurements in elderly patients. *Age and Ageing* 2002;31(3):2002. doi: http://dx.doi.org/10.1093/ageing/31.3.211
65. Netea RT, Elving LD, Lutterman JA, et al. Body position and blood pressure measurement in patients with diabetes mellitus. *Journal of Internal Medicine* 2002;251(5):2002. doi: http://dx.doi.org/10.1046/j.1365-2796.2002.00958.x
66. Mo R, Omvik P, Lund-Johansen P. The Bergen Blood Pressure Study. Estimated prevalence of postural hypotension is influenced by the alerting reaction to blood pressure measurement. *Journal of Human Hypertension* 1994;8(3):1994.
67. Hale WA, Chambliss ML. Should primary care patients be screened for orthostatic hypotension? *The Journal of family practice* 1999;48(7):547-52.
68. Ruwald MH, Hansen ML, Lamberts M, et al. Comparison of incidence, predictors, and the impact of co-morbidity and polypharmacy on the risk of recurrent syncope in patients <85 versus >85 years of age. *American Journal of Cardiology* 2013;112(10):15. doi: http://dx.doi.org/10.1016/j.amjcard.2013.07.041
69. Pepersack T, Gilles C, Petrovic M, et al. Prevalence of orthostatic hypotension and relationship with drug use amongst older patients. *Acta clinica Belgica* 2013;68(2):2013-2Apr.

For peer review only - http://bmjopen.bmj.com/site/about/guidelines.xhtml

70. Poon IO, Braun U. High prevalence of orthostatic hypotension and its correlation with potentially causative medications among elderly veterans. *Journal of Clinical Pharmacy and Therapeutics* 2005;30(2):April. doi: http://dx.doi.org/10.1111/j.1365-2710.2005.00629.x
71. Kobayashi K, Yamada S. Development of a simple index, calf mass index, for screening for orthostatic hypotension in community-dwelling elderly. *Archives of Gerontology and Geriatrics* 2012;54(2):March. doi: http://dx.doi.org/10.1016/j.archger.2011.04.003
72. Di SC, Milazzo V, Bruno G, et al. Prevalence of orthostatic hypotension in a cohort of patients under antihypertensive therapy. *High Blood Pressure and Cardiovascular Prevention Conference: 2013 National Congress of the Italian Society of Hypertension, SIIA 2013 Rome Italy Conference Start: 20131003 Conference End: 20131005 Conference Publication: (var pagings)* 2013;20(3):September. doi: http://dx.doi.org/10.1007/s40292-013-0021-4
73. Kamaruzzaman S, Watt H, Carson C, et al. The association between orthostatic hypotension and medication use in the British Women's Heart and Health Study. *Age and Ageing* 2010;39(1):afp192.
74. Barochiner J, Alfie J, Aparicio LS, et al. Prevalence and clinical profile of resistant hypertension among treated hypertensive subjects. *Clinical and Experimental Hypertension* 2013;35(6):2013. doi: http://dx.doi.org/10.3109/10641963.2012.739236
75. Bouhanick B, Meliani S, Doucet J, et al. Orthostatic hypotension is associated with more severe hypertension in elderly autonomous diabetic patients from the French Gerodiab study at inclusion. *Annales de Cardiologie et d Angeiologie* 2014;63(3):176-82. doi: http://dx.doi.org/10.1016/j.ancard.2014.05.013
76. Auseon A, Ooi WL, Hossain M, et al. Blood pressure behavior in the nursing home: Implications for diagnosis and treatment of hypertension. *Journal of the American Geriatrics Society* 1999;47(3):March.
77. Masuo K, Mikami H, Ogihara T, et al. Changes in frequency of orthostatic hypotension in elderly hypertensive patients under medications. *American Journal of Hypertension* 1996;9(3):March. doi: http://dx.doi.org/10.1016/0895-7061%2895%2900348-7
78. Fotherby MD, Potter JF. Orthostatic hypotension and anti-hypertensive therapy in the elderly. *Postgraduate Medical Journal* 1994;70(830):1994.
79. Fotherby MD, Robinson TG, Potter JF. Clinic and 24 h blood pressure in elderly treated hypertensives with postural hypotension. *Journal of Human Hypertension* 1994;8(9):1994.
80. Parker E, Glasziou P. Use of evidence in hypertension guidelines: should we measure in both arms? *British Journal of General Practice* 2009;59:e87-e92. doi: 10.3399/bjgp09X395012

For peer review only

For peer review only - http://bmjopen.bmj.com/site/about/guidelines.xhtml

Authors' contributions

CEC conceived and undertook this analysis. DT contributed to the analysis. FW contributed to the analysis and offered statistical advice and support. DL offered advice on analysis and interpretation of cognitive impairment indices. LF supported the study on behalf of the InCHIANTI investigators. JLC supervised study conduct. CEC drafted the manuscript, all authors revised and edited the manuscript and all authors have read, reviewed, and approved the final manuscript.

Funding statement

CEC is supported by an NIHR Clinical Lectureship award. The views expressed are those of the authors and not necessarily those of the NIHR, the NHS or the Department of Health. This work was supported in part by the Intramural Research Program of the National Institute on Aging, National Institute of Health, Baltimore MD21224, USA.

Competing interests statement

All authors assert that they have no competing interests to declare

Data sharing statement

The InCHIANTI datasets are available on application with a research proposal to the InCHIANTI investigators at http://inchiantistudy.net/wp/

Previous dissemination

Interim reports on this work have been presented at annual scientific meetings of the European Society for Hypertension, Paris 2016 (Clark C, Thomas D, Warren F et al. Predicting postural hypotension, falls, and cognitive impairment: the InCHIANTI study. *J Hypertens* 2016; 34, e-Supplement 2: e32, September 2016) and the British and Irish Hypertension Society, Dublin, 2016 (Clark C, Thomas D, Mejzner N, et al. Can we predict who should be tested for postural hypotension? Derivation and validation of a prediction tool. *Journal of Human Hypertension* 2016;30 doi: doi:10.1038/jhh.2016.60)

For peer review only

For peer review only - http://bmjopen.bmj.com/site/about/guidelines.xhtml

Legends for tables and figures

Table 1. Risk markers included in univariable analysis

Table 2. Baseline characteristics of derivation and validation cohorts

Table 3. Univariable associations of risk markers with postural hypotension in derivation cohort

Table 4. Multivariable prediction models for postural hypotension

Table 5. DROP score associations with postural hypotension, mortality and cognitive decline

Figure 1. Prevalence of postural hypotension vs DROP Score.

Figure 2. Kaplan Meier survival plot for DROP scores over 9 years follow up

Figure 3. Mean change in Mini Mental State Examination score over nine years per DROP score

For peer review only

For peer review only - http://bmjopen.bmj.com/site/about/guidelines.xhtml

**Group**	**Risk marker included in analysis**
Demographics	age
	gender
Medical History	hypertension
	heart failure
	myocardial infarction
	angina
	stroke
	diabetes
	Parkinson’s disease
	cancer
	dementia
Examination	Mini Mental State Examination
Medications	antiarrhythmics
	antidepressants
	antipsychotics
	anxiolytics
	anticholinesterase inhibitors
Frailty	hospital admission, fall, or weight loss in last 12 months
	WHO physical disability level
	ADL disability score

Table 1. Risk markers included in univariable analysis

For peer review only

For peer review only - http://bmjopen.bmj.com/site/about/guidelines.xhtml

	Derivation Cohort	Validation Cohort	p
**N**	**649**	**668**	
	**Mean (SD) or N/%**	**Mean (SD) or N/%**	**t/ χ^2**
**age**	68.5 (15.7)	68.2 (15.3)	0.77
**BMI**	27.2 (4.3)	27.1 (4.0)	0.59
**Supine SBP (higher arm)#**	145.9 (21.3)	146.3 (21.6)	0.76
**Supine DBP (higher arm)#**	82.9 (8.8)	83.1 (9.5)	0.59
**Standing SBP 1 min**	140.4 (21.0)	141.2 (21.3)	0.51
**Standing DBP 1 min**	83.0 (8.9)	83.6 (9.4)	0.25
**Standing SBP 3 min**	141.4 (20.9)	141.9 (20.9)	0.66
**Standing DBP 3 min**	82.7 (9.0)	83.0 (9.4)	0.60
**Female**	368 (56.7)	358 (53.6)	0.27
**Site (Greve vs Bagno a Ripoli)**	320 vs 329	327 vs 341	0.91
**Deceased @ 9 years**	199 (30.7)	203 (30.4)	0.95
**Systolic drop ≥20mmHg 1min**	56 (8.6)	45 (6.7)	0.21
**Diastolic drop ≥10mmHg 1min**	41 (6.3)	40 (6.0)	0.82
**Systolic drop ≥20mmHg 3 min**	47 (7.2)	42 (6.3)	0.51
**Diastolic drop ≥10mmHg 3min**	46 (7.1)	48 (7.2)	1.00
**Postural Hypotension present***	107 (16.5)	96 (14.4)	0.32
**Systolic inter-arm difference ≥10mmHg**	121 (18.8)	121 (18.1)	0.83
**Previous stroke**	44 (6.8)	45 (6.7)	1.00
**Pre-existing diabetes**	80 (12.3)	76 (11.4)	0.61
**Pre-existing hypertension**	279 (43.0)	292 (43.7)	0.82
**Pre-existing CV disease**	63 (9.7)	50 (7.5)	0.17
**Pre-existing dementia**	38 (5.9)	27 (4.0)	0.16
**Pre-existing Parkinson's disease**	9 (1.4)	6 (0.9)	0.45
**Fall in preceding 12 months**	143 (22.0)	130 (19.5)	0.28

#mean of 2nd and 3rd readings
*defined as a drop of ≥20mmHg systolic or ≥10mmHg diastolic within 3 minutes of standing

Table 2. Baseline characteristics of derivation and validation cohorts

For peer review only

Variable (n (%) unless otherwise stated)	PH absent (n=542)	PH present (n=107)	p value
Age (mean, SD)	67.7 (15.8)	72.2 (14.6)	0.005
Age over 60	438 (81)	96 (90)	0.027
Age over 65	421 (78)	90 (84)	0.160
Age over 70	302 (56)	73 (68)	0.018
MMSE score (mean, SD)	25.3 (4.9)	24.1 (5.1)	0.031
Female gender	301 (55.5)	67 (62.6)	0.200
Digoxin	27	14	0.004
Antiarrhythmics, class I and III	10	4	0.264
Psycholeptics: typical antipsychotics	8	4	0.119
Psycholeptics: atypical antipsychotics	6	1	1.000
Psycholeptics: anxiolytics	103	18	0.684
Psychoanaleptics: antidepressants	22	5	0.791
Drugs for dementia	5	0	1.000
Hypertension	217	62	0.001
Congestive heart failure	22	10	0.028
Myocardial infarction	23	6	0.607
Angina	21	7	0.421
Any CV disease	45	18	0.011
Stroke	28	16	0.001
Diabetes	64	16	0.420
Parkinson's disease	4	5	0.008
Any cancer	30 (5.5)	8 (7.5)	0.497
Dementia	29	9	0.257
MMSE score 22 to 26	150	27	0.637
hospital admission in past year	54	18	0.044
Weight loss ≥10lbs in past year	22	7	0.301
Any fall in past year	115	28	0.254
Any ADL disability	100 (18.5)	28 (26.2)	0.083
WHO disability level >1	66 (12.2)	24 (22.6)	0.045
Systolic inter-arm difference (mean, SD) mmHg	2.0 (4.1)	4.7 (5.9)	<0.001
Systolic inter-arm BP difference ≥10mmHg	81 (14.9)	40 (37.4)	<0.001
Systolic inter-arm BP difference ≥ 15mmHg	10 (1.8)	6 (5.6)	0.007

Table 3. Univariable associations of risk markers with postural hypotension in derivation cohort

For peer review only - http://bmjopen.bmj.com/site/about/guidelines.xhtml

Variable	Odds Ratio	95% Confidence Interval
Model 1		
Parkinson's disease	4.7	1.2 to 19.2
Previous stroke	2.2	1.1 to 4.5
Taking digoxin	2.2	1.0 to 4.7
Previous cardiac disease	1.9	1.0 to 3.6
Hypertension	1.7	1.1 to 2.6
Systolic inter-arm difference (continuous per mmHg)	1.1	1.1 to 1.2
Model 2		
Parkinson's disease	5.0	1.2 to 19.9
Previous stroke	2.2	1.1 to 4.4
Taking digoxin	2.4	1.1 to 5.1
Previous cardiac disease	1.9	1.0 to 2.6
Hypertension	1.7	1.1 to 5.1
Systolic inter-arm difference ≥10mmHg	3.3	2.0 to 5.3
Model 3		
Parkinson's disease	5.3	1.4 to 20.4
Previous stroke	2.4	1.2 to 4.8
Taking digoxin	2.0	0.9 to 4.3
Previous cardiac disease	1.8	0.9 to 3.4
Hypertension	1.9	1.3 to 3.0

Table 4. Multivariable prediction models for postural hypotension

For peer review only

	**Including inter-arm difference**		**Excluding inter-arm difference**	
	**Weighted**	**Unweighted**	**Weighted**	**Unweighted**
Prediction of PH per unit increase of DROP score OR (95%CI)	1.9 (1.4 to 2.5)	1.8 (1.4 to 2.3)	2.4 (1.6 to 3.4)	2.0 (1.5 to 2.6)
Area under ROC curve (95%CI)	0.65 (0.59 to 0.70)	0.65 (0.60 to 0.71)	0.65 (0.59 to 0.71)	0.65 (0.59 to 0.70)
Mortality risk per unit score OR (95%CI)	1.9 (1.6 to 2.2)	1.8 (1.5 to 2.1)	2.8 (2.2 to 3.4)	2.1 (1.8 to 2.5)
Change in MMSE score over study (ANOVA)	N/A	P=0.004	N/A	P<0.001
Annual change in MMSE score (ANOVA)	N/A	P<0.001	N/A	P<0.001

Table 5. DROP score associations with postural hypotension, mortality and cognitive decline

For peer review only

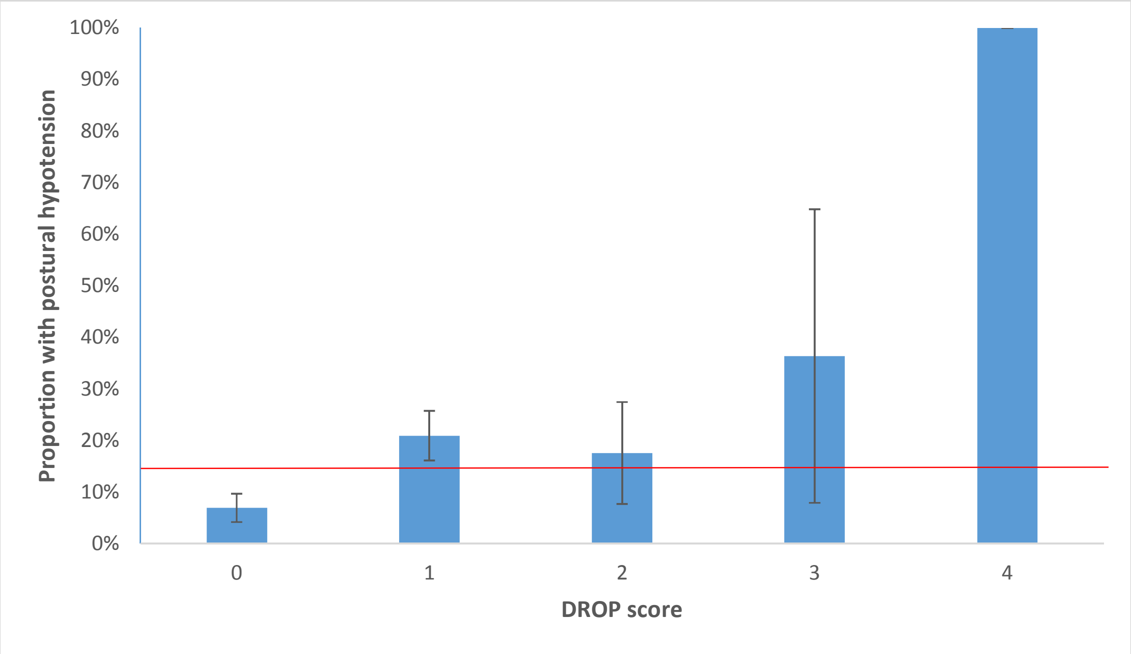

Figure 1. Prevalence of postural hypotension vs unweighted DROP Score without inter-arm difference term.

(Population prevalence indicated by horizontal line)

For peer review only - http://bmjopen.bmj.com/site/about/guidelines.xhtml

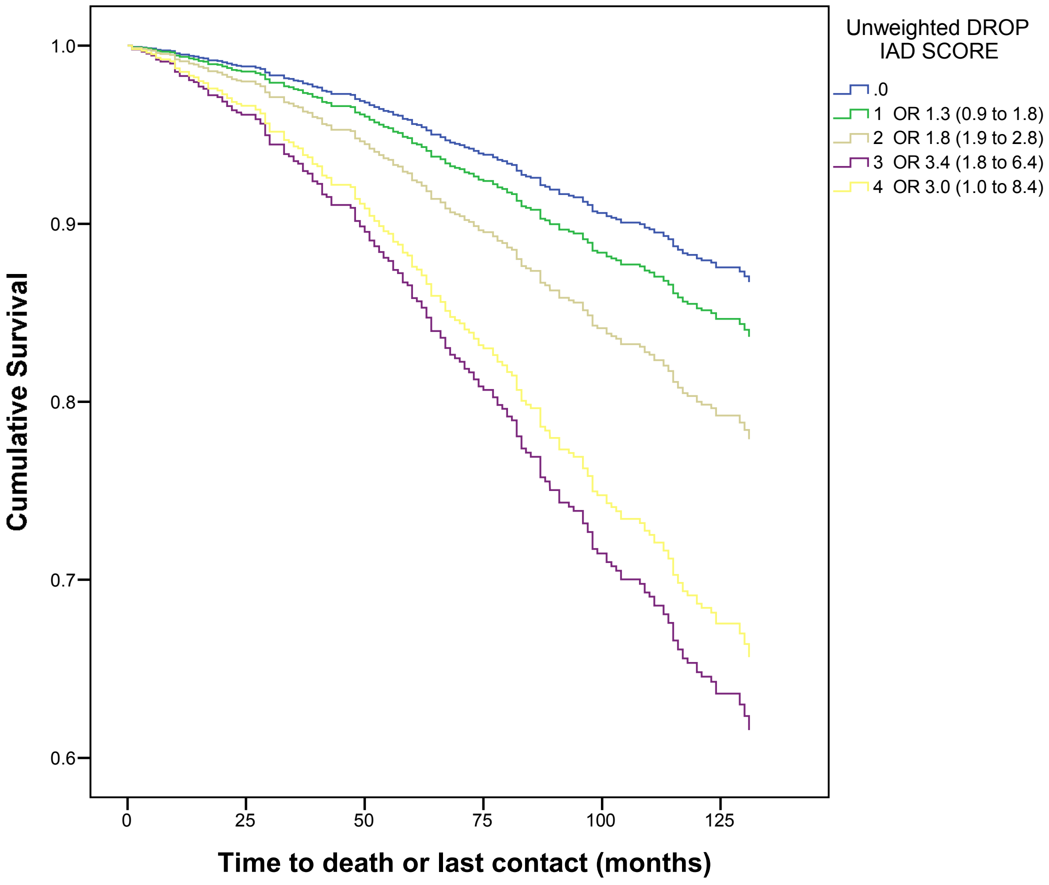

Figure 2. Kaplan Meier survival plot for DROP scores over 9 years follow up

view only

For peer review only - http://bmjopen.bmj.com/site/about/guidelines.xhtml

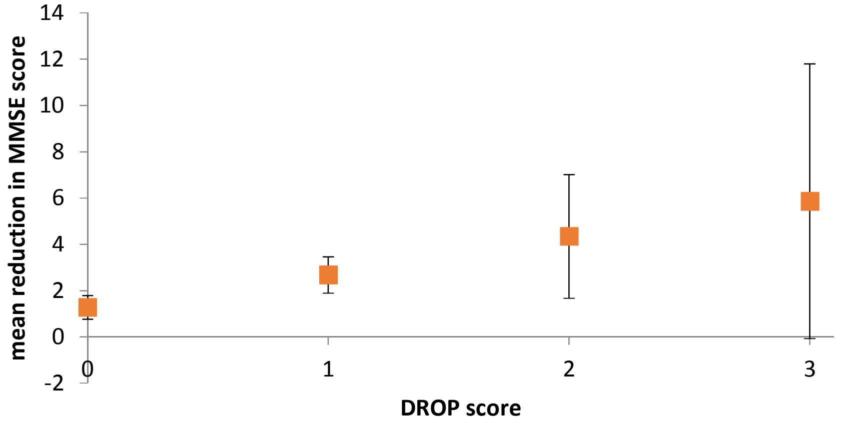

Figure 3. Mean change in Mini Mental State Examination score over nine years per DROP score

For peer review only - http://bmjopen.bmj.com/site/about/guidelines.xhtml

Appendix: Literature search for factors associated with postural hypotension

Demographics:	Increasing age[1-9]
	Female gender[10]
	Nursing home residence[11-15]
Medical History:	Hypertension[7-10 16-20] and uncontrolled hypertension[6 21 22]
	Diabetes and diabetic complications[17 23-28]
	Chronic Kidney Disease[10 29 30]
	Stroke[31-36]
	Ischaemic heart disease[36 37]
	Heart failure[38 39]
	Parkinson's disease[40-42]
	Cognitive impairment[43-50]
	Depression[51]
Medications:	Antiarrhythmic drugs[11]
	Antihypertensives[4 9-11 52-55] (negative association with ACE inhibitors)[10]
	Psychotropic agents (antipsychotics, sedatives, antidepressants)[23 53 56]
	Anticholinesterase inhibitors[50]
Biochemical:	Vitamin D deficiency (conflicting evidence)[1 23 57 58]
Frailty:[59 60]	Falls[61]
	Get up and go test[11]
	Reduced calf mass index[54 62]
	Activity of Daily Living disability score[1 11]
	Cumulative illness Rating Scale for Geriatrics score[23]
Environmental:	Seasons – prevalence higher in summer and in heatwaves[63 64]
	Time of day – higher in mornings[65-68]

For peer review only

References

1. Soysal P, Yay A, Isik AT. Does vitamin D deficiency increase orthostatic hypotension risk in the elderly patients? *Archives of Gerontology and Geriatrics* 2014;59(1):July. doi: http://dx.doi.org/10.1016/j.archger.2014.03.008
2. Moret F, Jaccard-Ruedin H, Bula C, et al. The high diagnostic yield of an outpatient geriatric clinic. *Journal of the American Geriatrics Society Conference: 2012 Annual Scientific Meeting of the American Geriatrics Society Seattle, WA United States Conference Start: 20120503 Conference End: 20120505 Conference Publication: (var pagings)* 2012;60(pp S175):April. doi: http://dx.doi.org/10.1111/j.1532-5415.2012.04000.x
3. Robertson D, Desjardin JA, Lichtenstein MJ. Distribution and observed associations of orthostatic blood pressure changes in elderly general medicine outpatients. *American Journal of the Medical Sciences* 1998;315(5):1998. doi: http://dx.doi.org/10.1097/00000441-199805000-00001
4. Poon IO, Braun U. High prevalence of orthostatic hypotension and its correlation with potentially causative medications among elderly veterans. *Journal of Clinical Pharmacy and Therapeutics* 2005;30(2):April. doi: http://dx.doi.org/10.1111/j.1365-2710.2005.00629.x
5. Mendez CA, Melgarejo JD, Lee JH, et al. Orthostatic hypotension in latino elderly: Findings from the maracaibo ageing study. *Journal of Hypertension Conference: 25th European Meeting on Hypertension and Cardiovascular Protection, ESH 2015 Milan Italy Conference Start: 20150612 Conference End: 20150615 Conference Publication: (var pagings)* 2015;33(pp e219):June. doi: http://dx.doi.org/10.1097/01.hjh.0000468017.03671.63
6. Barochiner J, Alfie J, Aparicio L, et al. Orthostatic hypotension in treated hypertensive patients. *Romanian journal of internal medicine = Revue roumaine de medecine interne* 2012;50(3):2012-2Sep.
7. Shin C, Abbott RD, Lee H, et al. Prevalence and correlates of orthostatic hypotension in middle-aged men and women in Korea: The Korean Health and Genome Study. *Journal of Human Hypertension* 2004;18(10):October. doi: http://dx.doi.org/10.1038/sj.jhh.1001732
8. Harris T, Lipsitz LA, Kleinman JC, et al. Postural change in blood pressure associated with age and systolic blood pressure. The National Health and Nutrition Examination Survey II. *Journals of Gerontology* 1991;46(5):1991.
9. Kamaruzzaman S, Watt H, Carson C, et al. The association between orthostatic hypotension and medication use in the British Women's Heart and Health Study. *Age and Ageing* 2010;39(1):afp192. doi: http://dx.doi.org/10.1093/ageing/afp192
10. Fedorowski A, Burri P, Melander O. Orthostatic hypotension in genetically related hypertensive and normotensive individuals. *Journal of Hypertension* 2009;27(5):May. doi: http://dx.doi.org/10.1097/HJH.0b013e3283279860
11. Boland E, Brackelaire G, Anani Y, et al. Postprandial hypotension among patients from an acute geriatric ward: Results from a pilot study. *European Geriatric Medicine Conference: 11th International Congress of the European Union Geriatric Medicine Society, EUGMS 2015 Oslo Norway Conference Start: 20150916 Conference End: 20150918 Conference Publication: (var pagings)* 2015;6(pp S64):September.
12. Hartog LC, Cizmar-Sweelssen M, Knipscheer A, et al. The association between orthostatic hypotension, falling and successful rehabilitation in a nursing home population. *Archives of Gerontology and Geriatrics* 2015;61(2):01. doi: http://dx.doi.org/10.1016/j.archger.2015.05.005
13. Asensio LE, Aguilera AAC, Corral MACC, et al. Prevalence of orthostatic hypotension in a series of elderly institutionalized patients. *Europace Conference: EHRA Europace 2011 Madrid Spain Conference Start: 20110626 Conference End: 20110629 Conference Publication: (var pagings)* 2011;13 doi: http://dx.doi.org/10.1093/europace/eur231
14. Matusik P, Nowak J, Tomaszewski K, et al. Hypertension among the elderly on the basis of nursing home residents population. [Polish]. *Polski Przeglad Kardiologiczny* 2010;12(3):2010.

For peer review only - http://bmjopen.bmj.com/site/about/guidelines.xhtml

15. Wu J-S, Yang Y-C, Lu F-H, et al. Population-based study on the prevalence and correlates of orthostatic hypotension/hypertension and orthostatic dizziness. *Hypertension Research* 2008;31(5):May. doi: http://dx.doi.org/10.1291/hypres.31.897
16. Saez T, Suarez C, Sierra MJ, et al. Orthostatic hypotension in the elderly and its association with the antihypertensive treatment. [Spanish]. *Medicina Clinica* 2000;114(14):15.
17. Wu JS, Yang YC, Lu FH, et al. Population-based study on the prevalence and risk factors of orthostatic hypotension in subjects with pre-diabetes and diabetes. *Diabetes Care* 2009;32(1):69-74. doi: http://dx.doi.org/10.2337/dc08-1389
18. Fan X-H, Sun K, Zhou X-L, et al. Association of orthostatic hypertension and hypotension with target organ damage in middle and old-aged hypertensive patients. [Chinese]. *National Medical Journal of China* 2011;91(4):04. doi: http://dx.doi.org/10.3760/cma.j.issn.0376-2491.2011.04.002
19. Barochiner J, Alfie J, Aparicio LS, et al. Prevalence and clinical profile of resistant hypertension among treated hypertensive subjects. *Clinical and Experimental Hypertension* 2013;35(6):2013. doi: http://dx.doi.org/10.3109/10641963.2012.739236
20. Bouhanick B, Meliani S, Doucet J, et al. Orthostatic hypotension is associated with more severe hypertension in elderly autonomous diabetic patients from the French Gerodiab study at inclusion. *Annales de Cardiologie et d Angeiologie* 2014;63(3):176-82. doi: http://dx.doi.org/10.1016/j.ancard.2014.05.013
21. Auseon A, Ooi WL, Hossain M, et al. Blood pressure behavior in the nursing home: Implications for diagnosis and treatment of hypertension. *Journal of the American Geriatrics Society* 1999;47(3):March.
22. Masuo K, Mikami H, Ogihara T, et al. Changes in frequency of orthostatic hypotension in elderly hypertensive patients under medications. *American Journal of Hypertension* 1996;9(3):March. doi: http://dx.doi.org/10.1016/0895-7061%2895%2900348-7
23. Paccalin M. Factors associated with orthostatic hypotension in hospitalized elderly patients. *European Geriatric Medicine Conference: 11th International Congress of the European Union Geriatric Medicine Society, EUGMS 2015 Oslo Norway Conference Start: 20150916 Conference End: 20150918 Conference Publication: (var pagings)* 2015;6(pp S62):September.
24. van Hateren KJJ, Kleefstra N, Blanker MH, et al. Orthostatic hypotension, diabetes, and falling in older patients: A cross-sectional study. *British Journal of General Practice* 2012;62(603):October. doi: http://dx.doi.org/10.3399/bjgp12X656838
25. Gupta A, Gilden J. Prevalence of diabetes in patients admitted to the hospital with primary diagnosis of orthostatic hypotension. *Diabetes Conference: 73rd Scientific Sessions of the American Diabetes Association Chicago, IL United States Conference Start: 20130621 Conference End: 20130625 Conference Publication: (var pagings)* 2013;62(pp A148):July. doi: http://dx.doi.org/10.2337/db13-388-679
26. Eze CO, Onwuekwe IO, Agu CE, et al. The prevalence of orthostatic hypotension in type 2 diabetes mellitus patients in a diabetic clinic in Enugu South-East Nigeria. *Nigerian journal of medicine : journal of the National Association of Resident Doctors of Nigeria* 2013;22(3):2013-2Sep.
27. Lanthier L, Touchette M, Bourget P, et al. [Evaluation of circadian variation of blood pressure by ambulatory blood pressure monitoring in an elderly diabetic population with or without orthostatic hypotension]. [French]. *Geriatrie et psychologie neuropsychiatrie du vieillissement* 2011;9(1):Mar.
28. Rota E, Quadri R, Fanti E, et al. Clinical and electrophysiological correlations in type 2 diabetes mellitus at diagnosis. *Diabetes Research and Clinical Practice* 2007;76(1):April. doi: http://dx.doi.org/10.1016/j.diabres.2006.07.027
29. Aghera D, McFadden C, Hunter K. Orthostatic hypotension in elderly individuals with chronic kidney disease(CKD). *American Journal of Kidney Diseases Conference: National Kidney Foundation 2015 Spring Clinical Meetings, NKF SCM15 Dallas, TX United States Conference*

For peer review only - http://bmjopen.bmj.com/site/about/guidelines.xhtml

Start: 20150325 Conference End: 20150329 Conference Publication: (var pagings) 2015;65(4):April.
30. Bhat S, Hegde S, Szpunar S, et al. Prevalence of orthostatic variation in blood pressure among stable outpatient chronic kidney disease population. *American Journal of Kidney Diseases Conference: National Kidney Foundation 2013 Spring Clinical Meetings Orlando, FL United States Conference Start: 20130402 Conference End: 20130406 Conference Publication: (var pagings)* 2013;61(4):April. doi: http://dx.doi.org/10.1053/j.ajkd.2013.02.049
31. Phipps MS, Schmid AA, Kapoor JR, et al. Orthostatic hypotension among outpatients with ischemic stroke. *Journal of the Neurological Sciences* 2012;314(1-2):15. doi: http://dx.doi.org/10.1016/j.jns.2011.10.031
32. Ryan DJ, Kenny RA, Christensen S, et al. Ischaemic stroke or TIA in older subjects associated with impaired dynamic blood pressure control in the absence of severe large artery stenosis. *Age and Ageing* 2015;44(4):afv011. doi: http://dx.doi.org/10.1093/ageing/afv011
33. Eguchi K, Kario K, Hoshide S, et al. Greater change of orthostatic blood pressure is related to silent cerebral infarct and cardiac overload in hypertensive subjects. *Hypertension Research* 2004;27(4):April. doi: http://dx.doi.org/10.1291/hypres.27.235
34. Kario K, Shimada K. Orthostatic blood pressure changes and silent cerebrovascular disease. *Cardiology Review* 2003;20(4):01.
35. Kwok CS, Ong ACL, Potter JF, et al. TIA, stroke and orthostatic hypotension: A disease spectrum related to ageing vasculature? *International Journal of Clinical Practice* 2014;68(6):June. doi: http://dx.doi.org/10.1111/ijcp.12373
36. Rutan GH, Hermanson B, Bild DE, et al. Orthostatic hypotension in older adults. The Cardiovascular Health Study. CHS Collaborative Research Group. *Hypertension* 1992;19(6:Pt 1):t-19.
37. Lin ZQ, Pan CM, Li WH, et al. [The correlation between postural hypotension and myocardial infarction in the elderly population]. [Chinese]. *Zhonghua nei ke za zhi [Chinese journal of internal medicine]* 2012;51(7):Jul.
38. Potocka-Plazak K, Plazak W. Orthostatic hypotension in elderly women with congestive heart failure. *Aging - Clinical and Experimental Research* 2001;13(5):2001.
39. McGann PE. Comorbidity in heart failure in the elderly. *Clinics in Geriatric Medicine* 2000;16(3):2000.
40. Rascol O, Perez-Lloret S, Damier P, et al. Falls in ambulatory non-demented patients with Parkinson's disease. *Journal of Neural Transmission* 2015;122(10):07. doi: http://dx.doi.org/10.1007/s00702-015-1396-2
41. Senard JM, Rai S, Lapeyre-Mestre M, et al. Prevalence of orthostatic hypotension in Parkinson's disease. *Journal of Neurology Neurosurgery and Psychiatry* 1997;63(5):November.
42. Palma J-A, Gomez-Esteban JC, Norcliffe-Kaufmann L, et al. Orthostatic Hypotension in Parkinson Disease: How Much You Fall or How Low You Go? *Movement Disorders* 2015;30(5):15. doi: http://dx.doi.org/10.1002/mds.26079
43. Elmstahl S, Widerstrom E. Orthostatic intolerance predicts mild cognitive impairment: incidence of mild cognitive impairment and dementia from the Swedish general population cohort Good Aging in Skane. *Clinical interventions in aging* 2014;9(pp 1993-2002):2014. doi: http://dx.doi.org/10.2147/CIA.S72316
44. Frewen J, Savva GM, Boyle G, et al. Cognitive performance in orthostatic hypotension: Findings from a nationally representative sample. *Journal of the American Geriatrics Society* 2014;62(1):January. doi: http://dx.doi.org/10.1111/jgs.12592
45. Frewen J, Finucane C, Savva GM, et al. Orthostatic hypotension is associated with lower cognitive performance in adults aged 50 plus with supine hypertension. *The journals of gerontology Series A, Biological sciences and medical sciences* 2014;69(7):Jul. doi: http://dx.doi.org/10.1093/gerona/glt171

For peer review only - http://bmjopen.bmj.com/site/about/guidelines.xhtml

46. Traykova M, Stankova T, Mehrabian S, et al. High prevalence of orthostatic hypotension in vascular and degenerative dementia. *European Journal of Medical Research Conference: 21st European Students' Conference Promising Medical Scientists Willing to Look Beyond Berlin Germany Conference Start: 20101013 Conference End: 20101017 Conference Publication: (var pagings)* 2010;15(pp 126-127):13.
47. Yap PL, Niti M, Yap KB, et al. Orthostatic hypotension, hypotension and cognitive status: early comorbid markers of primary dementia? *Dementia & Geriatric Cognitive Disorders* 2008;26(3):239-46. doi: http://dx.doi.org/10.1159/000160955
48. Sonnesyn H, Nilsen DW, Rongve A, et al. High prevalence of orthostatic hypotension in mild dementia. *Dementia and Geriatric Cognitive Disorders* 2009;28(4):November. doi: http://dx.doi.org/10.1159/000247586
49. Campbell AJ, Reinken J. Postural hypotension in old age: Prevalence, associations and prognosis. *Journal of Clinical and Experimental Gerontology* 1985;7(2):1985.
50. Isik AT, Soysal P, Mas M. Orthostatic hypotension and long-term effects of acheis on the orthostatic hypotension in elderly patients with alzheimer disease. *Alzheimer's and Dementia Conference: Alzheimer's Association International Conference 2014 Copenhagen Denmark Conference Start: 20140712 Conference End: 20140717 Conference Publication: (var pagings)* 2014;10(pp P774):July.
51. Regan CO, Kearney PM, Cronin H, et al. Oscillometric measure of blood pressure detects association between orthostatic hypotension and depression in population based study of older adults. *BMC psychiatry* 2013;13(pp 266):2013. doi: http://dx.doi.org/10.1186/1471-244X-13-266
52. Ruwald MH, Hansen ML, Lamberts M, et al. Comparison of incidence, predictors, and the impact of co-morbidity and polypharmacy on the risk of recurrent syncope in patients <85 versus >85 years of age. *American Journal of Cardiology* 2013;112(10):15. doi: http://dx.doi.org/10.1016/j.amjcard.2013.07.041
53. Pepersack T, Gilles C, Petrovic M, et al. Prevalence of orthostatic hypotension and relationship with drug use amongst older patients. *Acta clinica Belgica* 2013;68(2):2013-2Apr.
54. Kobayashi K, Yamada S. Development of a simple index, calf mass index, for screening for orthostatic hypotension in community-dwelling elderly. *Archives of Gerontology and Geriatrics* 2012;54(2):March. doi: http://dx.doi.org/10.1016/j.archger.2011.04.003
55. Di SC, Milazzo V, Bruno G, et al. Prevalence of orthostatic hypotension in a cohort of patients under antihypertensive therapy. *High Blood Pressure and Cardiovascular Prevention Conference: 2013 National Congress of the Italian Society of Hypertension, SIIA 2013 Rome Italy Conference Start: 20131003 Conference End: 20131005 Conference Publication: (var pagings)* 2013;20(3):September. doi: http://dx.doi.org/10.1007/s40292-013-0021-4
56. Rozenfeld S, Bastos Camacho LA, Peixoto VR. Medication as a risk factor for falls in older women in Brazil. *Revista Panamericana de Salud Publica/Pan American Journal of Public Health* 2003;13(6):01.
57. Soysal P, Yay A, Isik AT. Does 25-hydroxyvitamin D deficiency increase orthostatic hypotension risk in the elderly patients? *European Geriatric Medicine Conference: 10th International Congress of the European Union Geriatric Medicine Society - Geriatric Medicine Crossing Borders, EUGMS 2014 Rotterdam Netherlands Conference Start: 20140917 Conference End: 20140919 Conference* 2014;5(pp S120):September.
58. Veronese N, Bolzetta F, De RM, et al. Serum 25-hydroxyvitamin D and orthostatic hypotension in old people: The Pro.V.A. study. *Hypertension* 2014;64(3):September. doi: http://dx.doi.org/10.1161/HYPERTENSIONAHA.114.03143
59. O'Connell MDL, Savva GM, Fan CW, et al. Orthostatic hypotension, orthostatic intolerance and frailty: The Irish Longitudinal Study on Aging-TILDA. *Archives of Gerontology and Geriatrics* 2015;60(3):01. doi: http://dx.doi.org/10.1016/j.archger.2015.01.008

For peer review only - http://bmjopen.bmj.com/site/about/guidelines.xhtml

60. Rockwood MR, Howlett SE, Rockwood K. Orthostatic hypotension (OH) and mortality in relation to age, blood pressure and frailty. *Archives of Gerontology & Geriatrics* 2012;54(3):e255-e60. doi: http://dx.doi.org/10.1016/j.archger.2011.12.009
61. Lagro J, Laurenssen NCW, Schalk BWM, et al. Diastolic blood pressure drop after standing as a clinical sign for increased mortality in older falls clinic patients. *Journal of Hypertension* 2012;30(6):June. doi: http://dx.doi.org/10.1097/HJH.0b013e328352b9fd
62. Madhavan G, Goddard AA, McLeod KJ. Prevalence and Etiology of Delayed Orthostatic Hypotension in Adult Women. *Archives of Physical Medicine and Rehabilitation* 2008;89(9):September. doi: http://dx.doi.org/10.1016/j.apmr.2008.02.021
63. Weiss A, Beloosesky Y, Grinblat J, et al. Seasonal changes in orthostatic hypotension among elderly admitted patients. *Aging Clinical and Experimental Research* 2006;18(1):February.
64. Pathak A, Lapeyre-Mestre M, Montastruc J-L, et al. Heat-related morbidity in patients with orthostatic hypotension and primary autonomic failure. *Movement Disorders* 2005;20(9):September. doi: http://dx.doi.org/10.1002/mds.20571
65. Ooi WL, Barrett S, Hossain M, et al. Patterns of orthostatic blood pressure change and their clinical correlates in a frail, elderly population. *Journal of the American Medical Association* 1997;277(16):23.
66. Ward C, Kenny RA. Reproducibility of orthostatic hypotension in symptomatic elderly. *American Journal of Medicine* 1996;100(4):April. doi: http://dx.doi.org/10.1016/S0002-9343%2897%2989517-4
67. Weiss A, Grossman E, Beloosesky Y, et al. Orthostatic hypotension in acute geriatric ward: Is it a consistent finding? *Archives of Internal Medicine* 2002;162(20):15. doi: http://dx.doi.org/10.1001/archinte.162.20.2369
68. Youde JH, Manktelow B, Ward-Close S, et al. Measuring postural changes in blood pressure in the healthy elderly. *Blood Pressure Monitoring* 1999;4(1):1999.

For peer review only

For peer review only - http://bmjopen.bmj.com/site/about/guidelines.xhtml

Medline and Embase Search Strategy

Date of search 20th October 2015

	**Searches**	**Results**
1	postural hypotension.mp. [mp=ti, ab, hw, tn, ot, dm, mf, dv, kw, nm, kf, px, rx, an, ui]	3109
2	orthostatic hypotension.mp. [mp=ti, ab, hw, tn, ot, dm, mf, dv, kw, nm, kf, px, rx, an, ui]	22694
3	1 or 2	21615
4	prevalence.mp. [mp=ti, ab, hw, tn, ot, dm, mf, dv, kw, nm, kf, px, rx, an, ui]	1202953
5	3 and 4	1678
6	limit 5 to humans	1565
7	limit 6 to aged <65+ years> [Limit not valid in Ovid MEDLINE(R),Ovid MEDLINE(R) In-Process; records were retained]	661
8	remove duplicates from 7	470

For peer review only

TRIPOD Checklist: Prediction Model Development and Validation

Section/Topic	Item		Checklist Item	Page
**Title and abstract**				
Title	1	D;V	Identify the study as developing and/or validating a multivariable prediction model, the target population, and the outcome to be predicted.	1
Abstract	2	D;V	Provide a summary of objectives, study design, setting, participants, sample size, predictors, outcome, statistical analysis, results, and conclusions.	3
**Introduction**				
Background and objectives	3a	D;V	Explain the medical context (including whether diagnostic or prognostic) and rationale for developing or validating the multivariable prediction model, including references to existing models.	4
	3b	D;V	Specify the objectives, including whether the study describes the development or validation of the model or both.	4
**Methods**				
Source of data	4a	D;V	Describe the study design or source of data (e.g., randomized trial, cohort, or registry data), separately for the development and validation data sets, if applicable.	4
	4b	D;V	Specify the key study dates, including start of accrual; end of accrual; and, if applicable, end of follow-up.	4
Participants	5a	D;V	Specify key elements of the study setting (e.g., primary care, secondary care, general population) including number and location of centres.	4
	5b	D;V	Describe eligibility criteria for participants.	4
	5c	D;V	Give details of treatments received, if relevant.	N/A
Outcome	6a	D;V	Clearly define the outcome that is predicted by the prediction model, including how and when assessed.	5
	6b	D;V	Report any actions to blind assessment of the outcome to be predicted.	5
Predictors	7a	D;V	Clearly define all predictors used in developing or validating the multivariable prediction model, including how and when they were measured.	5 & table 1
	7b	D;V	Report any actions to blind assessment of predictors for the outcome and other predictors.	5
Sample size	8	D;V	Explain how the study size was arrived at.	6
Missing data	9	D;V	Describe how missing data were handled (e.g., complete-case analysis, single imputation, multiple imputation) with details of any imputation method.	N/A
Statistical analysis methods	10a	D	Describe how predictors were handled in the analyses.	5
	10b	D	Specify type of model, all model-building procedures (including any predictor selection), and method for internal validation.	5
	10c	V	For validation, describe how the predictions were calculated.	5
	10d	D;V	Specify all measures used to assess model performance and, if relevant, to compare multiple models.	5
	10e	V	Describe any model updating (e.g., recalibration) arising from the validation, if done.	N/A
Risk groups	11	D;V	Provide details on how risk groups were created, if done.	N/A
Development vs. validation	12	V	For validation, identify any differences from the development data in setting, eligibility criteria, outcome, and predictors.	None table 2
**Results**				
Participants	13a	D;V	Describe the flow of participants through the study, including the number of participants with and without the outcome and, if applicable, a summary of the follow-up time. A diagram may be helpful.	5
	13b	D;V	Describe the characteristics of the participants (basic demographics, clinical features, available predictors), including the number of participants with missing data for predictors and outcome.	table 2
	13c	V	For validation, show a comparison with the development data of the distribution of important variables (demographics, predictors and outcome).	table 2
Model development	14a	D	Specify the number of participants and outcome events in each analysis.	5
	14b	D	If done, report the unadjusted association between each candidate predictor and outcome.	table 3
Model specification	15a	D	Present the full prediction model to allow predictions for individuals (i.e., all regression coefficients, and model intercept or baseline survival at a given time point).	table 4
	15b	D	Explain how to the use the prediction model.	6
Model performance	16	D;V	Report performance measures (with CIs) for the prediction model.	6 table 5
Model-updating	17	V	If done, report the results from any model updating (i.e., model specification, model performance).	6
**Discussion**				
Limitations	18	D;V	Discuss any limitations of the study (such as nonrepresentative sample, few events per predictor, missing data).	6-7
Interpretation	19a	V	For validation, discuss the results with reference to performance in the development data, and any other validation data.	6-7
	19b	D;V	Give an overall interpretation of the results, considering objectives, limitations, results from similar studies, and other relevant evidence.	6-7
Implications	20	D;V	Discuss the potential clinical use of the model and implications for future research.	8
**Other information**				
Supplementary information	21	D;V	Provide information about the availability of supplementary resources, such as study protocol, Web calculator, and data sets.	N/A
Funding	22	D;V	Give the source of funding and the role of the funders for the present study.	14

TRIPOD Checklist: Prediction Model Development and Validation

*Items relevant only to the development of a prediction model are denoted by D, items relating solely to a validation of a prediction model are denoted by V, and items relating to both are denoted D;V. We recommend using the TRIPOD Checklist in conjunction with the TRIPOD Explanation and Elaboration document.

For peer review only

BMJ Open

Detecting Risks Of Postural Hypotension (DROP): derivation and validation of a prediction score for primary care

Journal:	*BMJ Open*
Manuscript ID	bmjopen-2017-020740.R1
Article Type:	Research
Date Submitted by the Author:	02-Jan-2018
Complete List of Authors:	Clark, Christopher; University of Exeter, Primary Care Research Group<br>Thomas, Daniel; University of Exeter, Primary Care Research Group<br>Warren, Fiona; University of Exeter Medical School, Institute of Health Research<br>Llewellyn, David; University of Exeter, Mental Health Research Group<br>Ferrucci, Luigi; Harbor Hospital, Baltimore, National Institute on Aging NIA-ASTRA Unit<br>Campbell, John; University of Exeter, Primary Care;
**Primary Subject Heading**:	General practice / Family practice
Secondary Subject Heading:	Cardiovascular medicine, Diagnostics, General practice / Family practice
Keywords:	Hypertension < CARDIOLOGY, PRIMARY CARE, PREVENTIVE MEDICINE

SCHOLARONE™ Manuscripts

For peer review only - http://bmjopen.bmj.com/site/about/guidelines.xhtml

Detecting Risks Of Postural Hypotension (DROP): derivation and validation of a prediction score for primary care

Clark CE, Thomas D, Warren F, Llewellyn D, Ferrucci L, Campbell JL

Christopher E Clark[1], Daniel Thomas[1], Fiona C Warren[1], David J. Llewellyn[2], Luigi Ferrucci[3], John L Campbell[1]

1. Primary Care Research Group
Institute of Health Research
University of Exeter Medical School
Smeall Building, St Luke's Campus
Magdalen Rd, Exeter, Devon, England EX1 2LU

2. Mental Health Research Group
Institute of Health Research
University of Exeter Medical School
College House, St Luke's Campus
Magdalen Rd, Exeter, Devon, England EX1 2LU

3. National Institute on Aging, Baltimore, Maryland, USA
251 Bayview Blvd. Room 04C228
Baltimore, MD 21224 - USA

Abstract: 296 words
Text: 3441 words
Tables: 5
Figures: 3
Appendices: 1

Address for correspondence: Dr C E Clark, as above, email: c.e.clark@exeter.ac.uk

For peer review only

Abstract

Objectives

Falls are a common problem in older people. Postural hypotension contributes to falls but is often asymptomatic. In the absence of symptoms, postural hypotension is only infrequently checked for in clinical practice. We undertook this study to derive, validate and explore the prospective associations of a prediction tool to identify people likely to have unrecognised postural hypotension.

Design and setting

Cross-sectional and prospective multivariable cohort analysis.

Participants

1317 participants of the InCHIANTI study, a population based cohort representative of the older Italian population.

Primary outcome measures

Predictive value of score to suggest presence of postural hypotension,

Methods

Subjects were randomised 1:1 to derivation or validation cohorts. Within the derivation cohort univariable associations for candidate predictors of postural hypotension were tested. Variables with P<0.1 entered multivariable linear regression models. Factors retaining multivariable significance were incorporated into unweighted and weighted DROP scores. These scores were tested in the validation cohort against prediction of postural hypotension, cognitive decline and mortality over nine years' follow up.

Results

Postural hypotension was present in 203 (15.4%) of participants. Factors predicting postural hypotension were: digoxin use, Parkinson's disease, hypertension, stroke or cardiovascular disease, and an inter-arm systolic blood pressure difference. Area under the curve was consistent at 0.65 for all models, with significant odds ratios (OR) of 1.8 to 2.4 per unit increase in score for predicting postural hypotension. For a DROP score ≥1, five cases need to be tested to identify one with postural hypotension.

Increasing DROP scores predicted mortality (OR 1.8 to 2.8 per unit rise) and increasing rates of decline of Mini Mental State Examination score (ANOVA p<0.001) over 9 years of follow up.

Conclusions

The DROP score provides a simple method to identify people likely to have postural hypotension, and increased risks to health, who require further evaluation.

(296 words)

For peer review only - http://bmjopen.bmj.com/site/about/guidelines.xhtml

Strengths and limitations of this study

- This study used data from a well-established cohort representative of an older population in Italy, to derive and validate a score (“DROP score”) to predict the presence of postural hypotension.

- Comprehensive recording of baseline variables at recruitment by the InCHIANTI investigators allowed a large number of previously reported risk markers for postural hypotension to be tested in the analyses.

- The study was undertaken according to TRIPOD guidelines and randomised splitting of the cohort allowed internal validation of the findings to be undertaken.

- We chose the consensus definition of postural hypotension as our outcome measure since we sought to predict this, rather than study postural symptoms, which should in any event trigger testing for postural hypotension.

- The population studied did not include residential or nursing home residents; refinement of the scoring system within larger cohorts more representative of primary care populations is required to confirm the potential of the DROP score in practice.

For peer review only

For peer review only - http://bmjopen.bmj.com/site/about/guidelines.xhtml

Introduction

Falls are a major cause of morbidity and mortality in older people; 35% of people older than 65 and 50% of people older than 80 fall at least once a year.[1 2] Falls are the leading cause of disability and the leading cause of death from injury among people over 75 in the UK, and cost the NHS around £2.3 billion per year.[3] Postural or orthostatic hypotension is a major risk factor for falls,[4 5] and is independently associated with increased mortality rates.[6-8] Postural hypotension has also been associated with dementia and cognitive impairment, and may have more subtle adverse effects on wellbeing and cognition.[9]

Postural hypotension is commonly defined as a fall of either ≥20mmHg in systolic blood pressure or ≥10mmHg in diastolic blood pressure, from sitting or lying, within three minutes of standing up.[10] Reported prevalences of postural hypotension vary widely, and are sensitive to both care setting, occurring in over half of patients admitted to care of the elderly,[11-13] and to the presence of co-morbidity. General adult population prevalence appears to be around 7%,[14 15] rising to 11 to 15% in persons 65 years old and older,[16-18] and 19% in those aged over 80 or older.[15] Prevalence is reported to be higher in the presence of hypertension,[19-23] stroke,[24 25] myocardial infarction,[25 26] and diabetes.[22 27]

Guidelines vary in recommendations for the detection of postural hypotension. The National Institute for Health and Care Excellence (NICE) recommends testing in the presence of symptoms whist the European Society for Hypertension also recommends testing in the elderly and in the presence of diabetes.[1 28] Unfortunately most individuals with postural hypotension are asymptomatic,[7] and we have found that, in practice, postural hypotension is seldom looked for in patients who do not report postural symptoms.[29] Anecdotally, testing is not undertaken due to time constraints; screening for postural hypotension is not supported in the literature, being regarded as lacking an evidence base, and primary care workloads are rising.[30 31] Risks of hospitalisation, nursing home admission or mortality can already be predicted by the electronic frailty index (eFI), a score derived from existing information in primary care computer records, and incorporated into many general practice computing systems. However the association of eFI with, and its ability to predict, postural hypotension (which itself is poorly tested for and recorded in primary care) is unclear,[32] and comparable frailty indices have not been found to be predictive of postural hypotension.[33] To address this gap in care we hypothesised that a simple prediction score, based on easily recognised risk markers, might help clinicians identify those most likely to have postural hypotension thereby allowing a targeted implementation of sitting and standing blood pressure measurement in the absence of symptoms. We therefore undertook the current analysis, in a well-documented cohort known to be representative of an older population living in the community. Aims were to explore the feasibility of deriving and internally validating a prediction score, to assess its value and its prospective associations.

Methods

The study was conducted and reported in accordance with the TRIPOD statement.[34] We studied participants from the InCHIANTI study; a cohort study designed to explore declining mobility in later life. The Italian National Research Council on Aging ethical committee approved the InCHIANTI study protocol, and the current analysis proposals were approved by the investigating committee of the InCHIANTI study.

The InCHIANTI study methods have been described in detail elsewhere.[35] In brief, 1270 participants aged 65 years or more were randomly selected from the population registries of two villages: Greve

For peer review only - http://bmjopen.bmj.com/site/about/guidelines.xhtml

in Chianti, and Antella in Bagno a Ripoli. Additional people were randomly selected from these sites to complete recruitment of at least 30 men and 30 women for each age decile from age 20 to 29 upwards. Extensive baseline interviews and examinations were conducted at recruitment, between September 1998 and March 2000, and follow up data were obtained after three, six and nine years. Blood pressure was initially measured supine, sequentially in both arms, to identify the higher reading arm, then a further two measurements were made on the higher reading arm. Subjects then stood and blood pressure was measured once after 1 minute and once more after 3 minutes standing. All measurements were obtained by research assistants using a standard mercury sphygmomanometer. Written informed consent was obtained from all participants at recruitment to the InCHIANTI study.

Baseline blood pressure was calculated as the mean of the second and third supine blood pressure readings.[36] Postural changes in blood pressure from lying to standing were calculated by subtraction of this mean from the standing blood pressure. Postural hypotension was considered to exist where there was as a reduction in blood pressure on standing of ≥20mmHg systolic or ≥10mmHg diastolic after 1 or after 3 minutes.[10] Hypertension was defined as use of antihypertensive drugs and/or a documented history of hypertension at recruitment.

For this analysis, participants were randomly allocated in a 1:1 ratio using a split-sample method,[37] stratified for gender and study site, to either a *derivation* or a *validation* group by a statistician (FW) blinded to postural hypotension status and medical history. A literature review was undertaken to identify potential risk markers for consideration in the analyses (appendix). These were mapped to variables available in the InCHIANTI dataset (table 1), which were then tested in the derivation cohort for univariable associations with postural hypotension, using t-tests or χ^2 tests as appropriate to the data. Variables signalling potential univariable associations (defined as p<0.1) were included in multivariable model analyses using an automated backward stepwise regression method.[38] We also included age (explored both continuously and as a dichotomous variable with cut-offs of 60, 65 and 70 years) and gender in all multivariable models. Prospective associations of postural hypotension with survival up to 9 years of follow-up were tested using Kaplan-Meier plots and Cox proportional hazard ratios. Cognitive decline was defined as a reduction in Mini Mental State Examination score (MMSE score) of 5 points or more from baseline, and rate of cognitive decline was defined as change in MMSE scores averaged per year of follow up.

Risk markers that retained significance in the multivariable models were used to derive both weighted and unweighted scores (DROP scores); weighted scores were derived by the addition of the multivariable Log (n) odds ratio (OR) for each marker present, whereas the unweighted model allocated one point for each risk marker present. Scores were tested in the validation cohort for ability to predict postural hypotension using ROC analysis, to predict future mortality using Cox proportional hazard ratios, and cognitive decline over nine years using ANOVA. All analyses were undertaken using IBM SPSS Statistics v24.0.0.2.

Results

Data for standing blood pressure existed for 1317 of the 1453 participants (91%) and they formed the cohort for this study. The derivation cohort (n=649) and validation cohort (n=668) were well matched for all important characteristics and putative risk markers (table 2); overall postural

For peer review only - http://bmjopen.bmj.com/site/about/guidelines.xhtml

hypotension was present for 203 (15.4%) of participants at recruitment. Mean age of participants was 68.3 (standard deviation 15.5).

For the derivation cohort postural hypotension was associated, over 9 years of follow-up, with increased all-cause mortality (Hazard Ratio (HR) 1.9; 95% confidence interval (95%CI) 1.4 to 2.7), cardiovascular mortality (HR 2.1; 95%CI 1.2 to 3.4), and non-cardiovascular mortality (HR 2.0; 95%CI 1.3 to 3.0). Results of univariable testing are summarised in table 3. Using a cut off value of $p<0.1$ the following candidate predictors were entered into multivariable models: age (continuous, or dichotomous for age 60 or 70 cut offs), MMSE score, angiotensin 2 antagonist, diuretic and digoxin use, presence of hypertension, any cardiovascular disease (composite of history of myocardial infarction, angina pectoris or congestive heart failure), stroke, Parkinson's disease, hospital admission within the last year, WHO disability level, any disability in activities of daily living, systolic inter-arm difference (continuous or using ≥10mmHg cut off).

Terms for systolic and diastolic blood pressure were entered into the multivariable model in a sensitivity analysis. Apart from finding that systolic blood pressure replaced the term for presence of hypertension, model outputs were unchanged. Therefore we adopted the latter for consistency with our aim to derive a pragmatic score.

Backward stepwise regression analysis produced consistent findings with any permutation of discrete and continuous variables for age (which was not retained in any model) or for inter-arm difference (model 1 and model 2; table 4). Consequently, a dichotomous cut off for inter-arm difference of ≥10mmHg was selected for simplicity, and retained with five other factors (use of digoxin, Parkinson's disease, previous stroke, previous cardiac disease and diagnosis of hypertension) to derive weighted (using log OR) and unweighted (score 1 for each factor present; possible range 0 to 6) *DROP* scores. The scores were tested in the validation cohort. Since inter-arm difference is not routinely measured a third model excluding inter-arm difference (model 3, table 4) was also used to derive DROP scores without this term (possible range 0 to 5).

All versions of the DROP score were found to predict postural hypotension in the validation cohort with similar areas under the curve of 0.65 but a trend to higher odds of postural hypotension with the exclusion of inter-arm difference from the model (Figure 1, table 5). Sensitivities and specificities of the unweighted DROP score without the inter-arm difference term were 76%, 16%, 5% and 53%, 91%, 99% respectively for cut-offs of ≥1, ≥2 and ≥3, although only 15 participants attained a DROP score of 3 and only one a score of 4. This equated to a number needed to test in order to detect one case of postural hypotension of 5, 5 and 2 for DROP scores of 1, 2 and 3 respectively. For the weighted DROP score without inter-arm difference a cut off value of 0.6 or more had a sensitivity of 74% and specificity of 55% for detection of postural hypotension. A similar pattern was seen for the DROP models including inter-arm difference; for an unweighted DROP score of 1 or more sensitivity and specificity for postural hypotension were 81% and 46% respectively predicting detection of one case of postural hypotension for every five tested. For the weighted score, a cut off value of 0.26 had a sensitivity of 81% and a specificity of 46% for detection of postural hypotension.

DROP scores were predictive of mortality over nine years of follow-up, with increasing ORs according to DROP score with adjustment for age (Figure 2). Data on MMSE were available for 529/668 (79%) of the validation cohort; classification by unweighted DROP scores was also predictive of decline in MMSE after nine years (Figure 3).

For peer review only - http://bmjopen.bmj.com/site/about/guidelines.xhtml

Discussion

Main findings

This analysis has confirmed that it is feasible, in a community living cohort of predominantly older people, to derive a score based on easily recognised risk markers that can help to identify older persons that are likely to have postural hypotension and require further clinical evaluation. The score, consisting of six risk markers: use of digoxin, presence of Parkinson's disease, hypertension, cardiovascular disease, stroke, and a difference in systolic blood pressure between arms ≥10mmHg, performs similarly with or without weighting, therefore a simple additive score is preferred. Performance is also similar when the inter-arm term is omitted, further simplifying its application.

In this population, postural hypotension is associated with a doubling of risk of death over nine years of follow-up. The DROP score also predicts increasing future mortality from any cause and is associated with greater decline in Mini Mental State Examination scores.

Strengths and weaknesses

The cohort was chosen as representative of a free-living elderly population and the 15.4% prevalence of postural hypotension is consistent with figures ranging from 11 to 15% in other general elderly (over 65) populations.[16-18] Comprehensive recording of baseline variables allowed a large number of previously reported risk factors for postural hypotension to be tested. Since this was undertaken as a feasibility study no formal sample size calculation was undertaken, however there were sufficient events to support the multivariable analyses performed.[38] Although the relatively low numbers attaining DROP scores higher than 2 did lead to imprecision around the predictive values of those higher levels of scores. Re-analysis and external validation in a larger sized cohort could overcome this limitation. Blood pressures were measured supine and standing for this study whereas in practice sitting and standing measurements are commonly recommended.[36] These are less sensitive but more practical in primary care,[39] however a score derived in supine to standing cases of postural hypotension cannot be assumed to perform similarly in the sitting to standing setting. Therefore, we regard this analysis as a feasibility study that supports the concept of a simple pragmatic prediction score to aid daily practice, in need of refinement through larger scale analyses, and exploration in cohorts with sit to stand measurements.

Relevance to literature

Postural hypotension has previously been reported as a significant independent predictor of four year all-cause mortality in the Honolulu Heart programme.[6] It also predicted mortality in the Malmo Heart study,[8] but not in the Helsinki ageing study.[40] Frailty was associated with a higher prevalence of postural hypotension in the TILDA study, and adjustment for frailty may influence associations with mortality.[41 42] However no measures of frailty remained predictive of postural hypotension on inclusion in the current multivariable analyses, and a frailty index predicted postural *symptoms* but not postural hypotension within TILDA.[33]

Prevalence of postural hypotension rises with age.[15] Although those with postural hypotension in this study were on average five years older age was not a significant independent predictor of postural hypotension in our models. This may have been in part due to the skewed nature of the age profile in InCHIANTI, although sensitivity analyses excluding those under 65 made no difference (not reported). Prevalence of postural hypotension is elevated in association with a history of stroke or TIA,[43-45] cardiovascular disease,[24-26] diabetes,[22 27] or hypertension, which itself affects over 60% of the over 65 age group.[46] Thus the significant factors in our models were all age related conditions which

For peer review only - http://bmjopen.bmj.com/site/about/guidelines.xhtml

seems the likely explanation for loss of age itself as an independent predictor due to collinearity. Parkinson's disease was the strongest predictor of postural hypotension in our analyses although, affecting only 1.1% of participants, it was also the least common factor. Postural hypotension has previously been reported to have prevalence approaching 50% in some groups of Parkinson's sufferers,[47 48] although only a third of those with postural hypotension report symptoms.[49]

The association of postural hypotension with presence of an inter-arm difference is, to our knowledge, a novel finding. We have previously associated inter-arm difference with white coat effects, which can confound detection of postural hypotension.[50 51] Arterial stiffness is a postulated cause of inter-arm difference,[52] and is also associated with postural hypotension;[53 54] thus inter-arm difference as a proxy measure of arterial stiffness might account for the observed association. Hypotension on ambulatory monitoring and elevated pulse-wave velocity are both associated with cognitive decline, lending further support to the association of inter-arm difference, arterial stiffness, and postural hypotension.[55]

Although postural hypotension is associated with diabetes, and with other complications such as neuropathy, retinopathy and proteinuria,[56] there was no univariable association in this study. Prevalence of postural hypotension in diabetes is associated with complications and duration of disease;[57 58] in this cohort diabetes was present in only 6% of participants, whereas recent data suggest that 25% of adults over the age of 65 in the US have it.[59] Therefore a validation of our models in other larger representative populations is needed.

Postural hypotension has been associated with mild cognitive impariement.[60 61] and reduced cognitive performance.[62] Postural hypotension did not predict cognitive decline in a 2 year prospective study of older Finns,[63] but is predictive over longer follow up.[64] In the current analysis postural hypotension per se was not predictive of cognitive decline over nine years of follow up but the DROP score was. This seems plausible given that it includes a number of risk markers known to be associated with cognitive decline.

Relevance to clinical practice

Testing sitting (or lying) and standing blood pressure takes time and training. The skills of nurses measuring postural hypotension are variable when compared with guidelines;[65] incorrect arm positioning can underestimate postural hypotension,[66] and the alerting reaction can over-estimate it.[67] Early and accurate detection of postural hypotension is a pre-requisite to intervening with medication withdrawal to reduce postural blood pressure drops and their associated risks including falls. Currently symptoms appear to be the main trigger for testing.[29] This should continue, however, a tool to identify which *asymptomatic* patients to test may help to target additional testing to those most likely to benefit. A DROP score of one or more appears to have such potential, and may support proposals that individuals at elevated risk of postural hypotension should be tested.[68]

The strongly cardiovascular composition of the DROP score means that patients will commonly be taking antihypertensive drugs. Potential adverse effects of withdrawing antihypertensive medication to ameliorate postural hypotension are unclear, and medication withdrawal may concern clinicians, carers and patients. Risk of falls rises incrementally with each added orthostatic drug.[69] Prevalence of postural hypotension in hypertension is related to use of cardiovascular drugs (antihypertensive agents, vasodilators, diuretics),[70 71] alpha blockers,[72] and the number of antihypertensive drugs used,[73 74] and is associated with resistant or uncontrolled hypertension.[75 76] Successful treatment of blood pressure in the elderly is in fact associated with lower prevalence of postural hypotension,[77 78] but withdrawal of antihypertensive therapy improves postural hypotension.[79 80]

For peer review only - http://bmjopen.bmj.com/site/about/guidelines.xhtml

We retained Parkinson's disease in our models due to the strength of the association with postural hypotension, however, on clinical grounds, testing for postural hypotension would be better regarded as integral to any review in Parkinson's disease, given the high prevalence of postural hypotension in this condition.[49]

We sought to develop a pragmatic score to support busy clinicians, faced with a rising workload and increasingly multimorbid caseload.[31] Although measurement of blood pressure in both arms has become more frequent over time it is not part of a routine review.[29 81] Therefore we derived a DROP score omitting inter-arm difference, which performed with similar sensitivity and specificity. For the same reasons, we prefer the unweighted score as a practical aide memoire to recognition of the risk of postural hypotension.

Further research

This study has examined the feasibility of identifying whom should be tested with sitting and standing blood pressure measurements to detect asymptomatic postural hypotension. It seems that a simple pragmatic scoring system can support this. We need to refine and externally validate this approach in larger samples more representative of UK primary care. Further work is needed to examine the feasibility and implications of medication review and antihypertensive withdrawal based on detection of postural hypotension in primary care.

Conclusion

We have described the derivation and validation of a score predicting the presence of postural hypotension. Initial testing suggests this approach to be feasible, and has identified the potential utility of the score in predicting mortality and cognitive decline over a nine year period of follow up. Further validation of the score in larger cohorts of individuals is warranted.

For peer review only - http://bmjopen.bmj.com/site/about/guidelines.xhtml

References

1. Falls: Assessment and prevention of falls in older people. London: National Institute for Health and Care Excellence 2013.
2. Blake AJ, Morgan K, Bendall MJ, et al. FALLS BY ELDERLY PEOPLE AT HOME: PREVALENCE AND ASSOCIATED FACTORS. *Age and Ageing* 1988;17(6):365-72. doi: 10.1093/ageing/17.6.365
3. The Importance of Vision in Preventing Falls. London: The College of Optometrists, British Geriatrics Society 2011.
4. Juraschek SP, Daya N, Appel LJ, et al. Orthostatic Hypotension in Middle-Age and Risk of Falls. *American Journal of Hypertension* 2017;30(2):188-95. doi: 10.1093/ajh/hpw108
5. McDonald C, Pearce M, Kerr SR, et al. A prospective study of the association between orthostatic hypotension and falls: Definition matters. *Age and Ageing* 2017;46(3):439-45. doi: http://dx.doi.org/10.1093/ageing/afw227
6. Masaki KH, Schatz IJ, Burchfiel CM, et al. Orthostatic hypotension predicts mortality in elderly men: The Honolulu Heart Program. *Circulation* 1998;98(21):24.
7. Benvenuto LJ, Krakoff LR. Morbidity and Mortality of Orthostatic Hypotension: Implications for Management of Cardiovascular Disease. *American Journal of Hypertension* 2011;24(2):135-44.
8. Fedorowski A, Stavenow L, Hedblad B, et al. Orthostatic hypotension predicts all-cause mortality and coronary events in middle-aged individuals (The Malmo Preventive Project). *European Heart Journal* 2010;31(1):85-91. doi: http://dx.doi.org/10.1093/eurheartj/ehp329
9. Mehrabian S, Duron E, Labouree F, et al. Relationship between orthostatic hypotension and cognitive impairment in the elderly. *J Neurol Sci* 2010;299(1-2):45-8. doi: 10.1016/j.jns.2010.08.056 [published Online First: 2010/09/22]
10. Freeman R, Wieling W, Axelrod FB, et al. Consensus statement on the definition of orthostatic hypotension, neurally mediated syncope and the postural tachycardia syndrome. *Clin Auton Res* 2011;21(2):69-72. doi: 10.1007/s10286-011-0119-5
11. Boland E, Brackelaire G, Anani Y, et al. Postprandial hypotension among patients from an acute geriatric ward: Results from a pilot study. *European Geriatric Medicine Conference: 11th International Congress of the European Union Geriatric Medicine Society, EUGMS 2015 Oslo Norway Conference Start: 20150916 Conference End: 20150918 Conference Publication: (var pagings)* 2015;6(pp S64):September.
12. Potocka-Plazak K, Plazak W. Orthostatic hypotension in elderly women with congestive heart failure. *Aging - Clinical and Experimental Research* 2001;13(5):2001.
13. McGann PE. Comorbidity in heart failure in the elderly. *Clinics in Geriatric Medicine* 2000;16(3):2000.
14. Vara-Gonzalez L, Munoz-Cacho P, De Castro SS. Postural changes in blood pressure in the general population of Cantabria (northern Spain). *Blood Pressure Monitoring* 2008;13(5):October. doi: http://dx.doi.org/10.1097/MBP.0b013e32830d4b33
15. Finucane C, O'Connell MD, Fan CW, et al. Age-related normative changes in phasic orthostatic blood pressure in a large population study: findings from The Irish Longitudinal Study on Ageing (TILDA). *Circulation* 2014;130(20):11. doi: http://dx.doi.org/10.1161/CIRCULATIONAHA.114.009831
16. Atli T, Keven K. Orthostatic hypotension in the healthy elderly. *Archives of Gerontology and Geriatrics* 2006;43(3):November/December. doi: http://dx.doi.org/10.1016/j.archger.2005.12.001
17. Mader SL, Josephson KR, Rubenstein LZ. Low prevalence of postural hypotension among community-dwelling elderly. *Journal of the American Medical Association* 1987;258(11):1987. doi: http://dx.doi.org/10.1001/jama.258.11.1511
18. Vara GL, Dominguez RR, Fernandez RM, et al. Prevalence of orthostatic hypotension in elderly hypertensive patients in primary care. [Spanish]. *Atencion primaria / Sociedad Espanola de Medicina de Familia y Comunitaria* 2001;28(3):2001-2Aug.

For peer review only - http://bmjopen.bmj.com/site/about/guidelines.xhtml

19. Shin C, Abbott RD, Lee H, et al. Prevalence and correlates of orthostatic hypotension in middle-aged men and women in Korea: The Korean Health and Genome Study. *Journal of Human Hypertension* 2004;18(10):October. doi: http://dx.doi.org/10.1038/sj.jhh.1001732
20. Saez T, Suarez C, Sierra MJ, et al. Orthostatic hypotension in the elderly and its association with the antihypertensive treatment. [Spanish]. *Medicina Clinica* 2000;114(14):15.
21. Harris T, Lipsitz LA, Kleinman JC, et al. Postural change in blood pressure associated with age and systolic blood pressure. The National Health and Nutrition Examination Survey II. *Journals of Gerontology* 1991;46(5):1991.
22. Wu JS, Yang YC, Lu FH, et al. Population-based study on the prevalence and risk factors of orthostatic hypotension in subjects with pre-diabetes and diabetes. *Diabetes Care* 2009;32(1):69-74. doi: http://dx.doi.org/10.2337/dc08-1389
23. Applegate WB, Davis BR, Black HR, et al. Prevalence of postural hypotension at baseline in the systolic hypertension in the elderly program (SHEP) cohort. *Journal of the American Geriatrics Society* 1991;39(11):1991.
24. Kwok CS, Ong ACL, Potter JF, et al. TIA, stroke and orthostatic hypotension: A disease spectrum related to ageing vasculature? *International Journal of Clinical Practice* 2014;68(6):June. doi: http://dx.doi.org/10.1111/ijcp.12373
25. Rutan GH, Hermanson B, Bild DE, et al. Orthostatic hypotension in older adults. The Cardiovascular Health Study. CHS Collaborative Research Group. *Hypertension* 1992;19(6:Pt 1):t-19.
26. Lin ZQ, Pan CM, Li WH, et al. [The correlation between postural hypotension and myocardial infarction in the elderly population]. [Chinese]. *Zhonghua nei ke za zhi [Chinese journal of internal medicine]* 2012;51(7):Jul.
27. Gupta A, Gilden J. Prevalence of diabetes in patients admitted to the hospital with primary diagnosis of orthostatic hypotension. *Diabetes Conference: 73rd Scientific Sessions of the American Diabetes Association Chicago, IL United States Conference Start: 20130621 Conference End: 20130625 Conference Publication: (var pagings)* 2013;62(pp A148):July. doi: http://dx.doi.org/10.2337/db13-388-679
28. Mancia G, Fagard R, Narkiewicz K, et al. 2013 ESH/ESC Guidelines for the management of arterial hypertension. *J Hypertens* 2013;31:1281-357.
29. Mejzner N, Clark CE, Smith LF, et al. Trends in the diagnosis and management of hypertension: repeated primary care survey in South West England. *British Journal of General Practice* 2017 doi: 10.3399/bjgp17X690461
30. Hale WA, Chambliss ML. Should primary care patients be screened for orthostatic hypotension? *The Journal of family practice* 1999;48(7):Jul.
31. Hobbs FD, Bankhead C, Mukhtar T, et al. Clinical workload in UK primary care: a retrospective analysis of 100 million consultations in England, 2007-14. *Lancet* 2016;387(10035):2323-30. doi: 10.1016/s0140-6736(16)00620-6 [published Online First: 2016/04/10]
32. Clegg A, Bates C, Young J, et al. Development and validation of an electronic frailty index using routine primary care electronic health record data. *Age and Ageing* 2016;45(3):353-60. doi: 10.1093/ageing/afw039
33. O'Connell MDL, Savva GM, Fan CW, et al. Orthostatic hypotension, orthostatic intolerance and frailty: The Irish Longitudinal Study on Aging-TILDA. *Archives of Gerontology and Geriatrics* 2015;60(3):507-13. doi: http://dx.doi.org/10.1016/j.archger.2015.01.008
34. Collins GS, Reitsma JB, Altman DG, et al. Transparent reporting of a multivariable prediction model for individual prognosis or diagnosis (TRIPOD): the TRIPOD statement. *BMJ : British Medical Journal* 2015;350 doi: 10.1136/bmj.g7594
35. Ferrucci L, Bandinelli S, Benvenuti E, et al. Subsystems contributing to the decline in ability to walk: bridging the gap between epidemiology and geriatric practice in the InCHIANTI study. *J AmGeriatrSoc* 2000;48(12):1618-25.

For peer review only - http://bmjopen.bmj.com/site/about/guidelines.xhtml

36. Daskalopoulou SS, Rabi DM, Zarnke KB, et al. The 2015 Canadian Hypertension Education Program recommendations for blood pressure measurement, diagnosis, assessment of risk, prevention, and treatment of hypertension. *Can J Cardiol* 2015;31(5):549-68. doi: 10.1016/j.cjca.2015.02.016
37. Steyerberg EW. Clinical prediction models : a practical approach to development, validation, and updating. New York ; London: Springer 2009.
38. Collett D. Modelling survival data in medical research. Boca Raton, Fla: Chapman & Hall/CRC 2003.
39. Cooke J, Carew S, O'Connor M, et al. Sitting and standing blood pressure measurements are not accurate for the diagnosis of orthostatic hypotension. *QJM: An International Journal of Medicine* 2009;102(5):335-39. doi: 10.1093/qjmed/hcp020
40. Tilvis RS, Hakala S-M, Valvanne J, et al. Postural hypotension and dizziness in a general aged population: A four- year follow-up of the Helsinki Aging Study. *Journal of the American Geriatrics Society* 1996;44(7):July.
41. Rockwood MR, Howlett SE, Rockwood K. Orthostatic hypotension (OH) and mortality in relation to age, blood pressure and frailty. *Archives of Gerontology & Geriatrics* 2012;54(3):e255-e60. doi: http://dx.doi.org/10.1016/j.archger.2011.12.009
42. O'Connell MDL, Savva GM, Fan CW, et al. Orthostatic hypotension, orthostatic intolerance and frailty: The Irish Longitudinal Study on Aging-TILDA. *Archives of Gerontology and Geriatrics* 2015;60(3):01. doi: http://dx.doi.org/10.1016/j.archger.2015.01.008
43. Phipps MS, Schmid AA, Kapoor JR, et al. Orthostatic hypotension among outpatients with ischemic stroke. *Journal of the Neurological Sciences* 2012;314(1-2):15. doi: http://dx.doi.org/10.1016/j.jns.2011.10.031
44. Eguchi K, Kario K, Hoshide S, et al. Greater change of orthostatic blood pressure is related to silent cerebral infarct and cardiac overload in hypertensive subjects. *Hypertension Research* 2004;27(4):April. doi: http://dx.doi.org/10.1291/hypres.27.235
45. Kario K, Shimada K. Orthostatic blood pressure changes and silent cerebrovascular disease. *Cardiology Review* 2003;20(4):01.
46. Falaschetti E, Mindell J, Knott C, et al. Hypertension management in England: a serial cross-sectional study from 1994 to 2011. *Lancet* 2014;383(9932):1912-19.
47. Rascol O, Perez-Lloret S, Damier P, et al. Falls in ambulatory non-demented patients with Parkinson's disease. *Journal of Neural Transmission* 2015;122(10):07. doi: http://dx.doi.org/10.1007/s00702-015-1396-2
48. Senard JM, Rai S, Lapeyre-Mestre M, et al. Prevalence of orthostatic hypotension in Parkinson's disease. *Journal of Neurology Neurosurgery and Psychiatry* 1997;63(5):November.
49. Palma J-A, Gomez-Esteban JC, Norcliffe-Kaufmann L, et al. Orthostatic Hypotension in Parkinson Disease: How Much You Fall or How Low You Go? *Movement Disorders* 2015;30(5):15. doi: http://dx.doi.org/10.1002/mds.26079
50. Schwartz CL, Clark C, Koshiaris C, et al. Interarm Difference in Systolic Blood Pressure in Different Ethnic Groups and Relationship to the "White Coat Effect": A Cross-Sectional Study. *Am J Hypertens* 2017 doi: 10.1093/ajh/hpx073
51. Kamaruzzaman S, Watt H, Carson C, et al. The association between orthostatic hypotension and medication use in the British Women's Heart and Health Study. *Age and Ageing* 2010;39(1):51-56. doi: http://dx.doi.org/10.1093/ageing/afp192
52. Clark CE. The interarm blood pressure difference: Do we know enough yet? *The Journal of Clinical Hypertension* 2017;19(5):462-65. doi: 10.1111/jch.12982
53. Liu K, Wang S, Wan S, et al. Arterial Stiffness, Central Pulsatile Hemodynamic Load, and Orthostatic Hypotension. *Journal of Clinical Hypertension* 2016;18(7):655-62. doi: http://dx.doi.org/10.1111/jch.12726

For peer review only - http://bmjopen.bmj.com/site/about/guidelines.xhtml

54. Meng Q, Wang S, Wang Y, et al. Arterial stiffness is a potential mechanism and promising indicator of orthostatic hypotension in the general population. *Vasa - European Journal of Vascular Medicine* 2014;43(6):423-32. doi: http://dx.doi.org/10.1024/0301-1526/a000389
55. Scuteri A, Tesauro M, Guglini L, et al. Aortic stiffness and hypotension episodes are associated with impaired cognitive function in older subjects with subjective complaints of memory loss. *Int J Cardiol* 2013;169(5):371-7. doi: 10.1016/j.ijcard.2013.09.009 [published Online First: 2013/10/15]
56. Eze CO, Onwuekwe IO, Agu CE, et al. The prevalence of orthostatic hypotension in type 2 diabetes mellitus patients in a diabetic clinic in Enugu South-East Nigeria. *Nigerian journal of medicine : journal of the National Association of Resident Doctors of Nigeria* 2013;22(3):2013-2Sep.
57. Lanthier L, Touchette M, Bourget P, et al. [Evaluation of circadian variation of blood pressure by ambulatory blood pressure monitoring in an elderly diabetic population with or without orthostatic hypotension]. [French]. *Geriatrie et psychologie neuropsychiatrie du vieillissement* 2011;9(1):Mar.
58. Rota E, Quadri R, Fanti E, et al. Clinical and electrophysiological correlations in type 2 diabetes mellitus at diagnosis. *Diabetes Research and Clinical Practice* 2007;76(1):April. doi: http://dx.doi.org/10.1016/j.diabres.2006.07.027
59. Kirkman MS, Briscoe VJ, Clark N, et al. Diabetes in Older Adults. *Diabetes Care* 2012;35(12):2650-64. doi: 10.2337/dc12-1801
60. Elmstahl S, Widerstrom E. Orthostatic intolerance predicts mild cognitive impairment: incidence of mild cognitive impairment and dementia from the Swedish general population cohort Good Aging in Skane. *Clinical interventions in aging* 2014;9(pp 1993-2002):2014. doi: http://dx.doi.org/10.2147/CIA.S72316
61. Frewen J, Savva GM, Boyle G, et al. Cognitive performance in orthostatic hypotension: Findings from a nationally representative sample. *Journal of the American Geriatrics Society* 2014;62(1):January. doi: http://dx.doi.org/10.1111/jgs.12592
62. Frewen J, Finucane C, Savva GM, et al. Orthostatic hypotension is associated with lower cognitive performance in adults aged 50 plus with supine hypertension. *The journals of gerontology Series A, Biological sciences and medical sciences* 2014;69(7):Jul. doi: http://dx.doi.org/10.1093/gerona/glt171
63. Viramo P, Luukinen H, Koski K, et al. Orthostatic hypotension and cognitive decline in older people. *Journal of the American Geriatrics Society* 1999;47(5):May.
64. Wolters FJ, Mattace-Raso FUS, Koudstaal PJ, et al. Orthostatic Hypotension and the Long-Term Risk of Dementia: A Population-Based Study. *PLoS Medicine* 2016;13 (10) (no pagination)(e1002143) doi: http://dx.doi.org/10.1371/journal.pmed.1002143
65. Vloet LCM, Smits R, Frederiks CMA, et al. Evaluation of skills and knowledge on orthostatic blood pressure measurements in elderly patients. *Age and Ageing* 2002;31(3):2002. doi: http://dx.doi.org/10.1093/ageing/31.3.211
66. Netea RT, Elving LD, Lutterman JA, et al. Body position and blood pressure measurement in patients with diabetes mellitus. *Journal of Internal Medicine* 2002;251(5):2002. doi: http://dx.doi.org/10.1046/j.1365-2796.2002.00958.x
67. Mo R, Omvik P, Lund-Johansen P. The Bergen Blood Pressure Study. Estimated prevalence of postural hypotension is influenced by the alerting reaction to blood pressure measurement. *Journal of Human Hypertension* 1994;8(3):1994.
68. Hale WA, Chambliss ML. Should primary care patients be screened for orthostatic hypotension? *The Journal of family practice* 1999;48(7):547-52.
69. Ruwald MH, Hansen ML, Lamberts M, et al. Comparison of incidence, predictors, and the impact of co-morbidity and polypharmacy on the risk of recurrent syncope in patients <85 versus >85 years of age. *American Journal of Cardiology* 2013;112(10):15. doi: http://dx.doi.org/10.1016/j.amjcard.2013.07.041

For peer review only - http://bmjopen.bmj.com/site/about/guidelines.xhtml

70. Pepersack T, Gilles C, Petrovic M, et al. Prevalence of orthostatic hypotension and relationship with drug use amongst older patients. *Acta clinica Belgica* 2013;68(2):2013-2Apr.
71. Poon IO, Braun U. High prevalence of orthostatic hypotension and its correlation with potentially causative medications among elderly veterans. *Journal of Clinical Pharmacy and Therapeutics* 2005;30(2):April. doi: http://dx.doi.org/10.1111/j.1365-2710.2005.00629.x
72. Kobayashi K, Yamada S. Development of a simple index, calf mass index, for screening for orthostatic hypotension in community-dwelling elderly. *Archives of Gerontology and Geriatrics* 2012;54(2):March. doi: http://dx.doi.org/10.1016/j.archger.2011.04.003
73. Di SC, Milazzo V, Bruno G, et al. Prevalence of orthostatic hypotension in a cohort of patients under antihypertensive therapy. *High Blood Pressure and Cardiovascular Prevention Conference: 2013 National Congress of the Italian Society of Hypertension, SIIA 2013 Rome Italy Conference Start: 20131003 Conference End: 20131005 Conference Publication: (var pagings)* 2013;20(3):September. doi: http://dx.doi.org/10.1007/s40292-013-0021-4
74. Kamaruzzaman S, Watt H, Carson C, et al. The association between orthostatic hypotension and medication use in the British Women's Heart and Health Study. *Age and Ageing* 2010;39(1):afp192.
75. Barochiner J, Alfie J, Aparicio LS, et al. Prevalence and clinical profile of resistant hypertension among treated hypertensive subjects. *Clinical and Experimental Hypertension* 2013;35(6):2013. doi: http://dx.doi.org/10.3109/10641963.2012.739236
76. Bouhanick B, Meliani S, Doucet J, et al. Orthostatic hypotension is associated with more severe hypertension in elderly autonomous diabetic patients from the French Gerodiab study at inclusion. *Annales de Cardiologie et d Angeiologie* 2014;63(3):176-82. doi: http://dx.doi.org/10.1016/j.ancard.2014.05.013
77. Auseon A, Ooi WL, Hossain M, et al. Blood pressure behavior in the nursing home: Implications for diagnosis and treatment of hypertension. *Journal of the American Geriatrics Society* 1999;47(3):March.
78. Masuo K, Mikami H, Ogihara T, et al. Changes in frequency of orthostatic hypotension in elderly hypertensive patients under medications. *American Journal of Hypertension* 1996;9(3):March. doi: http://dx.doi.org/10.1016/0895-7061%2895%2900348-7
79. Fotherby MD, Potter JF. Orthostatic hypotension and anti-hypertensive therapy in the elderly. *Postgraduate Medical Journal* 1994;70(830):1994.
80. Fotherby MD, Robinson TG, Potter JF. Clinic and 24 h blood pressure in elderly treated hypertensives with postural hypotension. *Journal of Human Hypertension* 1994;8(9):1994.
81. Parker E, Glasziou P. Use of evidence in hypertension guidelines: should we measure in both arms? *British Journal of General Practice* 2009;59:e87-e92. doi: 10.3399/bjgp09X395012

For peer review only

For peer review only - http://bmjopen.bmj.com/site/about/guidelines.xhtml

Authors' contributions

CEC conceived and undertook this analysis. DT contributed to the analysis. FW contributed to the analysis and offered statistical advice and support. DL offered advice on analysis and interpretation of cognitive impairment indices. LF supported the study on behalf of the InCHIANTI investigators. JLC supervised study conduct. CEC drafted the manuscript, all authors revised and edited the manuscript and all authors have read, reviewed, and approved the final manuscript.

Funding statement

CEC is supported by an NIHR Clinical Lectureship award. The views expressed are those of the authors and not necessarily those of the NIHR, the NHS or the Department of Health. This work was supported in part by the Intramural Research Program of the National Institute on Aging, National Institute of Health, Baltimore MD21224, USA.

Competing interests statement

All authors assert that they have no competing interests to declare

Previous dissemination

Interim reports on this work have been presented at annual scientific meetings of the European Society for Hypertension, Paris 2016 (Clark C, Thomas D, Warren F et al. Predicting postural hypotension, falls, and cognitive impairment: the InCHIANTI study. *J Hypertens* 2016; 34, e-Supplement 2: e32, September 2016) and the British and Irish Hypertension Society, Dublin, 2016 (Clark C, Thomas D, Mejzner N, et al. Can we predict who should be tested for postural hypotension? Derivation and validation of a prediction tool. *Journal of Human Hypertension* 2016;30 doi: doi:10.1038/jhh.2016.60)

Data sharing statement

The InCHIANTI datasets are available on application with a research proposal to the InCHIANTI investigators at http://inchiantistudy.net/wp/

For peer review only

Legends for tables and figures

Table 1. Risk markers included in univariable analysis

Table 2. Baseline characteristics of derivation and validation cohorts

Table 3. Univariable associations of risk markers with postural hypotension in derivation cohort

Table 4. Multivariable prediction models for postural hypotension

Table 5. DROP score associations with postural hypotension, mortality and cognitive decline

Figure 1. Prevalence of postural hypotension vs unweighted DROP Score without inter-arm difference term (Population prevalence indicated by horizontal line)

Figure 2. Kaplan Meier survival plot for DROP scores over 9 years follow up

Figure 3. Mean change in Mini Mental State Examination score over nine years per DROP score

For peer review only

Group	Risk marker included in analysis
Demographics	age<br>gender
Medical History	hypertension<br>heart failure<br>myocardial infarction<br>angina<br>stroke<br>diabetes<br>Parkinson's disease<br>cancer<br>dementia
Examination	Mini Mental State Examination
Medications	antihypertensives<br>antiarrhythmics<br>antidepressants<br>antipsychotics<br>anxiolytics<br>anticholinesterase inhibitors
Frailty	hospital admission, fall, or weight loss in last 12 months<br>WHO physical disability level<br>ADL disability score

Table 1. Risk markers included in univariable analysis

For peer review only

	Derivation Cohort	Validation Cohort	p
**N**	**649**	**668**	
	**Mean (SD) or N/%**	**Mean (SD) or N/%**	**t/ χ^2**
**age**	68.5 (15.7)	68.2 (15.3)	0.77
**BMI**	27.2 (4.3)	27.1 (4.0)	0.59
**Supine SBP (higher arm)#**	145.9 (21.3)	146.3 (21.6)	0.76
**Supine DBP (higher arm)#**	82.9 (8.8)	83.1 (9.5)	0.59
**Standing SBP 1 min**	140.4 (21.0)	141.2 (21.3)	0.51
**Standing DBP 1 min**	83.0 (8.9)	83.6 (9.4)	0.25
**Standing SBP 3 min**	141.4 (20.9)	141.9 (20.9)	0.66
**Standing DBP 3 min**	82.7 (9.0)	83.0 (9.4)	0.60
**Female**	368 (56.7)	358 (53.6)	0.27
**Site (Greve vs Bagno a Ripoli)**	320 vs 329	327 vs 341	0.91
**Deceased @ 9 years**	199 (30.7)	203 (30.4)	0.95
**Systolic drop ≥20mmHg 1min**	56 (8.6)	45 (6.7)	0.21
**Diastolic drop ≥10mmHg 1min**	41 (6.3)	40 (6.0)	0.82
**Systolic drop ≥20mmHg 3 min**	47 (7.2)	42 (6.3)	0.51
**Diastolic drop ≥10mmHg 3min**	46 (7.1)	48 (7.2)	1.00
**Postural Hypotension present***	107 (16.5)	96 (14.4)	0.32
**Systolic inter-arm difference ≥10mmHg**	121 (18.8)	121 (18.1)	0.83
**Previous stroke**	44 (6.8)	45 (6.7)	1.00
**Pre-existing diabetes**	80 (12.3)	76 (11.4)	0.61
**Pre-existing hypertension**	279 (43.0)	292 (43.7)	0.82
**Pre-existing CV disease**	63 (9.7)	50 (7.5)	0.17
**Pre-existing dementia**	38 (5.9)	27 (4.0)	0.16
**Pre-existing Parkinson's disease**	9 (1.4)	6 (0.9)	0.45
**Fall in preceding 12 months**	143 (22.0)	130 (19.5)	0.28

#mean of 2nd and 3rd readings
*defined as a drop of ≥20mmHg systolic or ≥10mmHg diastolic within 3 minutes of standing

Table 2. Baseline characteristics of derivation and validation cohorts

For peer review only - http://bmjopen.bmj.com/site/about/guidelines.xhtml

**Variable (n (%) unless otherwise stated)**	**PH absent (n=542)**	**PH present (n=107)**	**p value**
Age (mean, SD)	67.7 (15.8)	72.2 (14.6)	0.005
Age over 60	438 (81)	96 (90)	0.027
Age over 65	421 (78)	90 (84)	0.160
Age over 70	302 (56)	73 (68)	0.018
MMSE score (mean, SD)	25.3 (4.9)	24.1 (5.1)	0.031
Female gender	301 (55.5)	67 (62.6)	0.200
Angiotensin converting enzyme inhibitors	103 (19)	23 (22)	0.552
Angiotensin-2 antagonists	6 (1)	4 (4)	0.066
Calcium channel blockers	62 (11)	15 (14)	0.451
Diuretics	48 (9)	17 (16)	0.027
Beta-blockers	20 (4)	4 (4)	0.981
alpha-blockers	11 (2)	1 (1)	0.442
aldosterone antagonists	2 (0.4)	0 (0)	0.529
Digoxin	27 (5)	14 (13)	0.004
Antiarrhythmics, class I and III	10 (2)	4 (4)	0.264
Psycholeptics: typical antipsychotics	8 (1)	4 (4)	0.119
Psycholeptics: atypical antipsychotics	6 (1)	1 (1)	1.000
Psycholeptics: anxiolytics	103 (19)	18 (17)	0.684
Psychoanaleptics: antidepressants	22 (4)	5 (5)	0.791
Drugs for dementia	5 (1)	0 (0)	1.000
Hypertension	217 (40)	62 (58)	0.001
Congestive heart failure	22 (4)	10 (9)	0.028
Myocardial infarction	23 (4)	6 (6)	0.607
Angina	21 (4)	7 (6)	0.421
Any CV disease	45 (8)	18 (17)	0.011
Stroke	28 (5)	16 (15)	0.001
Diabetes	64 (12)	16 (15)	0.420
Parkinson's disease	4 (1)	5 (5)	0.008
Any cancer	30 (6)	8 (8)	0.497
Dementia	29 (5)	9 (8)	0.257
MMSE score 22 to 26	150 (28)	27 (25)	0.637
hospital admission in past year	54 (10)	18 (17)	0.044
Weight loss ≥10lbs in past year	22 (4)	7 (6)	0.301
Any fall in past year	115 (21)	28 (26)	0.254
Any ADL disability	100 (19)	28 (26)	0.083
WHO disability level >1	66 (12)	24 (23)	0.045
Systolic blood pressure (mean, SD) mmHg	144.3 (20.1)	153.7 (25.3)	<0.001
Diastolic blood pressure (mean, SD) mmHg	82.2 (8.8)	86.2 (8.1)	<0.001

For peer review only - http://bmjopen.bmj.com/site/about/guidelines.xhtml

Systolic inter-arm difference (mean, SD) mmHg	2.0 (4.1)	4.7 (5.9)	<0.001
Systolic inter-arm BP difference ≥10mmHg	81 (15)	40 (37)	<0.001
Systolic inter-arm BP difference ≥ 15mmHg	10 (2)	6 (6)	0.007

p values derived from t-tests for continuous data, or Pearson chi-square for categorical data; Fisher's exact test reported where expected cell count <5

Table 3. Univariable associations of risk markers with postural hypotension in derivation cohort

For peer review only

Variable	Odds Ratio	95% Confidence Interval
Model 1		
Parkinson's disease	4.7	1.2 to 19.2
Previous stroke	2.2	1.1 to 4.5
Taking digoxin	2.2	1.0 to 4.7
Previous cardiac disease	1.9	1.0 to 3.6
Hypertension	1.7	1.1 to 2.6
Systolic inter-arm difference (continuous per mmHg)	1.1	1.1 to 1.2
Model 2		
Parkinson's disease	5.0	1.2 to 19.9
Previous stroke	2.2	1.1 to 4.4
Taking digoxin	2.4	1.1 to 5.1
Previous cardiac disease	1.9	1.0 to 2.6
Hypertension	1.7	1.1 to 5.1
Systolic inter-arm difference ≥10mmHg	3.3	2.0 to 5.3
Model 3		
Parkinson's disease	5.3	1.4 to 20.4
Previous stroke	2.4	1.2 to 4.8
Taking digoxin	2.0	0.9 to 4.3
Previous cardiac disease	1.8	0.9 to 3.4
Hypertension	1.9	1.3 to 3.0

Table 4. Multivariable prediction models for postural hypotension

For peer review only

For peer review only - http://bmjopen.bmj.com/site/about/guidelines.xhtml

	Including inter-arm difference		Excluding inter-arm difference	
	**Weighted**	**Unweighted**	**Weighted**	**Unweighted**
Prediction of PH per unit increase of DROP score OR (95%CI)	1.9 (1.4 to 2.5)	1.8 (1.4 to 2.3)	2.4 (1.6 to 3.4)	2.0 (1.5 to 2.6)
Area under ROC curve (95%CI)	0.65 (0.59 to 0.70)	0.65 (0.60 to 0.71)	0.65 (0.59 to 0.71)	0.65 (0.59 to 0.70)
Mortality risk per unit score OR (95%CI)	1.9 (1.6 to 2.2)	1.8 (1.5 to 2.1)	2.8 (2.2 to 3.4)	2.1 (1.8 to 2.5)
Change in MMSE score over study (ANOVA)	N/A	P=0.004	N/A	P<0.001
Annual change in MMSE score (ANOVA)	N/A	P<0.001	N/A	P<0.001

Table 5. DROP score associations with postural hypotension, mortality and cognitive decline

For peer review only

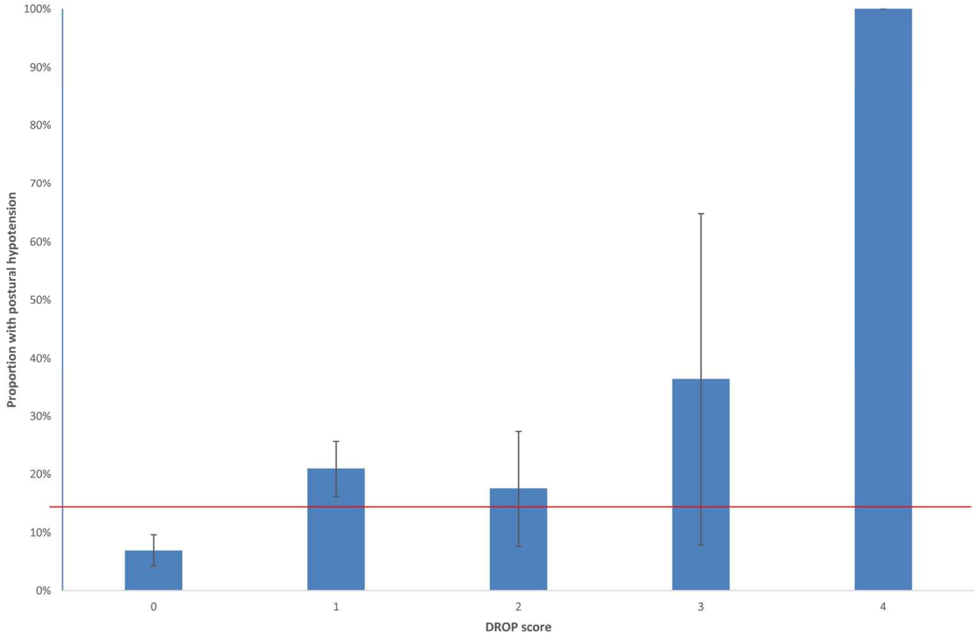

Prevalence of postural hypotension vs unweighted DROP Score without inter-arm difference term (Population prevalence indicated by horizontal line)

167x108mm (300 x 300 DPI)

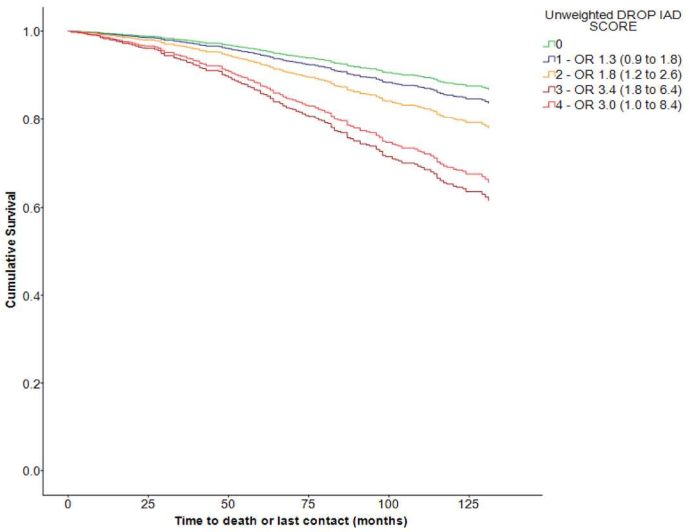

Kaplan Meier survival plot for DROP scores over 9 years follow up

209x148mm (300 x 300 DPI)

For peer review only - http://bmjopen.bmj.com/site/about/guidelines.xhtml

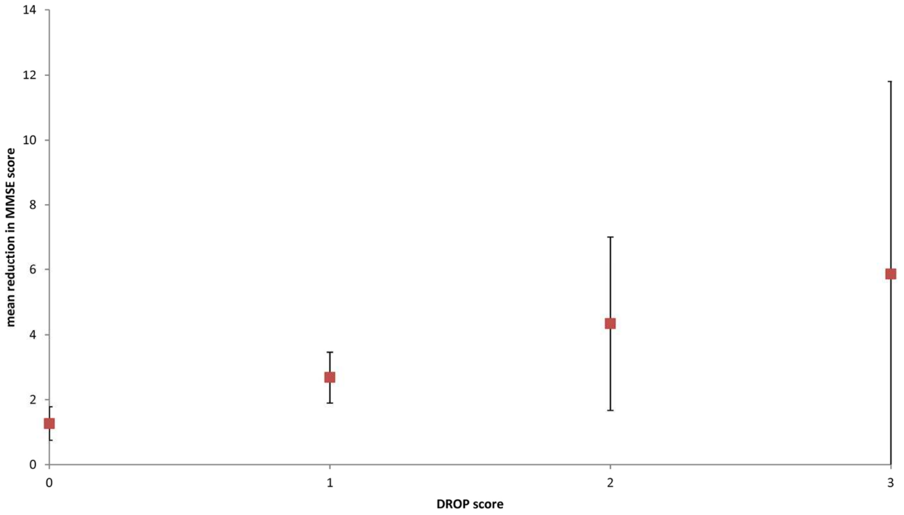

Mean change in Mini Mental State Examination score over nine years per DROP score

165x108mm (300 x 300 DPI)

view only

Appendix: Literature search for factors associated with postural hypotension

Demographics:	Increasing age[1-9]
	Female gender[10]
	Nursing home residence[11-15]
Medical History:	Hypertension[7-10 16-20] and uncontrolled hypertension[6 21 22]
	Diabetes and diabetic complications[17 23-28]
	Chronic Kidney Disease[10 29 30]
	Stroke[31-36]
	Ischaemic heart disease[36 37]
	Heart failure[38 39]
	Parkinson's disease[40-42]
	Cognitive impairment[43-50]
	Depression[51]
Medications:	Antiarrhythmic drugs[11]
	Antihypertensives[4 9-11 52-55] (negative association with ACE inhibitors)[10]
	Psychotropic agents (antipsychotics, sedatives, antidepressants)[23 53 56]
	Anticholinesterase inhibitors[50]
Biochemical:	Vitamin D deficiency (conflicting evidence)[1 23 57 58]
Frailty:[59 60]	Falls[61]
	Get up and go test[11]
	Reduced calf mass index[54 62]
	Activity of Daily Living disability score[1 11]
	Cumulative illness Rating Scale for Geriatrics score[23]
Environmental:	Seasons – prevalence higher in summer and in heatwaves[63 64]
	Time of day – higher in mornings[65-68]

For peer review only - http://bmjopen.bmj.com/site/about/guidelines.xhtml

References

1. Soysal P, Yay A, Isik AT. Does vitamin D deficiency increase orthostatic hypotension risk in the elderly patients? *Archives of Gerontology and Geriatrics* 2014;59(1):July. doi: http://dx.doi.org/10.1016/j.archger.2014.03.008
2. Moret F, Jaccard-Ruedin H, Bula C, et al. The high diagnostic yield of an outpatient geriatric clinic. *Journal of the American Geriatrics Society Conference: 2012 Annual Scientific Meeting of the American Geriatrics Society Seattle, WA United States Conference Start: 20120503 Conference End: 20120505 Conference Publication: (var pagings)* 2012;60(pp S175):April. doi: http://dx.doi.org/10.1111/j.1532-5415.2012.04000.x
3. Robertson D, Desjardin JA, Lichtenstein MJ. Distribution and observed associations of orthostatic blood pressure changes in elderly general medicine outpatients. *American Journal of the Medical Sciences* 1998;315(5):1998. doi: http://dx.doi.org/10.1097/00000441-199805000-00001
4. Poon IO, Braun U. High prevalence of orthostatic hypotension and its correlation with potentially causative medications among elderly veterans. *Journal of Clinical Pharmacy and Therapeutics* 2005;30(2):April. doi: http://dx.doi.org/10.1111/j.1365-2710.2005.00629.x
5. Mendez CA, Melgarejo JD, Lee JH, et al. Orthostatic hypotension in latino elderly: Findings from the maracaibo ageing study. *Journal of Hypertension Conference: 25th European Meeting on Hypertension and Cardiovascular Protection, ESH 2015 Milan Italy Conference Start: 20150612 Conference End: 20150615 Conference Publication: (var pagings)* 2015;33(pp e219):June. doi: http://dx.doi.org/10.1097/01.hjh.0000468017.03671.63
6. Barochiner J, Alfie J, Aparicio L, et al. Orthostatic hypotension in treated hypertensive patients. *Romanian journal of internal medicine = Revue roumaine de medecine interne* 2012;50(3):2012-2Sep.
7. Shin C, Abbott RD, Lee H, et al. Prevalence and correlates of orthostatic hypotension in middle-aged men and women in Korea: The Korean Health and Genome Study. *Journal of Human Hypertension* 2004;18(10):October. doi: http://dx.doi.org/10.1038/sj.jhh.1001732
8. Harris T, Lipsitz LA, Kleinman JC, et al. Postural change in blood pressure associated with age and systolic blood pressure. The National Health and Nutrition Examination Survey II. *Journals of Gerontology* 1991;46(5):1991.
9. Kamaruzzaman S, Watt H, Carson C, et al. The association between orthostatic hypotension and medication use in the British Women's Heart and Health Study. *Age and Ageing* 2010;39(1):afp192. doi: http://dx.doi.org/10.1093/ageing/afp192
10. Fedorowski A, Burri P, Melander O. Orthostatic hypotension in genetically related hypertensive and normotensive individuals. *Journal of Hypertension* 2009;27(5):May. doi: http://dx.doi.org/10.1097/HJH.0b013e3283279860
11. Boland E, Brackelaire G, Anani Y, et al. Postprandial hypotension among patients from an acute geriatric ward: Results from a pilot study. *European Geriatric Medicine Conference: 11th International Congress of the European Union Geriatric Medicine Society, EUGMS 2015 Oslo Norway Conference Start: 20150916 Conference End: 20150918 Conference Publication: (var pagings)* 2015;6(pp S64):September.
12. Hartog LC, Cizmar-Sweelssen M, Knipscheer A, et al. The association between orthostatic hypotension, falling and successful rehabilitation in a nursing home population. *Archives of Gerontology and Geriatrics* 2015;61(2):01. doi: http://dx.doi.org/10.1016/j.archger.2015.05.005
13. Asensio LE, Aguilera AAC, Corral MACC, et al. Prevalence of orthostatic hypotension in a series of elderly institutionalized patients. *Europace Conference: EHRA Europace 2011 Madrid Spain Conference Start: 20110626 Conference End: 20110629 Conference Publication: (var pagings)* 2011;13 doi: http://dx.doi.org/10.1093/europace/eur231

For peer review only - http://bmjopen.bmj.com/site/about/guidelines.xhtml

14. Matusik P, Nowak J, Tomaszewski K, et al. Hypertension among the elderly on the basis of nursing home residents population. [Polish]. *Polski Przeglad Kardiologiczny* 2010;12(3):2010.
15. Wu J-S, Yang Y-C, Lu F-H, et al. Population-based study on the prevalence and correlates of orthostatic hypotension/hypertension and orthostatic dizziness. *Hypertension Research* 2008;31(5):May. doi: http://dx.doi.org/10.1291/hypres.31.897
16. Saez T, Suarez C, Sierra MJ, et al. Orthostatic hypotension in the elderly and its association with the antihypertensive treatment. [Spanish]. *Medicina Clinica* 2000;114(14):15.
17. Wu JS, Yang YC, Lu FH, et al. Population-based study on the prevalence and risk factors of orthostatic hypotension in subjects with pre-diabetes and diabetes. *Diabetes Care* 2009;32(1):69-74. doi: http://dx.doi.org/10.2337/dc08-1389
18. Fan X-H, Sun K, Zhou X-L, et al. Association of orthostatic hypertension and hypotension with target organ damage in middle and old-aged hypertensive patients. [Chinese]. *National Medical Journal of China* 2011;91(4):04. doi: http://dx.doi.org/10.3760/cma.j.issn.0376-2491.2011.04.002
19. Barochiner J, Alfie J, Aparicio LS, et al. Prevalence and clinical profile of resistant hypertension among treated hypertensive subjects. *Clinical and Experimental Hypertension* 2013;35(6):2013. doi: http://dx.doi.org/10.3109/10641963.2012.739236
20. Bouhanick B, Meliani S, Doucet J, et al. Orthostatic hypotension is associated with more severe hypertension in elderly autonomous diabetic patients from the French Gerodiab study at inclusion. *Annales de Cardiologie et d Angeiologie* 2014;63(3):176-82. doi: http://dx.doi.org/10.1016/j.ancard.2014.05.013
21. Auseon A, Ooi WL, Hossain M, et al. Blood pressure behavior in the nursing home: Implications for diagnosis and treatment of hypertension. *Journal of the American Geriatrics Society* 1999;47(3):March.
22. Masuo K, Mikami H, Ogihara T, et al. Changes in frequency of orthostatic hypotension in elderly hypertensive patients under medications. *American Journal of Hypertension* 1996;9(3):March. doi: http://dx.doi.org/10.1016/0895-7061%2895%2900348-7
23. Paccalin M. Factors associated with orthostatic hypotension in hospitalized elderly patients. *European Geriatric Medicine Conference: 11th International Congress of the European Union Geriatric Medicine Society, EUGMS 2015 Oslo Norway Conference Start: 20150916 Conference End: 20150918 Conference Publication: (var pagings)* 2015;6(pp S62):September.
24. van Hateren KJJ, Kleefstra N, Blanker MH, et al. Orthostatic hypotension, diabetes, and falling in older patients: A cross-sectional study. *British Journal of General Practice* 2012;62(603):October. doi: http://dx.doi.org/10.3399/bjgp12X656838
25. Gupta A, Gilden J. Prevalence of diabetes in patients admitted to the hospital with primary diagnosis of orthostatic hypotension. *Diabetes Conference: 73rd Scientific Sessions of the American Diabetes Association Chicago, IL United States Conference Start: 20130621 Conference End: 20130625 Conference Publication: (var pagings)* 2013;62(pp A148):July. doi: http://dx.doi.org/10.2337/db13-388-679
26. Eze CO, Onwuekwe IO, Agu CE, et al. The prevalence of orthostatic hypotension in type 2 diabetes mellitus patients in a diabetic clinic in Enugu South-East Nigeria. *Nigerian journal of medicine : journal of the National Association of Resident Doctors of Nigeria* 2013;22(3):2013-2Sep.
27. Lanthier L, Touchette M, Bourget P, et al. [Evaluation of circadian variation of blood pressure by ambulatory blood pressure monitoring in an elderly diabetic population with or without orthostatic hypotension]. [French]. *Geriatrie et psychologie neuropsychiatrie du vieillissement* 2011;9(1):Mar.
28. Rota E, Quadri R, Fanti E, et al. Clinical and electrophysiological correlations in type 2 diabetes mellitus at diagnosis. *Diabetes Research and Clinical Practice* 2007;76(1):April. doi: http://dx.doi.org/10.1016/j.diabres.2006.07.027

For peer review only - http://bmjopen.bmj.com/site/about/guidelines.xhtml

29. Aghera D, McFadden C, Hunter K. Orthostatic hypotension in elderly individuals with chronic kidney disease(CKD). *American Journal of Kidney Diseases Conference: National Kidney Foundation 2015 Spring Clinical Meetings, NKF SCM15 Dallas, TX United States Conference Start: 20150325 Conference End: 20150329 Conference Publication: (var pagings)* 2015;65(4):April.
30. Bhat S, Hegde S, Szpunar S, et al. Prevalence of orthostatic variation in blood pressure among stable outpatient chronic kidney disease population. *American Journal of Kidney Diseases Conference: National Kidney Foundation 2013 Spring Clinical Meetings Orlando, FL United States Conference Start: 20130402 Conference End: 20130406 Conference Publication: (var pagings)* 2013;61(4):April. doi: http://dx.doi.org/10.1053/j.ajkd.2013.02.049
31. Phipps MS, Schmid AA, Kapoor JR, et al. Orthostatic hypotension among outpatients with ischemic stroke. *Journal of the Neurological Sciences* 2012;314(1-2):15. doi: http://dx.doi.org/10.1016/j.jns.2011.10.031
32. Ryan DJ, Kenny RA, Christensen S, et al. Ischaemic stroke or TIA in older subjects associated with impaired dynamic blood pressure control in the absence of severe large artery stenosis. *Age and Ageing* 2015;44(4):afv011. doi: http://dx.doi.org/10.1093/ageing/afv011
33. Eguchi K, Kario K, Hoshide S, et al. Greater change of orthostatic blood pressure is related to silent cerebral infarct and cardiac overload in hypertensive subjects. *Hypertension Research* 2004;27(4):April. doi: http://dx.doi.org/10.1291/hypres.27.235
34. Kario K, Shimada K. Orthostatic blood pressure changes and silent cerebrovascular disease. *Cardiology Review* 2003;20(4):01.
35. Kwok CS, Ong ACL, Potter JF, et al. TIA, stroke and orthostatic hypotension: A disease spectrum related to ageing vasculature? *International Journal of Clinical Practice* 2014;68(6):June. doi: http://dx.doi.org/10.1111/ijcp.12373
36. Rutan GH, Hermanson B, Bild DE, et al. Orthostatic hypotension in older adults. The Cardiovascular Health Study. CHS Collaborative Research Group. *Hypertension* 1992;19(6:Pt 1):t-19.
37. Lin ZQ, Pan CM, Li WH, et al. [The correlation between postural hypotension and myocardial infarction in the elderly population]. [Chinese]. *Zhonghua nei ke za zhi [Chinese journal of internal medicine]* 2012;51(7):Jul.
38. Potocka-Plazak K, Plazak W. Orthostatic hypotension in elderly women with congestive heart failure. *Aging - Clinical and Experimental Research* 2001;13(5):2001.
39. McGann PE. Comorbidity in heart failure in the elderly. *Clinics in Geriatric Medicine* 2000;16(3):2000.
40. Rascol O, Perez-Lloret S, Damier P, et al. Falls in ambulatory non-demented patients with Parkinson's disease. *Journal of Neural Transmission* 2015;122(10):07. doi: http://dx.doi.org/10.1007/s00702-015-1396-2
41. Senard JM, Rai S, Lapeyre-Mestre M, et al. Prevalence of orthostatic hypotension in Parkinson's disease. *Journal of Neurology Neurosurgery and Psychiatry* 1997;63(5):November.
42. Palma J-A, Gomez-Esteban JC, Norcliffe-Kaufmann L, et al. Orthostatic Hypotension in Parkinson Disease: How Much You Fall or How Low You Go? *Movement Disorders* 2015;30(5):15. doi: http://dx.doi.org/10.1002/mds.26079
43. Elmstahl S, Widerstrom E. Orthostatic intolerance predicts mild cognitive impairment: incidence of mild cognitive impairment and dementia from the Swedish general population cohort Good Aging in Skane. *Clinical interventions in aging* 2014;9(pp 1993-2002):2014. doi: http://dx.doi.org/10.2147/CIA.S72316
44. Frewen J, Savva GM, Boyle G, et al. Cognitive performance in orthostatic hypotension: Findings from a nationally representative sample. *Journal of the American Geriatrics Society* 2014;62(1):January. doi: http://dx.doi.org/10.1111/jgs.12592
45. Frewen J, Finucane C, Savva GM, et al. Orthostatic hypotension is associated with lower cognitive performance in adults aged 50 plus with supine hypertension. *The journals of gerontology*

For peer review only - http://bmjopen.bmj.com/site/about/guidelines.xhtml

Series A, Biological sciences and medical sciences 2014;69(7):Jul. doi: http://dx.doi.org/10.1093/gerona/glt171

46. Traykova M, Stankova T, Mehrabian S, et al. High prevalence of orthostatic hypotension in vascular and degenerative dementia. *European Journal of Medical Research Conference: 21st European Students' Conference Promising Medical Scientists Willing to Look Beyond Berlin Germany Conference Start: 20101013 Conference End: 20101017 Conference Publication: (var pagings)* 2010;15(pp 126-127):13.
47. Yap PL, Niti M, Yap KB, et al. Orthostatic hypotension, hypotension and cognitive status: early comorbid markers of primary dementia? *Dementia & Geriatric Cognitive Disorders* 2008;26(3):239-46. doi: http://dx.doi.org/10.1159/000160955
48. Sonnesyn H, Nilsen DW, Rongve A, et al. High prevalence of orthostatic hypotension in mild dementia. *Dementia and Geriatric Cognitive Disorders* 2009;28(4):November. doi: http://dx.doi.org/10.1159/000247586
49. Campbell AJ, Reinken J. Postural hypotension in old age: Prevalence, associations and prognosis. *Journal of Clinical and Experimental Gerontology* 1985;7(2):1985.
50. Isik AT, Soysal P, Mas M. Orthostatic hypotension and long-term effects of acheis on the orthostatic hypotension in elderly patients with alzheimer disease. *Alzheimer's and Dementia Conference: Alzheimer's Association International Conference 2014 Copenhagen Denmark Conference Start: 20140712 Conference End: 20140717 Conference Publication: (var pagings)* 2014;10(pp P774):July.
51. Regan CO, Kearney PM, Cronin H, et al. Oscillometric measure of blood pressure detects association between orthostatic hypotension and depression in population based study of older adults. *BMC psychiatry* 2013;13(pp 266):2013. doi: http://dx.doi.org/10.1186/1471-244X-13-266
52. Ruwald MH, Hansen ML, Lamberts M, et al. Comparison of incidence, predictors, and the impact of co-morbidity and polypharmacy on the risk of recurrent syncope in patients <85 versus >85 years of age. *American Journal of Cardiology* 2013;112(10):15. doi: http://dx.doi.org/10.1016/j.amjcard.2013.07.041
53. Pepersack T, Gilles C, Petrovic M, et al. Prevalence of orthostatic hypotension and relationship with drug use amongst older patients. *Acta clinica Belgica* 2013;68(2):2013-2Apr.
54. Kobayashi K, Yamada S. Development of a simple index, calf mass index, for screening for orthostatic hypotension in community-dwelling elderly. *Archives of Gerontology and Geriatrics* 2012;54(2):March. doi: http://dx.doi.org/10.1016/j.archger.2011.04.003
55. Di SC, Milazzo V, Bruno G, et al. Prevalence of orthostatic hypotension in a cohort of patients under antihypertensive therapy. *High Blood Pressure and Cardiovascular Prevention Conference: 2013 National Congress of the Italian Society of Hypertension, SIIA 2013 Rome Italy Conference Start: 20131003 Conference End: 20131005 Conference Publication: (var pagings)* 2013;20(3):September. doi: http://dx.doi.org/10.1007/s40292-013-0021-4
56. Rozenfeld S, Bastos Camacho LA, Peixoto VR. Medication as a risk factor for falls in older women in Brazil. *Revista Panamericana de Salud Publica/Pan American Journal of Public Health* 2003;13(6):01.
57. Soysal P, Yay A, Isik AT. Does 25-hydroxyvitamin D deficiency increase orthostatic hypotension risk in the elderly patients? *European Geriatric Medicine Conference: 10th International Congress of the European Union Geriatric Medicine Society - Geriatric Medicine Crossing Borders, EUGMS 2014 Rotterdam Netherlands Conference Start: 20140917 Conference End: 20140919 Conference* 2014;5(pp S120):September.
58. Veronese N, Bolzetta F, De RM, et al. Serum 25-hydroxyvitamin D and orthostatic hypotension in old people: The Pro.V.A. study. *Hypertension* 2014;64(3):September. doi: http://dx.doi.org/10.1161/HYPERTENSIONAHA.114.03143

For peer review only - http://bmjopen.bmj.com/site/about/guidelines.xhtml

59. O'Connell MDL, Savva GM, Fan CW, et al. Orthostatic hypotension, orthostatic intolerance and frailty: The Irish Longitudinal Study on Aging-TILDA. *Archives of Gerontology and Geriatrics* 2015;60(3):01. doi: http://dx.doi.org/10.1016/j.archger.2015.01.008
60. Rockwood MR, Howlett SE, Rockwood K. Orthostatic hypotension (OH) and mortality in relation to age, blood pressure and frailty. *Archives of Gerontology & Geriatrics* 2012;54(3):e255-e60. doi: http://dx.doi.org/10.1016/j.archger.2011.12.009
61. Lagro J, Laurenssen NCW, Schalk BWM, et al. Diastolic blood pressure drop after standing as a clinical sign for increased mortality in older falls clinic patients. *Journal of Hypertension* 2012;30(6):June. doi: http://dx.doi.org/10.1097/HJH.0b013e328352b9fd
62. Madhavan G, Goddard AA, McLeod KJ. Prevalence and Etiology of Delayed Orthostatic Hypotension in Adult Women. *Archives of Physical Medicine and Rehabilitation* 2008;89(9):September. doi: http://dx.doi.org/10.1016/j.apmr.2008.02.021
63. Weiss A, Beloosesky Y, Grinblat J, et al. Seasonal changes in orthostatic hypotension among elderly admitted patients. *Aging Clinical and Experimental Research* 2006;18(1):February.
64. Pathak A, Lapeyre-Mestre M, Montastruc J-L, et al. Heat-related morbidity in patients with orthostatic hypotension and primary autonomic failure. *Movement Disorders* 2005;20(9):September. doi: http://dx.doi.org/10.1002/mds.20571
65. Ooi WL, Barrett S, Hossain M, et al. Patterns of orthostatic blood pressure change and their clinical correlates in a frail, elderly population. *Journal of the American Medical Association* 1997;277(16):23.
66. Ward C, Kenny RA. Reproducibility of orthostatic hypotension in symptomatic elderly. *American Journal of Medicine* 1996;100(4):April. doi: http://dx.doi.org/10.1016/S0002-9343%2897%2989517-4
67. Weiss A, Grossman E, Beloosesky Y, et al. Orthostatic hypotension in acute geriatric ward: Is it a consistent finding? *Archives of Internal Medicine* 2002;162(20):15. doi: http://dx.doi.org/10.1001/archinte.162.20.2369
68. Youde JH, Manktelow B, Ward-Close S, et al. Measuring postural changes in blood pressure in the healthy elderly. *Blood Pressure Monitoring* 1999;4(1):1999.

For peer review only

For peer review only - http://bmjopen.bmj.com/site/about/guidelines.xhtml

Medline and Embase Search Strategy

Date of search 20th October 2015

	Searches	Results
1	postural hypotension.mp. [mp=ti, ab, hw, tn, ot, dm, mf, dv, kw, nm, kf, px, rx, an, ui]	3109
2	orthostatic hypotension.mp. [mp=ti, ab, hw, tn, ot, dm, mf, dv, kw, nm, kf, px, rx, an, ui]	22694
3	1 or 2	21615
4	prevalence.mp. [mp=ti, ab, hw, tn, ot, dm, mf, dv, kw, nm, kf, px, rx, an, ui]	1202953
5	3 and 4	1678
6	limit 5 to humans	1565
7	limit 6 to aged <65+ years> [Limit not valid in Ovid MEDLINE(R),Ovid MEDLINE(R) In-Process; records were retained]	661
8	remove duplicates from 7	470

For peer review only - http://bmjopen.bmj.com/site/about/guidelines.xhtml

TRIPOD Checklist: Prediction Model Development and Validation

Section/Topic	Item		Checklist Item	Page
**Title and abstract**				
Title	1	D;V	Identify the study as developing and/or validating a multivariable prediction model, the target population, and the outcome to be predicted.	1
Abstract	2	D;V	Provide a summary of objectives, study design, setting, participants, sample size, predictors, outcome, statistical analysis, results, and conclusions.	3
**Introduction**				
Background and objectives	3a	D;V	Explain the medical context (including whether diagnostic or prognostic) and rationale for developing or validating the multivariable prediction model, including references to existing models.	4
	3b	D;V	Specify the objectives, including whether the study describes the development or validation of the model or both.	4
**Methods**				
Source of data	4a	D;V	Describe the study design or source of data (e.g., randomized trial, cohort, or registry data), separately for the development and validation data sets, if applicable.	4
	4b	D;V	Specify the key study dates, including start of accrual; end of accrual; and, if applicable, end of follow-up.	4
Participants	5a	D;V	Specify key elements of the study setting (e.g., primary care, secondary care, general population) including number and location of centres.	4
	5b	D;V	Describe eligibility criteria for participants.	4
	5c	D;V	Give details of treatments received, if relevant.	N/A
Outcome	6a	D;V	Clearly define the outcome that is predicted by the prediction model, including how and when assessed.	5
	6b	D;V	Report any actions to blind assessment of the outcome to be predicted.	5
Predictors	7a	D;V	Clearly define all predictors used in developing or validating the multivariable prediction model, including how and when they were measured.	5 & table 1
	7b	D;V	Report any actions to blind assessment of predictors for the outcome and other predictors.	5
Sample size	8	D;V	Explain how the study size was arrived at.	6
Missing data	9	D;V	Describe how missing data were handled (e.g., complete-case analysis, single imputation, multiple imputation) with details of any imputation method.	N/A
Statistical analysis methods	10a	D	Describe how predictors were handled in the analyses.	5
	10b	D	Specify type of model, all model-building procedures (including any predictor selection), and method for internal validation.	5
	10c	V	For validation, describe how the predictions were calculated.	5
	10d	D;V	Specify all measures used to assess model performance and, if relevant, to compare multiple models.	5
	10e	V	Describe any model updating (e.g., recalibration) arising from the validation, if done.	N/A
Risk groups	11	D;V	Provide details on how risk groups were created, if done.	N/A
Development vs. validation	12	V	For validation, identify any differences from the development data in setting, eligibility criteria, outcome, and predictors.	None table 2
**Results**				
Participants	13a	D;V	Describe the flow of participants through the study, including the number of participants with and without the outcome and, if applicable, a summary of the follow-up time. A diagram may be helpful.	5
	13b	D;V	Describe the characteristics of the participants (basic demographics, clinical features, available predictors), including the number of participants with missing data for predictors and outcome.	table 2
	13c	V	For validation, show a comparison with the development data of the distribution of important variables (demographics, predictors and outcome).	table 2
Model development	14a	D	Specify the number of participants and outcome events in each analysis.	5
	14b	D	If done, report the unadjusted association between each candidate predictor and outcome.	table 3
Model specification	15a	D	Present the full prediction model to allow predictions for individuals (i.e., all regression coefficients, and model intercept or baseline survival at a given time point).	table 4
	15b	D	Explain how to the use the prediction model.	6
Model performance	16	D;V	Report performance measures (with CIs) for the prediction model.	6 table 5
Model-updating	17	V	If done, report the results from any model updating (i.e., model specification, model performance).	6
**Discussion**				
Limitations	18	D;V	Discuss any limitations of the study (such as nonrepresentative sample, few events per predictor, missing data).	6-7
Interpretation	19a	V	For validation, discuss the results with reference to performance in the development data, and any other validation data.	6-7
	19b	D;V	Give an overall interpretation of the results, considering objectives, limitations, results from similar studies, and other relevant evidence.	6-7
Implications	20	D;V	Discuss the potential clinical use of the model and implications for future research.	8
**Other information**				
Supplementary information	21	D;V	Provide information about the availability of supplementary resources, such as study protocol, Web calculator, and data sets.	N/A
Funding	22	D;V	Give the source of funding and the role of the funders for the present study.	14

For peer review only

TRIPOD Checklist: Prediction Model Development and Validation

*Items relevant only to the development of a prediction model are denoted by D, items relating solely to a validation of a prediction model are denoted by V, and items relating to both are denoted D;V. We recommend using the TRIPOD Checklist in conjunction with the TRIPOD Explanation and Elaboration document.

For peer review only

BMJ Open

Detecting Risks Of Postural Hypotension (DROP): derivation and validation of a prediction score for primary care

Journal:	*BMJ Open*
Manuscript ID	bmjopen-2017-020740.R2
Article Type:	Research
Date Submitted by the Author:	07-Feb-2018
Complete List of Authors:	Clark, Christopher; University of Exeter, Primary Care Research Group<br>Thomas, Daniel; University of Exeter, Primary Care Research Group<br>Warren, Fiona; University of Exeter Medical School, Institute of Health Research<br>Llewellyn, David; University of Exeter, Mental Health Research Group<br>Ferrucci, Luigi; Harbor Hospital, Baltimore, National Institute on Aging NIA-ASTRA Unit<br>Campbell, John; University of Exeter, Primary Care;
**Primary Subject Heading**:	General practice / Family practice
Secondary Subject Heading:	Cardiovascular medicine, Diagnostics, General practice / Family practice
Keywords:	Hypertension < CARDIOLOGY, PRIMARY CARE, PREVENTIVE MEDICINE

SCHOLARONE™
Manuscripts

For peer review only

For peer review only - http://bmjopen.bmj.com/site/about/guidelines.xhtml

Detecting Risks Of Postural Hypotension (DROP): derivation and validation of a prediction score for primary care

Clark CE, Thomas D, Warren F, Llewellyn D, Ferrucci L, Campbell JL

Christopher E Clark[1], Daniel Thomas[1], Fiona C Warren[1], David J. Llewellyn[2], Luigi Ferrucci[3], John L Campbell[1]

1. Primary Care Research Group
Institute of Health Research
University of Exeter Medical School
Smeall Building, St Luke's Campus
Magdalen Rd, Exeter, Devon, England EX1 2LU

2. Mental Health Research Group
Institute of Health Research
University of Exeter Medical School
College House, St Luke's Campus
Magdalen Rd, Exeter, Devon, England EX1 2LU

3. National Institute on Aging, Baltimore, Maryland, USA
251 Bayview Blvd. Room 04C228
Baltimore, MD 21224 - USA

Abstract: 296 words
Text: 3441 words
Tables: 5
Figures: 3
Appendices: 1

Address for correspondence: Dr C E Clark, as above, email: c.e.clark@exeter.ac.uk

For peer review only - http://bmjopen.bmj.com/site/about/guidelines.xhtml

Abstract

Objectives

Falls are a common problem in older people. Postural hypotension contributes to falls but is often asymptomatic. In the absence of symptoms, postural hypotension is only infrequently checked for in clinical practice. We undertook this study to derive, validate and explore the prospective associations of a prediction tool to identify people likely to have unrecognised postural hypotension.

Design and setting

Cross-sectional and prospective multivariable cohort analysis.

Participants

1317 participants of the InCHIANTI study, a population based cohort representative of the older Italian population.

Primary outcome measures

Predictive value of score to suggest presence of postural hypotension,

Methods

Subjects were randomised 1:1 to derivation or validation cohorts. Within the derivation cohort univariable associations for candidate predictors of postural hypotension were tested. Variables with P<0.1 entered multivariable linear regression models. Factors retaining multivariable significance were incorporated into unweighted and weighted DROP scores. These scores were tested in the validation cohort against prediction of postural hypotension, cognitive decline and mortality over nine years' follow up.

Results

Postural hypotension was present in 203 (15.4%) of participants. Factors predicting postural hypotension were: digoxin use, Parkinson's disease, hypertension, stroke or cardiovascular disease, and an inter-arm systolic blood pressure difference. Area under the curve was consistent at 0.65 for all models, with significant odds ratios (OR) of 1.8 to 2.4 per unit increase in score for predicting postural hypotension. For a DROP score ≥1, five cases need to be tested to identify one with postural hypotension.

Increasing DROP scores predicted mortality (OR 1.8 to 2.8 per unit rise) and increasing rates of decline of Mini Mental State Examination score (ANOVA p<0.001) over 9 years of follow up.

Conclusions

The DROP score provides a simple method to identify people likely to have postural hypotension, and increased risks to health, who require further evaluation.

(296 words)

For peer review only

Strengths and limitations of this study

- This study used data from a well-established cohort representative of an older population in Italy, to derive and validate a score ("DROP score") to predict the presence of postural hypotension.

- Comprehensive recording of baseline variables at recruitment by the InCHIANTI investigators allowed a large number of previously reported risk markers for postural hypotension to be tested in the analyses.

- The study was undertaken according to TRIPOD guidelines and randomised splitting of the cohort allowed internal validation of the findings to be undertaken.

- We chose the consensus definition of postural hypotension as our outcome measure since we sought to predict this, rather than study postural symptoms. Specific postural symptoms were not recorded during recruitment to the InCHIANTI study, and their presence should in any event trigger testing for postural hypotension.

- The population studied did not include residential or nursing home residents; refinement of the scoring system within larger cohorts more representative of primary care populations is required to confirm the potential of the DROP score in practice.

For peer review only

Introduction

Falls are a major cause of morbidity and mortality in older people; 35% of people older than 65 and 50% of people older than 80 fall at least once a year.[1 2] Falls are the leading cause of disability and the leading cause of death from injury among people over 75 in the UK, and cost the NHS around £2.3 billion per year.[3] Postural or orthostatic hypotension is a major risk factor for falls,[4 5] and is independently associated with increased mortality rates.[6-8] Postural hypotension has also been associated with dementia and cognitive impairment, and may have more subtle adverse effects on wellbeing and cognition.[9]

Postural hypotension is commonly defined as a fall of either ≥20mmHg in systolic blood pressure or ≥10mmHg in diastolic blood pressure, from sitting or lying, within three minutes of standing up.[10] Reported prevalences of postural hypotension vary widely, and are sensitive to both care setting, occurring in over half of patients admitted to care of the elderly,[11-13] and to the presence of co-morbidity. General adult population prevalence appears to be around 7%,[14 15] rising to 11 to 15% in persons 65 years old and older,[16-18] and 19% in those aged over 80 or older.[15] Prevalence is reported to be higher in the presence of hypertension,[19-23] stroke,[24 25] myocardial infarction,[25 26] and diabetes.[22 27]

Guidelines vary in recommendations for the detection of postural hypotension. The National Institute for Health and Care Excellence (NICE) recommends testing in the presence of symptoms whist the European Society for Hypertension also recommends testing in the elderly and in the presence of diabetes.[1 28] Unfortunately most individuals with postural hypotension are asymptomatic,[7] and we have found that, in practice, postural hypotension is seldom looked for in patients who do not report postural symptoms.[29] Anecdotally, testing is not undertaken due to time constraints; screening for postural hypotension is not supported in the literature, being regarded as lacking an evidence base, and primary care workloads are rising. [30 31] Risks of hospitalisation, nursing home admission or mortality can already be predicted by the electronic frailty index (eFI), a score derived from existing information in primary care computer records, and incorporated into many general practice computing systems. However the association of eFI with, and its ability to predict, postural hypotension (which itself is poorly tested for and recorded in primary care) is unclear,[32] and comparable frailty indices have not been found to be predictive of postural hypotension.[33] To address this gap in care we hypothesised that a simple prediction score, based on easily recognised risk markers, might help clinicians identify those most likely to have postural hypotension thereby allowing a targeted implementation of sitting and standing blood pressure measurement in the absence of symptoms. We therefore undertook the current analysis, in a well-documented cohort known to be representative of an older population living in the community. Aims were to explore the feasibility of deriving and internally validating a prediction score, to assess its value and its prospective associations.

Methods

The study was conducted and reported in accordance with the TRIPOD statement.[34] We studied participants from the InCHIANTI study; a cohort study designed to explore declining mobility in later life. The Italian National Research Council on Aging ethical committee approved the InCHIANTI study protocol, and the current analysis proposals were approved by the investigating committee of the InCHIANTI study.

The InCHIANTI study methods have been described in detail elsewhere.[35] In brief, 1270 participants aged 65 years or more were randomly selected from the population registries of two villages: Greve

For peer review only - http://bmjopen.bmj.com/site/about/guidelines.xhtml

in Chianti, and Antella in Bagno a Ripoli. Additional people were randomly selected from these sites to complete recruitment of at least 30 men and 30 women for each age decile from age 20 to 29 upwards. Extensive baseline interviews and examinations were conducted at recruitment, between September 1998 and March 2000, and follow up data were obtained after three, six and nine years. Blood pressure was initially measured supine, sequentially in both arms, to identify the higher reading arm, then a further two measurements were made on the higher reading arm. Subjects then stood and blood pressure was measured once after 1 minute and once more after 3 minutes standing. All measurements were obtained by research assistants using a standard mercury sphygmomanometer. Written informed consent was obtained from all participants at recruitment to the InCHIANTI study.

Baseline blood pressure was calculated as the mean of the second and third supine blood pressure readings.[36] Postural changes in blood pressure from lying to standing were calculated by subtraction of this mean from the standing blood pressure. Postural hypotension was considered to exist where there was as a reduction in blood pressure on standing of ≥20mmHg systolic or ≥10mmHg diastolic after 1 or after 3 minutes.[10] Hypertension was defined as use of antihypertensive drugs and/or a documented history of hypertension at recruitment.

For this analysis, participants were randomly allocated in a 1:1 ratio using a split-sample method,[37] stratified for gender and study site, to either a *derivation* or a *validation* group by a statistician (FW) blinded to postural hypotension status and medical history. A literature review was undertaken to identify potential risk markers for consideration in the analyses (appendix). These were mapped to variables available in the InCHIANTI dataset (table 1), which were then tested in the derivation cohort for univariable associations with postural hypotension, using t-tests or χ^2 tests as appropriate to the data. Variables signalling potential univariable associations (defined as $p<0.1$) were included in multivariable model analyses using an automated backward stepwise regression method.[38] We also included age (explored both continuously and as a dichotomous variable with cut-offs of 60, 65 and 70 years) and gender in all multivariable models. Prospective associations of postural hypotension with survival up to 9 years of follow-up were tested using Kaplan-Meier plots and Cox proportional hazard ratios. Cognitive decline was defined as a reduction in Mini Mental State Examination score (MMSE score) of 5 points or more from baseline, and rate of cognitive decline was defined as change in MMSE scores averaged per year of follow up.

Risk markers that retained significance in the multivariable models were used to derive both weighted and unweighted scores (DROP scores); weighted scores were derived by the addition of the multivariable Log (n) odds ratio (OR) for each marker present, whereas the unweighted model allocated one point for each risk marker present. Scores were tested in the validation cohort for ability to predict postural hypotension using ROC analysis, to predict future mortality using Cox proportional hazard ratios, and cognitive decline over nine years using ANOVA. All analyses were undertaken using IBM SPSS Statistics v24.0.0.2.

Results

Data for standing blood pressure existed for 1317 of the 1453 participants (91%) and they formed the cohort for this study. The derivation cohort (n=649) and validation cohort (n=668) were well matched for all important characteristics and putative risk markers (table 2); overall postural

For peer review only - http://bmjopen.bmj.com/site/about/guidelines.xhtml

hypotension was present for 203 (15.4%) of participants at recruitment. Mean age of participants was 68.3 (standard deviation 15.5).

For the derivation cohort postural hypotension was associated, over 9 years of follow-up, with increased all-cause mortality (Hazard Ratio (HR) 1.9; 95% confidence interval (95%CI) 1.4 to 2.7), cardiovascular mortality (HR 2.1; 95%CI 1.2 to 3.4), and non-cardiovascular mortality (HR 2.0; 95%CI 1.3 to 3.0). Results of univariable testing are summarised in table 3. Using a cut off value of $p<0.1$ the following candidate predictors were entered into multivariable models: age (continuous, or dichotomous for age 60 or 70 cut offs), MMSE score, angiotensin 2 antagonist, diuretic and digoxin use, presence of hypertension, any cardiovascular disease (composite of history of myocardial infarction, angina pectoris or congestive heart failure), stroke, Parkinson's disease, hospital admission within the last year, WHO disability level, any disability in activities of daily living, systolic inter-arm difference (continuous or using ≥10mmHg cut off).

Terms for systolic and diastolic blood pressure were entered into the multivariable model in a sensitivity analysis. Apart from finding that systolic blood pressure replaced the term for presence of hypertension, model outputs were unchanged. Therefore we adopted the latter for consistency with our aim to derive a pragmatic score.

Backward stepwise regression analysis produced consistent findings with any permutation of discrete and continuous variables for age (which was not retained in any model) or for inter-arm difference (model 1 and model 2; table 4). Consequently, a dichotomous cut off for inter-arm difference of ≥10mmHg was selected for simplicity, and retained with five other factors (use of digoxin, Parkinson's disease, previous stroke, previous cardiac disease and diagnosis of hypertension) to derive weighted (using log OR) and unweighted (score 1 for each factor present; possible range 0 to 6) *DROP* scores. The scores were tested in the validation cohort. Since inter-arm difference is not routinely measured a third model excluding inter-arm difference (model 3, table 4) was also used to derive DROP scores without this term (possible range 0 to 5).

All versions of the DROP score were found to predict postural hypotension in the validation cohort with similar areas under the curve of 0.65 but a trend to higher odds of postural hypotension with the exclusion of inter-arm difference from the model (Figure 1, table 5). Sensitivities and specificities of the unweighted DROP score without the inter-arm difference term were 76%, 16%, 5% and 53%, 91%, 99% respectively for cut-offs of ≥1, ≥2 and ≥3, although only 15 participants attained a DROP score of 3 and only one a score of 4. This equated to a number needed to test in order to detect one case of postural hypotension of 5, 5 and 2 for DROP scores of 1, 2 and 3 respectively. For the weighted DROP score without inter-arm difference a cut off value of 0.6 or more had a sensitivity of 74% and specificity of 55% for detection of postural hypotension. A similar pattern was seen for the DROP models including inter-arm difference; for an unweighted DROP score of 1 or more sensitivity and specificity for postural hypotension were 81% and 46% respectively predicting detection of one case of postural hypotension for every five tested. For the weighted score, a cut off value of 0.26 had a sensitivity of 81% and a specificity of 46% for detection of postural hypotension.

DROP scores were predictive of mortality over nine years of follow-up, with increasing ORs according to DROP score with adjustment for age (Figure 2). Data on MMSE were available for 529/668 (79%) of the validation cohort; classification by unweighted DROP scores was also predictive of decline in MMSE after nine years (Figure 3). DROP scores were not predictive of future falls; however increasing DROP scores were associated with rising prevalence of falls in the year prior to recruitment (chi^2 for trend $p<0.001$).

For peer review only - http://bmjopen.bmj.com/site/about/guidelines.xhtml

Discussion

Main findings

This analysis has confirmed that it is feasible, in a community living cohort of predominantly older people, to derive a score based on easily recognised risk markers that can help to identify older persons that are likely to have postural hypotension and require further clinical evaluation. The score, consisting of six risk markers: use of digoxin, presence of Parkinson's disease, hypertension, cardiovascular disease, stroke, and a difference in systolic blood pressure between arms ≥10mmHg, performs similarly with or without weighting, therefore a simple additive score is preferred. Performance is also similar when the inter-arm term is omitted, further simplifying its application.

In this population, postural hypotension is associated with a doubling of risk of death over nine years of follow-up. The DROP score also predicts increasing future mortality from any cause and is associated with greater decline in Mini Mental State Examination scores.

Strengths and weaknesses

The cohort was chosen as representative of a free-living elderly population and the 15.4% prevalence of postural hypotension is consistent with figures ranging from 11 to 15% in other general elderly (over 65) populations.[16-18] Comprehensive recording of baseline variables allowed a large number of previously reported risk factors for postural hypotension to be tested. Since this was undertaken as a feasibility study no formal sample size calculation was undertaken, however there were sufficient events to support the multivariable analyses performed.[38] Although the relatively low numbers attaining DROP scores higher than 2 did lead to imprecision around the predictive values of those higher levels of scores. Re-analysis and external validation in a larger sized cohort could overcome this limitation. Blood pressures were measured supine and standing for this study whereas in practice sitting and standing measurements are commonly recommended.[36] These are less sensitive but more practical in primary care,[39] however a score derived in supine to standing cases of postural hypotension cannot be assumed to perform similarly in the sitting to standing setting. Therefore, we regard this analysis as a feasibility study that supports the concept of a simple pragmatic prediction score to aid daily practice, in need of refinement through larger scale analyses, and exploration in cohorts with sit to stand measurements. Although the DROP score was associated with fall prevalence we did not have data on specific posture induced symptoms, so were unable to examine the relationship of the DROP score with postural symptoms. The presence of symptoms, however, should trigger testing for postural hypotension in any event.[1 29]

Relevance to literature

Postural hypotension has previously been reported as a significant independent predictor of four year all-cause mortality in the Honolulu Heart programme.[6] It also predicted mortality in the Malmo Heart study,[8] but not in the Helsinki ageing study.[40] Frailty was associated with a higher prevalence of postural hypotension in the TILDA study, and adjustment for frailty may influence associations with mortality.[41 42] However no measures of frailty remained predictive of postural hypotension on inclusion in the current multivariable analyses, and a frailty index predicted postural *symptoms* but not postural hypotension within TILDA.[33]

Prevalence of postural hypotension rises with age.[15] Although those with postural hypotension in this study were on average five years older age was not a significant independent predictor of postural hypotension in our models. This may have been in part due to the skewed nature of the age profile in InCHIANTI, although sensitivity analyses excluding those under 65 made no difference (not

For peer review only - http://bmjopen.bmj.com/site/about/guidelines.xhtml

reported). Prevalence of postural hypotension is elevated in association with a history of stroke or TIA,[43-45] cardiovascular disease,[24-26] diabetes,[22 27] or hypertension, which itself affects over 60% of the over 65 age group.[46] Thus the significant factors in our models were all age related conditions which seems the likely explanation for loss of age itself as an independent predictor due to collinearity. Parkinson's disease was the strongest predictor of postural hypotension in our analyses although, affecting only 1.1% of participants, it was also the least common factor. Postural hypotension has previously been reported to have prevalence approaching 50% in some groups of Parkinson's sufferers,[47 48] although only a third of those with postural hypotension report symptoms.[49]

The association of postural hypotension with presence of an inter-arm difference is, to our knowledge, a novel finding. We have previously associated inter-arm difference with white coat effects, which can confound detection of postural hypotension.[50 51] Arterial stiffness is a postulated cause of inter-arm difference,[52] and is also associated with postural hypotension;[53 54] thus inter-arm difference as a proxy measure of arterial stiffness might account for the observed association. Hypotension on ambulatory monitoring and elevated pulse-wave velocity are both associated with cognitive decline, lending further support to the association of inter-arm difference, arterial stiffness, and postural hypotension.[55]

Although postural hypotension is associated with diabetes, and with other complications such as neuropathy, retinopathy and proteinuria,[56] there was no univariable association in this study. Prevalence of postural hypotension in diabetes is associated with complications and duration of disease;[57 58] in this cohort diabetes was present in only 6% of participants, whereas recent data suggest that 25% of adults over the age of 65 in the US have it.[59] Therefore a validation of our models in other larger representative populations is needed.

Postural hypotension has been associated with mild cognitive impariement.[60 61] and reduced cognitive performance.[62] Postural hypotension did not predict cognitive decline in a 2 year prospective study of older Finns,[63] but is predictive over longer follow up.[64] In the current analysis postural hypotension per se was not predictive of cognitive decline over nine years of follow up but the DROP score was. This seems plausible given that it includes a number of risk markers known to be associated with cognitive decline.

Relevance to clinical practice

Testing sitting (or lying) and standing blood pressure takes time and training. The skills of nurses measuring postural hypotension are variable when compared with guidelines;[65] incorrect arm positioning can underestimate postural hypotension,[66] and the alerting reaction can over-estimate it.[67] Early and accurate detection of postural hypotension is a pre-requisite to intervening with medication withdrawal to reduce postural blood pressure drops and their associated risks including falls. Currently symptoms appear to be the main trigger for testing.[29] This should continue, however, a tool to identify which *asymptomatic* patients to test may help to target additional testing to those most likely to benefit. A DROP score of one or more appears to have such potential, and may support proposals that individuals at elevated risk of postural hypotension should be tested.[68]

The strongly cardiovascular composition of the DROP score means that patients will commonly be taking antihypertensive drugs. Potential adverse effects of withdrawing antihypertensive medication to ameliorate postural hypotension are unclear, and medication withdrawal may concern clinicians, carers and patients. Risk of falls rises incrementally with each added orthostatic drug.[69] Prevalence of postural hypotension in hypertension is related to use of cardiovascular drugs (antihypertensive

For peer review only - http://bmjopen.bmj.com/site/about/guidelines.xhtml

agents, vasodilators, diuretics),[70 71] alpha blockers,[72] and the number of antihypertensive drugs used,[73 74] and is associated with resistant or uncontrolled hypertension.[75 76] Successful treatment of blood pressure in the elderly is in fact associated with lower prevalence of postural hypotension,[77 78] but withdrawal of antihypertensive therapy improves postural hypotension.[79 80]

We retained Parkinson's disease in our models due to the strength of the association with postural hypotension, however, on clinical grounds, testing for postural hypotension would be better regarded as integral to any review in Parkinson's disease, given the high prevalence of postural hypotension in this condition.[49]

We sought to develop a pragmatic score to support busy clinicians, faced with a rising workload and increasingly multimorbid caseload.[31] Although measurement of blood pressure in both arms has become more frequent over time it is not part of a routine review.[29 81] Therefore we derived a DROP score omitting inter-arm difference, which performed with similar sensitivity and specificity. For the same reasons, we prefer the unweighted score as a practical aide memoire to recognition of the risk of postural hypotension.

Further research

This study has examined the feasibility of identifying whom should be tested with sitting and standing blood pressure measurements to detect asymptomatic postural hypotension. It seems that a simple pragmatic scoring system can support this. We need to refine and externally validate this approach in larger samples more representative of UK primary care. Further work is needed to examine the feasibility and implications of medication review and antihypertensive withdrawal based on detection of postural hypotension in primary care.

Conclusion

We have described the derivation and validation of a score predicting the presence of postural hypotension. Initial testing suggests this approach to be feasible, and has identified the potential utility of the score in predicting mortality and cognitive decline over a nine year period of follow up. Further validation of the score in larger cohorts of individuals is warranted.

For peer review only

References

1. Falls: Assessment and prevention of falls in older people. London: National Institute for Health and Care Excellence 2013.
2. Blake AJ, Morgan K, Bendall MJ, et al. FALLS BY ELDERLY PEOPLE AT HOME: PREVALENCE AND ASSOCIATED FACTORS. *Age and Ageing* 1988;17(6):365-72. doi: 10.1093/ageing/17.6.365
3. The Importance of Vision in Preventing Falls. London: The College of Optometrists, British Geriatrics Society 2011.
4. Juraschek SP, Daya N, Appel LJ, et al. Orthostatic Hypotension in Middle-Age and Risk of Falls. *American Journal of Hypertension* 2017;30(2):188-95. doi: 10.1093/ajh/hpw108
5. McDonald C, Pearce M, Kerr SR, et al. A prospective study of the association between orthostatic hypotension and falls: Definition matters. *Age and Ageing* 2017;46(3):439-45. doi: http://dx.doi.org/10.1093/ageing/afw227
6. Masaki KH, Schatz IJ, Burchfiel CM, et al. Orthostatic hypotension predicts mortality in elderly men: The Honolulu Heart Program. *Circulation* 1998;98(21):24.
7. Benvenuto LJ, Krakoff LR. Morbidity and Mortality of Orthostatic Hypotension: Implications for Management of Cardiovascular Disease. *American Journal of Hypertension* 2011;24(2):135-44.
8. Fedorowski A, Stavenow L, Hedblad B, et al. Orthostatic hypotension predicts all-cause mortality and coronary events in middle-aged individuals (The Malmo Preventive Project). *European Heart Journal* 2010;31(1):85-91. doi: http://dx.doi.org/10.1093/eurheartj/ehp329
9. Mehrabian S, Duron E, Labouree F, et al. Relationship between orthostatic hypotension and cognitive impairment in the elderly. *J Neurol Sci* 2010;299(1-2):45-8. doi: 10.1016/j.jns.2010.08.056 [published Online First: 2010/09/22]
10. Freeman R, Wieling W, Axelrod FB, et al. Consensus statement on the definition of orthostatic hypotension, neurally mediated syncope and the postural tachycardia syndrome. *Clin Auton Res* 2011;21(2):69-72. doi: 10.1007/s10286-011-0119-5
11. Boland E, Brackelaire G, Anani Y, et al. Postprandial hypotension among patients from an acute geriatric ward: Results from a pilot study. *European Geriatric Medicine Conference: 11th International Congress of the European Union Geriatric Medicine Society, EUGMS 2015 Oslo Norway Conference Start: 20150916 Conference End: 20150918 Conference Publication: (var pagings)* 2015;6(pp S64):September.
12. Potocka-Plazak K, Plazak W. Orthostatic hypotension in elderly women with congestive heart failure. *Aging - Clinical and Experimental Research* 2001;13(5):2001.
13. McGann PE. Comorbidity in heart failure in the elderly. *Clinics in Geriatric Medicine* 2000;16(3):2000.
14. Vara-Gonzalez L, Munoz-Cacho P, De Castro SS. Postural changes in blood pressure in the general population of Cantabria (northern Spain). *Blood Pressure Monitoring* 2008;13(5):October. doi: http://dx.doi.org/10.1097/MBP.0b013e32830d4b33
15. Finucane C, O'Connell MD, Fan CW, et al. Age-related normative changes in phasic orthostatic blood pressure in a large population study: findings from The Irish Longitudinal Study on Ageing (TILDA). *Circulation* 2014;130(20):11. doi: http://dx.doi.org/10.1161/CIRCULATIONAHA.114.009831
16. Atli T, Keven K. Orthostatic hypotension in the healthy elderly. *Archives of Gerontology and Geriatrics* 2006;43(3):November/December. doi: http://dx.doi.org/10.1016/j.archger.2005.12.001
17. Mader SL, Josephson KR, Rubenstein LZ. Low prevalence of postural hypotension among community-dwelling elderly. *Journal of the American Medical Association* 1987;258(11):1987. doi: http://dx.doi.org/10.1001/jama.258.11.1511
18. Vara GL, Dominguez RR, Fernandez RM, et al. Prevalence of orthostatic hypotension in elderly hypertensive patients in primary care. [Spanish]. *Atencion primaria / Sociedad Espanola de Medicina de Familia y Comunitaria* 2001;28(3):2001-2Aug.

For peer review only - http://bmjopen.bmj.com/site/about/guidelines.xhtml

19. Shin C, Abbott RD, Lee H, et al. Prevalence and correlates of orthostatic hypotension in middle-aged men and women in Korea: The Korean Health and Genome Study. *Journal of Human Hypertension* 2004;18(10):October. doi: http://dx.doi.org/10.1038/sj.jhh.1001732
20. Saez T, Suarez C, Sierra MJ, et al. Orthostatic hypotension in the elderly and its association with the antihypertensive treatment. [Spanish]. *Medicina Clinica* 2000;114(14):15.
21. Harris T, Lipsitz LA, Kleinman JC, et al. Postural change in blood pressure associated with age and systolic blood pressure. The National Health and Nutrition Examination Survey II. *Journals of Gerontology* 1991;46(5):1991.
22. Wu JS, Yang YC, Lu FH, et al. Population-based study on the prevalence and risk factors of orthostatic hypotension in subjects with pre-diabetes and diabetes. *Diabetes Care* 2009;32(1):69-74. doi: http://dx.doi.org/10.2337/dc08-1389
23. Applegate WB, Davis BR, Black HR, et al. Prevalence of postural hypotension at baseline in the systolic hypertension in the elderly program (SHEP) cohort. *Journal of the American Geriatrics Society* 1991;39(11):1991.
24. Kwok CS, Ong ACL, Potter JF, et al. TIA, stroke and orthostatic hypotension: A disease spectrum related to ageing vasculature? *International Journal of Clinical Practice* 2014;68(6):June. doi: http://dx.doi.org/10.1111/ijcp.12373
25. Rutan GH, Hermanson B, Bild DE, et al. Orthostatic hypotension in older adults. The Cardiovascular Health Study. CHS Collaborative Research Group. *Hypertension* 1992;19(6:Pt 1):t-19.
26. Lin ZQ, Pan CM, Li WH, et al. [The correlation between postural hypotension and myocardial infarction in the elderly population]. [Chinese]. *Zhonghua nei ke za zhi [Chinese journal of internal medicine]* 2012;51(7):Jul.
27. Gupta A, Gilden J. Prevalence of diabetes in patients admitted to the hospital with primary diagnosis of orthostatic hypotension. *Diabetes Conference: 73rd Scientific Sessions of the American Diabetes Association Chicago, IL United States Conference Start: 20130621 Conference End: 20130625 Conference Publication: (var pagings)* 2013;62(pp A148):July. doi: http://dx.doi.org/10.2337/db13-388-679
28. Mancia G, Fagard R, Narkiewicz K, et al. 2013 ESH/ESC Guidelines for the management of arterial hypertension. *J Hypertens* 2013;31:1281-357.
29. Mejzner N, Clark CE, Smith LF, et al. Trends in the diagnosis and management of hypertension: repeated primary care survey in South West England. *British Journal of General Practice* 2017 doi: 10.3399/bjgp17X690461
30. Hale WA, Chambliss ML. Should primary care patients be screened for orthostatic hypotension? *The Journal of family practice* 1999;48(7):Jul.
31. Hobbs FD, Bankhead C, Mukhtar T, et al. Clinical workload in UK primary care: a retrospective analysis of 100 million consultations in England, 2007-14. *Lancet* 2016;387(10035):2323-30. doi: 10.1016/s0140-6736(16)00620-6 [published Online First: 2016/04/10]
32. Clegg A, Bates C, Young J, et al. Development and validation of an electronic frailty index using routine primary care electronic health record data. *Age and Ageing* 2016;45(3):353-60. doi: 10.1093/ageing/afw039
33. O'Connell MDL, Savva GM, Fan CW, et al. Orthostatic hypotension, orthostatic intolerance and frailty: The Irish Longitudinal Study on Aging-TILDA. *Archives of Gerontology and Geriatrics* 2015;60(3):507-13. doi: http://dx.doi.org/10.1016/j.archger.2015.01.008
34. Collins GS, Reitsma JB, Altman DG, et al. Transparent reporting of a multivariable prediction model for individual prognosis or diagnosis (TRIPOD): the TRIPOD statement. *BMJ : British Medical Journal* 2015;350 doi: 10.1136/bmj.g7594
35. Ferrucci L, Bandinelli S, Benvenuti E, et al. Subsystems contributing to the decline in ability to walk: bridging the gap between epidemiology and geriatric practice in the InCHIANTI study. *J AmGeriatrSoc* 2000;48(12):1618-25.

For peer review only - http://bmjopen.bmj.com/site/about/guidelines.xhtml

36. Daskalopoulou SS, Rabi DM, Zarnke KB, et al. The 2015 Canadian Hypertension Education Program recommendations for blood pressure measurement, diagnosis, assessment of risk, prevention, and treatment of hypertension. *Can J Cardiol* 2015;31(5):549-68. doi: 10.1016/j.cjca.2015.02.016
37. Steyerberg EW. Clinical prediction models : a practical approach to development, validation, and updating. New York ; London: Springer 2009.
38. Collett D. Modelling survival data in medical research. Boca Raton, Fla: Chapman & Hall/CRC 2003.
39. Cooke J, Carew S, O'Connor M, et al. Sitting and standing blood pressure measurements are not accurate for the diagnosis of orthostatic hypotension. *QJM: An International Journal of Medicine* 2009;102(5):335-39. doi: 10.1093/qjmed/hcp020
40. Tilvis RS, Hakala S-M, Valvanne J, et al. Postural hypotension and dizziness in a general aged population: A four- year follow-up of the Helsinki Aging Study. *Journal of the American Geriatrics Society* 1996;44(7):July.
41. Rockwood MR, Howlett SE, Rockwood K. Orthostatic hypotension (OH) and mortality in relation to age, blood pressure and frailty. *Archives of Gerontology & Geriatrics* 2012;54(3):e255-e60. doi: http://dx.doi.org/10.1016/j.archger.2011.12.009
42. O'Connell MDL, Savva GM, Fan CW, et al. Orthostatic hypotension, orthostatic intolerance and frailty: The Irish Longitudinal Study on Aging-TILDA. *Archives of Gerontology and Geriatrics* 2015;60(3):01. doi: http://dx.doi.org/10.1016/j.archger.2015.01.008
43. Phipps MS, Schmid AA, Kapoor JR, et al. Orthostatic hypotension among outpatients with ischemic stroke. *Journal of the Neurological Sciences* 2012;314(1-2):15. doi: http://dx.doi.org/10.1016/j.jns.2011.10.031
44. Eguchi K, Kario K, Hoshide S, et al. Greater change of orthostatic blood pressure is related to silent cerebral infarct and cardiac overload in hypertensive subjects. *Hypertension Research* 2004;27(4):April. doi: http://dx.doi.org/10.1291/hypres.27.235
45. Kario K, Shimada K. Orthostatic blood pressure changes and silent cerebrovascular disease. *Cardiology Review* 2003;20(4):01.
46. Falaschetti E, Mindell J, Knott C, et al. Hypertension management in England: a serial cross-sectional study from 1994 to 2011. *Lancet* 2014;383(9932):1912-19.
47. Rascol O, Perez-Lloret S, Damier P, et al. Falls in ambulatory non-demented patients with Parkinson's disease. *Journal of Neural Transmission* 2015;122(10):07. doi: http://dx.doi.org/10.1007/s00702-015-1396-2
48. Senard JM, Rai S, Lapeyre-Mestre M, et al. Prevalence of orthostatic hypotension in Parkinson's disease. *Journal of Neurology Neurosurgery and Psychiatry* 1997;63(5):November.
49. Palma J-A, Gomez-Esteban JC, Norcliffe-Kaufmann L, et al. Orthostatic Hypotension in Parkinson Disease: How Much You Fall or How Low You Go? *Movement Disorders* 2015;30(5):15. doi: http://dx.doi.org/10.1002/mds.26079
50. Schwartz CL, Clark C, Koshiaris C, et al. Interarm Difference in Systolic Blood Pressure in Different Ethnic Groups and Relationship to the "White Coat Effect": A Cross-Sectional Study. *Am J Hypertens* 2017 doi: 10.1093/ajh/hpx073
51. Kamaruzzaman S, Watt H, Carson C, et al. The association between orthostatic hypotension and medication use in the British Women's Heart and Health Study. *Age and Ageing* 2010;39(1):51-56. doi: http://dx.doi.org/10.1093/ageing/afp192
52. Clark CE. The interarm blood pressure difference: Do we know enough yet? *The Journal of Clinical Hypertension* 2017;19(5):462-65. doi: 10.1111/jch.12982
53. Liu K, Wang S, Wan S, et al. Arterial Stiffness, Central Pulsatile Hemodynamic Load, and Orthostatic Hypotension. *Journal of Clinical Hypertension* 2016;18(7):655-62. doi: http://dx.doi.org/10.1111/jch.12726

For peer review only - http://bmjopen.bmj.com/site/about/guidelines.xhtml

54. Meng Q, Wang S, Wang Y, et al. Arterial stiffness is a potential mechanism and promising indicator of orthostatic hypotension in the general population. *Vasa - European Journal of Vascular Medicine* 2014;43(6):423-32. doi: http://dx.doi.org/10.1024/0301-1526/a000389
55. Scuteri A, Tesauro M, Guglini L, et al. Aortic stiffness and hypotension episodes are associated with impaired cognitive function in older subjects with subjective complaints of memory loss. *Int J Cardiol* 2013;169(5):371-7. doi: 10.1016/j.ijcard.2013.09.009 [published Online First: 2013/10/15]
56. Eze CO, Onwuekwe IO, Agu CE, et al. The prevalence of orthostatic hypotension in type 2 diabetes mellitus patients in a diabetic clinic in Enugu South-East Nigeria. *Nigerian journal of medicine : journal of the National Association of Resident Doctors of Nigeria* 2013;22(3):2013-2Sep.
57. Lanthier L, Touchette M, Bourget P, et al. [Evaluation of circadian variation of blood pressure by ambulatory blood pressure monitoring in an elderly diabetic population with or without orthostatic hypotension]. [French]. *Geriatrie et psychologie neuropsychiatrie du vieillissement* 2011;9(1):Mar.
58. Rota E, Quadri R, Fanti E, et al. Clinical and electrophysiological correlations in type 2 diabetes mellitus at diagnosis. *Diabetes Research and Clinical Practice* 2007;76(1):April. doi: http://dx.doi.org/10.1016/j.diabres.2006.07.027
59. Kirkman MS, Briscoe VJ, Clark N, et al. Diabetes in Older Adults. *Diabetes Care* 2012;35(12):2650-64. doi: 10.2337/dc12-1801
60. Elmstahl S, Widerstrom E. Orthostatic intolerance predicts mild cognitive impairment: incidence of mild cognitive impairment and dementia from the Swedish general population cohort Good Aging in Skane. *Clinical interventions in aging* 2014;9(pp 1993-2002):2014. doi: http://dx.doi.org/10.2147/CIA.S72316
61. Frewen J, Savva GM, Boyle G, et al. Cognitive performance in orthostatic hypotension: Findings from a nationally representative sample. *Journal of the American Geriatrics Society* 2014;62(1):January. doi: http://dx.doi.org/10.1111/jgs.12592
62. Frewen J, Finucane C, Savva GM, et al. Orthostatic hypotension is associated with lower cognitive performance in adults aged 50 plus with supine hypertension. *The journals of gerontology Series A, Biological sciences and medical sciences* 2014;69(7):Jul. doi: http://dx.doi.org/10.1093/gerona/glt171
63. Viramo P, Luukinen H, Koski K, et al. Orthostatic hypotension and cognitive decline in older people. *Journal of the American Geriatrics Society* 1999;47(5):May.
64. Wolters FJ, Mattace-Raso FUS, Koudstaal PJ, et al. Orthostatic Hypotension and the Long-Term Risk of Dementia: A Population-Based Study. *PLoS Medicine* 2016;13 (10) (no pagination)(e1002143) doi: http://dx.doi.org/10.1371/journal.pmed.1002143
65. Vloet LCM, Smits R, Frederiks CMA, et al. Evaluation of skills and knowledge on orthostatic blood pressure measurements in elderly patients. *Age and Ageing* 2002;31(3):2002. doi: http://dx.doi.org/10.1093/ageing/31.3.211
66. Netea RT, Elving LD, Lutterman JA, et al. Body position and blood pressure measurement in patients with diabetes mellitus. *Journal of Internal Medicine* 2002;251(5):2002. doi: http://dx.doi.org/10.1046/j.1365-2796.2002.00958.x
67. Mo R, Omvik P, Lund-Johansen P. The Bergen Blood Pressure Study. Estimated prevalence of postural hypotension is influenced by the alerting reaction to blood pressure measurement. *Journal of Human Hypertension* 1994;8(3):1994.
68. Hale WA, Chambliss ML. Should primary care patients be screened for orthostatic hypotension? *The Journal of family practice* 1999;48(7):547-52.
69. Ruwald MH, Hansen ML, Lamberts M, et al. Comparison of incidence, predictors, and the impact of co-morbidity and polypharmacy on the risk of recurrent syncope in patients <85 versus >85 years of age. *American Journal of Cardiology* 2013;112(10):15. doi: http://dx.doi.org/10.1016/j.amjcard.2013.07.041

For peer review only - http://bmjopen.bmj.com/site/about/guidelines.xhtml

70. Pepersack T, Gilles C, Petrovic M, et al. Prevalence of orthostatic hypotension and relationship with drug use amongst older patients. *Acta clinica Belgica* 2013;68(2):2013-2Apr.
71. Poon IO, Braun U. High prevalence of orthostatic hypotension and its correlation with potentially causative medications among elderly veterans. *Journal of Clinical Pharmacy and Therapeutics* 2005;30(2):April. doi: http://dx.doi.org/10.1111/j.1365-2710.2005.00629.x
72. Kobayashi K, Yamada S. Development of a simple index, calf mass index, for screening for orthostatic hypotension in community-dwelling elderly. *Archives of Gerontology and Geriatrics* 2012;54(2):March. doi: http://dx.doi.org/10.1016/j.archger.2011.04.003
73. Di SC, Milazzo V, Bruno G, et al. Prevalence of orthostatic hypotension in a cohort of patients under antihypertensive therapy. *High Blood Pressure and Cardiovascular Prevention Conference: 2013 National Congress of the Italian Society of Hypertension, SIIA 2013 Rome Italy Conference Start: 20131003 Conference End: 20131005 Conference Publication: (var pagings)* 2013;20(3):September. doi: http://dx.doi.org/10.1007/s40292-013-0021-4
74. Kamaruzzaman S, Watt H, Carson C, et al. The association between orthostatic hypotension and medication use in the British Women's Heart and Health Study. *Age and Ageing* 2010;39(1):afp192.
75. Barochiner J, Alfie J, Aparicio LS, et al. Prevalence and clinical profile of resistant hypertension among treated hypertensive subjects. *Clinical and Experimental Hypertension* 2013;35(6):2013. doi: http://dx.doi.org/10.3109/10641963.2012.739236
76. Bouhanick B, Meliani S, Doucet J, et al. Orthostatic hypotension is associated with more severe hypertension in elderly autonomous diabetic patients from the French Gerodiab study at inclusion. *Annales de Cardiologie et d Angeiologie* 2014;63(3):176-82. doi: http://dx.doi.org/10.1016/j.ancard.2014.05.013
77. Auseon A, Ooi WL, Hossain M, et al. Blood pressure behavior in the nursing home: Implications for diagnosis and treatment of hypertension. *Journal of the American Geriatrics Society* 1999;47(3):March.
78. Masuo K, Mikami H, Ogihara T, et al. Changes in frequency of orthostatic hypotension in elderly hypertensive patients under medications. *American Journal of Hypertension* 1996;9(3):March. doi: http://dx.doi.org/10.1016/0895-7061%2895%2900348-7
79. Fotherby MD, Potter JF. Orthostatic hypotension and anti-hypertensive therapy in the elderly. *Postgraduate Medical Journal* 1994;70(830):1994.
80. Fotherby MD, Robinson TG, Potter JF. Clinic and 24 h blood pressure in elderly treated hypertensives with postural hypotension. *Journal of Human Hypertension* 1994;8(9):1994.
81. Parker E, Glasziou P. Use of evidence in hypertension guidelines: should we measure in both arms? *British Journal of General Practice* 2009;59:e87-e92. doi: 10.3399/bjgp09X395012

For peer review only

For peer review only - http://bmjopen.bmj.com/site/about/guidelines.xhtml

Authors' contributions

CEC conceived and undertook this analysis. DT contributed to the analysis. FW contributed to the analysis and offered statistical advice and support. DL offered advice on analysis and interpretation of cognitive impairment indices. LF supported the study on behalf of the InCHIANTI investigators. JLC supervised study conduct. CEC drafted the manuscript, all authors revised and edited the manuscript and all authors have read, reviewed, and approved the final manuscript.

Funding statement

CEC is supported by an NIHR Clinical Lectureship award. The views expressed are those of the authors and not necessarily those of the NIHR, the NHS or the Department of Health. This work was supported in part by the Intramural Research Program of the National Institute on Aging, National Institute of Health, Baltimore MD21224, USA.

Competing interests statement

All authors assert that they have no competing interests to declare

Previous dissemination

Interim reports on this work have been presented at annual scientific meetings of the European Society for Hypertension, Paris 2016 (Clark C, Thomas D, Warren F et al. Predicting postural hypotension, falls, and cognitive impairment: the InCHIANTI study. *J Hypertens* 2016; 34, e-Supplement 2: e32, September 2016) and the British and Irish Hypertension Society, Dublin, 2016 (Clark C, Thomas D, Mejzner N, et al. Can we predict who should be tested for postural hypotension? Derivation and validation of a prediction tool. *Journal of Human Hypertension* 2016;30 doi: doi:10.1038/jhh.2016.60)

Data sharing statement

The InCHIANTI datasets are available on application with a research proposal to the InCHIANTI investigators at http://inchiantistudy.net/wp/

For peer review only

For peer review only - http://bmjopen.bmj.com/site/about/guidelines.xhtml

Legends for tables and figures

Table 1. Risk markers included in univariable analysis

Table 2. Baseline characteristics of derivation and validation cohorts

Table 3. Univariable associations of risk markers with postural hypotension in derivation cohort

Table 4. Multivariable prediction models for postural hypotension

Table 5. DROP score associations with postural hypotension, mortality and cognitive decline

Figure 1. Prevalence of postural hypotension vs unweighted DROP Score without inter-arm difference term (Population prevalence indicated by horizontal line)

Figure 2. Kaplan Meier survival plot for DROP scores over 9 years follow up

Figure 3. Mean change in Mini Mental State Examination score over nine years per DROP score

For peer review only

For peer review only - http://bmjopen.bmj.com/site/about/guidelines.xhtml

Group	Risk marker included in analysis
Demographics	age<br>gender
Medical History	hypertension<br>heart failure<br>myocardial infarction<br>angina<br>stroke<br>diabetes<br>Parkinson's disease<br>cancer<br>dementia
Examination	Mini Mental State Examination
Medications	antihypertensives<br>antiarrhythmics<br>antidepressants<br>antipsychotics<br>anxiolytics<br>anticholinesterase inhibitors
Frailty	hospital admission, fall, or weight loss in last 12 months<br>WHO physical disability level<br>ADL disability score

Table 1. Risk markers included in univariable analysis

For peer review only

	Derivation Cohort	Validation Cohort	p
**N**	**649**	**668**	
	**Mean (SD) or N/%**	**Mean (SD) or N/%**	**t/ χ^2**
**age**	68.5 (15.7)	68.2 (15.3)	0.77
**BMI**	27.2 (4.3)	27.1 (4.0)	0.59
**Supine SBP (higher arm)#**	145.9 (21.3)	146.3 (21.6)	0.76
**Supine DBP (higher arm)#**	82.9 (8.8)	83.1 (9.5)	0.59
**Standing SBP 1 min**	140.4 (21.0)	141.2 (21.3)	0.51
**Standing DBP 1 min**	83.0 (8.9)	83.6 (9.4)	0.25
**Standing SBP 3 min**	141.4 (20.9)	141.9 (20.9)	0.66
**Standing DBP 3 min**	82.7 (9.0)	83.0 (9.4)	0.60
**Female**	368 (56.7)	358 (53.6)	0.27
**Site (Greve vs Bagno a Ripoli)**	320 vs 329	327 vs 341	0.91
**Deceased @ 9 years**	199 (30.7)	203 (30.4)	0.95
**Systolic drop ≥20mmHg 1min**	56 (8.6)	45 (6.7)	0.21
**Diastolic drop ≥10mmHg 1min**	41 (6.3)	40 (6.0)	0.82
**Systolic drop ≥20mmHg 3 min**	47 (7.2)	42 (6.3)	0.51
**Diastolic drop ≥10mmHg 3min**	46 (7.1)	48 (7.2)	1.00
**Postural Hypotension present***	107 (16.5)	96 (14.4)	0.32
**Systolic inter-arm difference ≥10mmHg**	121 (18.8)	121 (18.1)	0.83
**Previous stroke**	44 (6.8)	45 (6.7)	1.00
**Pre-existing diabetes**	80 (12.3)	76 (11.4)	0.61
**Pre-existing hypertension**	279 (43.0)	292 (43.7)	0.82
**Pre-existing CV disease**	63 (9.7)	50 (7.5)	0.17
**Pre-existing dementia**	38 (5.9)	27 (4.0)	0.16
**Pre-existing Parkinson's disease**	9 (1.4)	6 (0.9)	0.45
**Fall in preceding 12 months**	143 (22.0)	130 (19.5)	0.28

#mean of 2nd and 3rd readings
*defined as a drop of ≥20mmHg systolic or ≥10mmHg diastolic within 3 minutes of standing

Table 2. Baseline characteristics of derivation and validation cohorts

For peer review only

**Variable (n (%) unless otherwise stated)**	**PH absent (n=542)**	**PH present (n=107)**	**p value**
Age (mean, SD)	67.7 (15.8)	72.2 (14.6)	0.005
Age over 60	438 (81)	96 (90)	0.027
Age over 65	421 (78)	90 (84)	0.160
Age over 70	302 (56)	73 (68)	0.018
MMSE score (mean, SD)	25.3 (4.9)	24.1 (5.1)	0.031
Female gender	301 (55.5)	67 (62.6)	0.200
Angiotensin converting enzyme inhibitors	103 (19)	23 (22)	0.552
Angiotensin-2 antagonists	6 (1)	4 (4)	0.066
Calcium channel blockers	62 (11)	15 (14)	0.451
Diuretics	48 (9)	17 (16)	0.027
Beta-blockers	20 (4)	4 (4)	0.981
alpha-blockers	11 (2)	1 (1)	0.442
aldosterone antagonists	2 (0.4)	0 (0)	0.529
Digoxin	27 (5)	14 (13)	0.004
Antiarrhythmics, class I and III	10 (2)	4 (4)	0.264
Psycholeptics: typical antipsychotics	8 (1)	4 (4)	0.119
Psycholeptics: atypical antipsychotics	6 (1)	1 (1)	1.000
Psycholeptics: anxiolytics	103 (19)	18 (17)	0.684
Psychoanaleptics: antidepressants	22 (4)	5 (5)	0.791
Drugs for dementia	5 (1)	0 (0)	1.000
Hypertension	217 (40)	62 (58)	0.001
Congestive heart failure	22 (4)	10 (9)	0.028
Myocardial infarction	23 (4)	6 (6)	0.607
Angina	21 (4)	7 (6)	0.421
Any CV disease	45 (8)	18 (17)	0.011
Stroke	28 (5)	16 (15)	0.001
Diabetes	64 (12)	16 (15)	0.420
Parkinson's disease	4 (1)	5 (5)	0.008
Any cancer	30 (6)	8 (8)	0.497
Dementia	29 (5)	9 (8)	0.257
MMSE score 22 to 26	150 (28)	27 (25)	0.637
hospital admission in past year	54 (10)	18 (17)	0.044
Weight loss ≥10lbs in past year	22 (4)	7 (6)	0.301
Any fall in past year	115 (21)	28 (26)	0.254
Any ADL disability	100 (19)	28 (26)	0.083
WHO disability level >1	66 (12)	24 (23)	0.045
Systolic blood pressure (mean, SD) mmHg	144.3 (20.1)	153.7 (25.3)	<0.001
Diastolic blood pressure (mean, SD) mmHg	82.2 (8.8)	86.2 (8.1)	<0.001

For peer review only - http://bmjopen.bmj.com/site/about/guidelines.xhtml

Systolic inter-arm difference (mean, SD) mmHg	2.0 (4.1)	4.7 (5.9)	<0.001
Systolic inter-arm BP difference ≥10mmHg	81 (15)	40 (37)	<0.001
Systolic inter-arm BP difference ≥ 15mmHg	10 (2)	6 (6)	0.007

p values derived from t-tests for continuous data, or Pearson chi-square for categorical data; Fisher's exact test reported where expected cell count <5

Table 3. Univariable associations of risk markers with postural hypotension in derivation cohort

For peer review only

For peer review only - http://bmjopen.bmj.com/site/about/guidelines.xhtml

**Variable**	**Odds Ratio**	**95% Confidence Interval**
Model 1		
Parkinson's disease	4.7	1.2 to 19.2
Previous stroke	2.2	1.1 to 4.5
Taking digoxin	2.2	1.0 to 4.7
Previous cardiac disease	1.9	1.0 to 3.6
Hypertension	1.7	1.1 to 2.6
Systolic inter-arm difference (continuous per mmHg)	1.1	1.1 to 1.2
Model 2		
Parkinson's disease	5.0	1.2 to 19.9
Previous stroke	2.2	1.1 to 4.4
Taking digoxin	2.4	1.1 to 5.1
Previous cardiac disease	1.9	1.0 to 2.6
Hypertension	1.7	1.1 to 5.1
Systolic inter-arm difference ≥10mmHg	3.3	2.0 to 5.3
Model 3		
Parkinson's disease	5.3	1.4 to 20.4
Previous stroke	2.4	1.2 to 4.8
Taking digoxin	2.0	0.9 to 4.3
Previous cardiac disease	1.8	0.9 to 3.4
Hypertension	1.9	1.3 to 3.0

Table 4. Multivariable prediction models for postural hypotension

For peer review only

	Including inter-arm difference		Excluding inter-arm difference	
	**Weighted**	**Unweighted**	**Weighted**	**Unweighted**
Prediction of PH per unit increase of DROP score OR (95%CI)	1.9 (1.4 to 2.5)	1.8 (1.4 to 2.3)	2.4 (1.6 to 3.4)	2.0 (1.5 to 2.6)
Area under ROC curve (95%CI)	0.65 (0.59 to 0.70)	0.65 (0.60 to 0.71)	0.65 (0.59 to 0.71)	0.65 (0.59 to 0.70)
Mortality risk per unit score OR (95%CI)	1.9 (1.6 to 2.2)	1.8 (1.5 to 2.1)	2.8 (2.2 to 3.4)	2.1 (1.8 to 2.5)
Change in MMSE score over study (ANOVA)	N/A	P=0.004	N/A	P<0.001
Annual change in MMSE score (ANOVA)	N/A	P<0.001	N/A	P<0.001

Table 5. DROP score associations with postural hypotension, mortality and cognitive decline

For peer review only

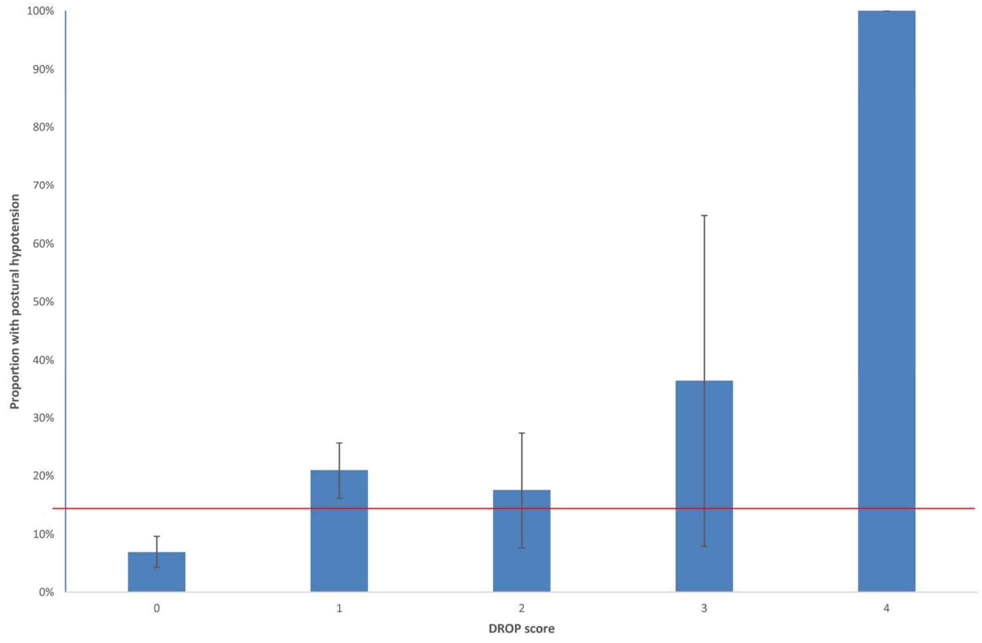

Prevalence of postural hypotension vs unweighted DROP Score without inter-arm difference term (Population prevalence indicated by horizontal line)

167x108mm (300 x 300 DPI)

view only

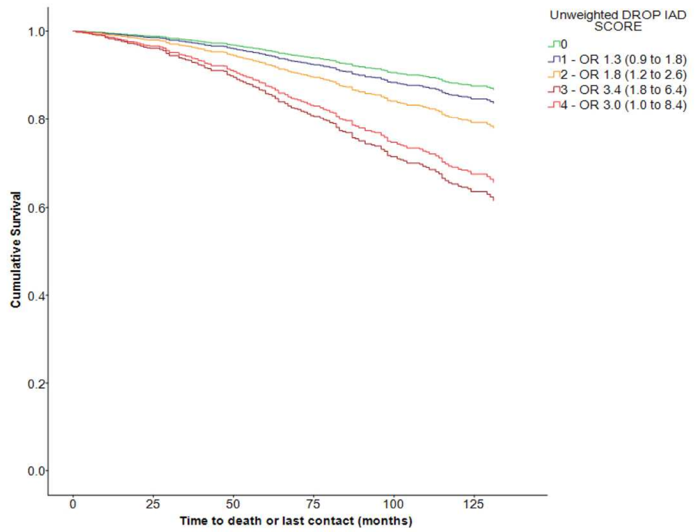

Kaplan Meier survival plot for DROP scores over 9 years follow up

209x148mm (300 x 300 DPI)

For peer review only - http://bmjopen.bmj.com/site/about/guidelines.xhtml

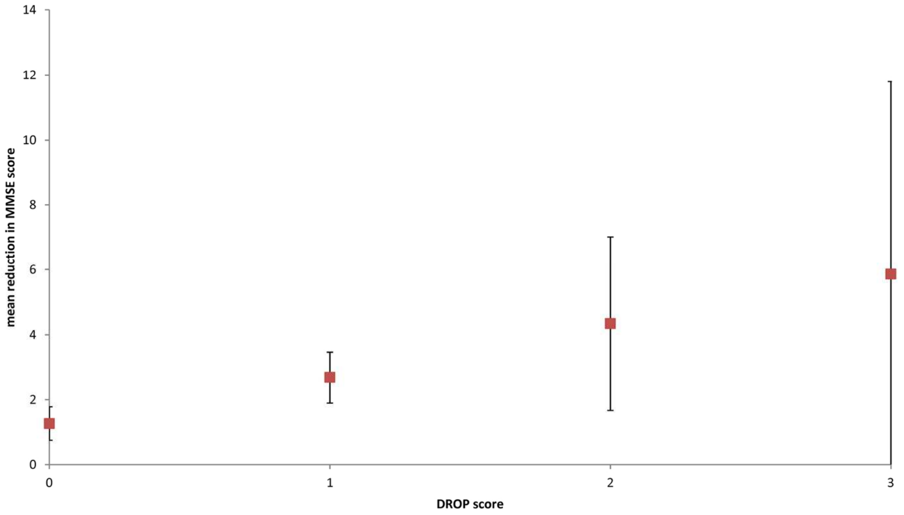

Mean change in Mini Mental State Examination score over nine years per DROP score

165x108mm (300 x 300 DPI)

view only

Appendix: Literature search for factors associated with postural hypotension

Demographics:
- Increasing age[1-9]
- Female gender[10]
- Nursing home residence[11-15]

Medical History:
- Hypertension[7-10 16-20] and uncontrolled hypertension[6 21 22]
- Diabetes and diabetic complications[17 23-28]
- Chronic Kidney Disease[10 29 30]
- Stroke[31-36]
- Ischaemic heart disease[36 37]
- Heart failure[38 39]
- Parkinson's disease[40-42]
- Cognitive impairment[43-50]
- Depression[51]

Medications:
- Antiarrhythmic drugs[11]
- Antihypertensives[4 9-11 52-55] (negative association with ACE inhibitors)[10]
- Psychotropic agents (antipsychotics, sedatives, antidepressants)[23 53 56]
- Anticholinesterase inhibitors[50]

Biochemical:
- Vitamin D deficiency (conflicting evidence)[1 23 57 58]

Frailty:[59 60]
- Falls[61]
- Get up and go test[11]
- Reduced calf mass index[54 62]
- Activity of Daily Living disability score[1 11]
- Cumulative illness Rating Scale for Geriatrics score[23]

Environmental:
- Seasons – prevalence higher in summer and in heatwaves[63 64]
- Time of day – higher in mornings[65-68]

For peer review only - http://bmjopen.bmj.com/site/about/guidelines.xhtml

References

1. Soysal P, Yay A, Isik AT. Does vitamin D deficiency increase orthostatic hypotension risk in the elderly patients? *Archives of Gerontology and Geriatrics* 2014;59(1):July. doi: http://dx.doi.org/10.1016/j.archger.2014.03.008

2. Moret F, Jaccard-Ruedin H, Bula C, et al. The high diagnostic yield of an outpatient geriatric clinic. *Journal of the American Geriatrics Society Conference: 2012 Annual Scientific Meeting of the American Geriatrics Society Seattle, WA United States Conference Start: 20120503 Conference End: 20120505 Conference Publication: (var pagings)* 2012;60(pp S175):April. doi: http://dx.doi.org/10.1111/j.1532-5415.2012.04000.x

3. Robertson D, Desjardin JA, Lichtenstein MJ. Distribution and observed associations of orthostatic blood pressure changes in elderly general medicine outpatients. *American Journal of the Medical Sciences* 1998;315(5):1998. doi: http://dx.doi.org/10.1097/00000441-199805000-00001

4. Poon IO, Braun U. High prevalence of orthostatic hypotension and its correlation with potentially causative medications among elderly veterans. *Journal of Clinical Pharmacy and Therapeutics* 2005;30(2):April. doi: http://dx.doi.org/10.1111/j.1365-2710.2005.00629.x

5. Mendez CA, Melgarejo JD, Lee JH, et al. Orthostatic hypotension in latino elderly: Findings from the maracaibo ageing study. *Journal of Hypertension Conference: 25th European Meeting on Hypertension and Cardiovascular Protection, ESH 2015 Milan Italy Conference Start: 20150612 Conference End: 20150615 Conference Publication: (var pagings)* 2015;33(pp e219):June. doi: http://dx.doi.org/10.1097/01.hjh.0000468017.03671.63

6. Barochiner J, Alfie J, Aparicio L, et al. Orthostatic hypotension in treated hypertensive patients. *Romanian journal of internal medicine = Revue roumaine de medecine interne* 2012;50(3):2012-2Sep.

7. Shin C, Abbott RD, Lee H, et al. Prevalence and correlates of orthostatic hypotension in middle-aged men and women in Korea: The Korean Health and Genome Study. *Journal of Human Hypertension* 2004;18(10):October. doi: http://dx.doi.org/10.1038/sj.jhh.1001732

8. Harris T, Lipsitz LA, Kleinman JC, et al. Postural change in blood pressure associated with age and systolic blood pressure. The National Health and Nutrition Examination Survey II. *Journals of Gerontology* 1991;46(5):1991.

9. Kamaruzzaman S, Watt H, Carson C, et al. The association between orthostatic hypotension and medication use in the British Women's Heart and Health Study. *Age and Ageing* 2010;39(1):afp192. doi: http://dx.doi.org/10.1093/ageing/afp192

10. Fedorowski A, Burri P, Melander O. Orthostatic hypotension in genetically related hypertensive and normotensive individuals. *Journal of Hypertension* 2009;27(5):May. doi: http://dx.doi.org/10.1097/HJH.0b013e3283279860

11. Boland E, Brackelaire G, Anani Y, et al. Postprandial hypotension among patients from an acute geriatric ward: Results from a pilot study. *European Geriatric Medicine Conference: 11th International Congress of the European Union Geriatric Medicine Society, EUGMS 2015 Oslo Norway Conference Start: 20150916 Conference End: 20150918 Conference Publication: (var pagings)* 2015;6(pp S64):September.

12. Hartog LC, Cizmar-Sweelssen M, Knipscheer A, et al. The association between orthostatic hypotension, falling and successful rehabilitation in a nursing home population. *Archives of Gerontology and Geriatrics* 2015;61(2):01. doi: http://dx.doi.org/10.1016/j.archger.2015.05.005

13. Asensio LE, Aguilera AAC, Corral MACC, et al. Prevalence of orthostatic hypotension in a series of elderly institutionalized patients. *Europace Conference: EHRA Europace 2011 Madrid Spain Conference Start: 20110626 Conference End: 20110629 Conference Publication: (var pagings)* 2011;13 doi: http://dx.doi.org/10.1093/europace/eur231

For peer review only - http://bmjopen.bmj.com/site/about/guidelines.xhtml

14. Matusik P, Nowak J, Tomaszewski K, et al. Hypertension among the elderly on the basis of nursing home residents population. [Polish]. *Polski Przeglad Kardiologiczny* 2010;12(3):2010.
15. Wu J-S, Yang Y-C, Lu F-H, et al. Population-based study on the prevalence and correlates of orthostatic hypotension/hypertension and orthostatic dizziness. *Hypertension Research* 2008;31(5):May. doi: http://dx.doi.org/10.1291/hypres.31.897
16. Saez T, Suarez C, Sierra MJ, et al. Orthostatic hypotension in the elderly and its association with the antihypertensive treatment. [Spanish]. *Medicina Clinica* 2000;114(14):15.
17. Wu JS, Yang YC, Lu FH, et al. Population-based study on the prevalence and risk factors of orthostatic hypotension in subjects with pre-diabetes and diabetes. *Diabetes Care* 2009;32(1):69-74. doi: http://dx.doi.org/10.2337/dc08-1389
18. Fan X-H, Sun K, Zhou X-L, et al. Association of orthostatic hypertension and hypotension with target organ damage in middle and old-aged hypertensive patients. [Chinese]. *National Medical Journal of China* 2011;91(4):04. doi: http://dx.doi.org/10.3760/cma.j.issn.0376-2491.2011.04.002
19. Barochiner J, Alfie J, Aparicio LS, et al. Prevalence and clinical profile of resistant hypertension among treated hypertensive subjects. *Clinical and Experimental Hypertension* 2013;35(6):2013. doi: http://dx.doi.org/10.3109/10641963.2012.739236
20. Bouhanick B, Meliani S, Doucet J, et al. Orthostatic hypotension is associated with more severe hypertension in elderly autonomous diabetic patients from the French Gerodiab study at inclusion. *Annales de Cardiologie et d Angeiologie* 2014;63(3):176-82. doi: http://dx.doi.org/10.1016/j.ancard.2014.05.013
21. Auseon A, Ooi WL, Hossain M, et al. Blood pressure behavior in the nursing home: Implications for diagnosis and treatment of hypertension. *Journal of the American Geriatrics Society* 1999;47(3):March.
22. Masuo K, Mikami H, Ogihara T, et al. Changes in frequency of orthostatic hypotension in elderly hypertensive patients under medications. *American Journal of Hypertension* 1996;9(3):March. doi: http://dx.doi.org/10.1016/0895-7061%2895%2900348-7
23. Paccalin M. Factors associated with orthostatic hypotension in hospitalized elderly patients. *European Geriatric Medicine Conference: 11th International Congress of the European Union Geriatric Medicine Society, EUGMS 2015 Oslo Norway Conference Start: 20150916 Conference End: 20150918 Conference Publication: (var pagings)* 2015;6(pp S62):September.
24. van Hateren KJJ, Kleefstra N, Blanker MH, et al. Orthostatic hypotension, diabetes, and falling in older patients: A cross-sectional study. *British Journal of General Practice* 2012;62(603):October. doi: http://dx.doi.org/10.3399/bjgp12X656838
25. Gupta A, Gilden J. Prevalence of diabetes in patients admitted to the hospital with primary diagnosis of orthostatic hypotension. *Diabetes Conference: 73rd Scientific Sessions of the American Diabetes Association Chicago, IL United States Conference Start: 20130621 Conference End: 20130625 Conference Publication: (var pagings)* 2013;62(pp A148):July. doi: http://dx.doi.org/10.2337/db13-388-679
26. Eze CO, Onwuekwe IO, Agu CE, et al. The prevalence of orthostatic hypotension in type 2 diabetes mellitus patients in a diabetic clinic in Enugu South-East Nigeria. *Nigerian journal of medicine : journal of the National Association of Resident Doctors of Nigeria* 2013;22(3):2013-2Sep.
27. Lanthier L, Touchette M, Bourget P, et al. [Evaluation of circadian variation of blood pressure by ambulatory blood pressure monitoring in an elderly diabetic population with or without orthostatic hypotension]. [French]. *Geriatrie et psychologie neuropsychiatrie du vieillissement* 2011;9(1):Mar.
28. Rota E, Quadri R, Fanti E, et al. Clinical and electrophysiological correlations in type 2 diabetes mellitus at diagnosis. *Diabetes Research and Clinical Practice* 2007;76(1):April. doi: http://dx.doi.org/10.1016/j.diabres.2006.07.027

For peer review only - http://bmjopen.bmj.com/site/about/guidelines.xhtml

29. Aghera D, McFadden C, Hunter K. Orthostatic hypotension in elderly individuals with chronic kidney disease(CKD). *American Journal of Kidney Diseases Conference: National Kidney Foundation 2015 Spring Clinical Meetings, NKF SCM15 Dallas, TX United States Conference Start: 20150325 Conference End: 20150329 Conference Publication: (var pagings)* 2015;65(4):April.
30. Bhat S, Hegde S, Szpunar S, et al. Prevalence of orthostatic variation in blood pressure among stable outpatient chronic kidney disease population. *American Journal of Kidney Diseases Conference: National Kidney Foundation 2013 Spring Clinical Meetings Orlando, FL United States Conference Start: 20130402 Conference End: 20130406 Conference Publication: (var pagings)* 2013;61(4):April. doi: http://dx.doi.org/10.1053/j.ajkd.2013.02.049
31. Phipps MS, Schmid AA, Kapoor JR, et al. Orthostatic hypotension among outpatients with ischemic stroke. *Journal of the Neurological Sciences* 2012;314(1-2):15. doi: http://dx.doi.org/10.1016/j.jns.2011.10.031
32. Ryan DJ, Kenny RA, Christensen S, et al. Ischaemic stroke or TIA in older subjects associated with impaired dynamic blood pressure control in the absence of severe large artery stenosis. *Age and Ageing* 2015;44(4):afv011. doi: http://dx.doi.org/10.1093/ageing/afv011
33. Eguchi K, Kario K, Hoshide S, et al. Greater change of orthostatic blood pressure is related to silent cerebral infarct and cardiac overload in hypertensive subjects. *Hypertension Research* 2004;27(4):April. doi: http://dx.doi.org/10.1291/hypres.27.235
34. Kario K, Shimada K. Orthostatic blood pressure changes and silent cerebrovascular disease. *Cardiology Review* 2003;20(4):01.
35. Kwok CS, Ong ACL, Potter JF, et al. TIA, stroke and orthostatic hypotension: A disease spectrum related to ageing vasculature? *International Journal of Clinical Practice* 2014;68(6):June. doi: http://dx.doi.org/10.1111/ijcp.12373
36. Rutan GH, Hermanson B, Bild DE, et al. Orthostatic hypotension in older adults. The Cardiovascular Health Study. CHS Collaborative Research Group. *Hypertension* 1992;19(6:Pt 1):t-19.
37. Lin ZQ, Pan CM, Li WH, et al. [The correlation between postural hypotension and myocardial infarction in the elderly population]. [Chinese]. *Zhonghua nei ke za zhi [Chinese journal of internal medicine]* 2012;51(7):Jul.
38. Potocka-Plazak K, Plazak W. Orthostatic hypotension in elderly women with congestive heart failure. *Aging - Clinical and Experimental Research* 2001;13(5):2001.
39. McGann PE. Comorbidity in heart failure in the elderly. *Clinics in Geriatric Medicine* 2000;16(3):2000.
40. Rascol O, Perez-Lloret S, Damier P, et al. Falls in ambulatory non-demented patients with Parkinson's disease. *Journal of Neural Transmission* 2015;122(10):07. doi: http://dx.doi.org/10.1007/s00702-015-1396-2
41. Senard JM, Rai S, Lapeyre-Mestre M, et al. Prevalence of orthostatic hypotension in Parkinson's disease. *Journal of Neurology Neurosurgery and Psychiatry* 1997;63(5):November.
42. Palma J-A, Gomez-Esteban JC, Norcliffe-Kaufmann L, et al. Orthostatic Hypotension in Parkinson Disease: How Much You Fall or How Low You Go? *Movement Disorders* 2015;30(5):15. doi: http://dx.doi.org/10.1002/mds.26079
43. Elmstahl S, Widerstrom E. Orthostatic intolerance predicts mild cognitive impairment: incidence of mild cognitive impairment and dementia from the Swedish general population cohort Good Aging in Skane. *Clinical interventions in aging* 2014;9(pp 1993-2002):2014. doi: http://dx.doi.org/10.2147/CIA.S72316
44. Frewen J, Savva GM, Boyle G, et al. Cognitive performance in orthostatic hypotension: Findings from a nationally representative sample. *Journal of the American Geriatrics Society* 2014;62(1):January. doi: http://dx.doi.org/10.1111/jgs.12592
45. Frewen J, Finucane C, Savva GM, et al. Orthostatic hypotension is associated with lower cognitive performance in adults aged 50 plus with supine hypertension. *The journals of gerontology*

For peer review only - http://bmjopen.bmj.com/site/about/guidelines.xhtml

Series A, Biological sciences and medical sciences 2014;69(7):Jul. doi: http://dx.doi.org/10.1093/gerona/glt171
46. Traykova M, Stankova T, Mehrabian S, et al. High prevalence of orthostatic hypotension in vascular and degenerative dementia. *European Journal of Medical Research Conference: 21st European Students' Conference Promising Medical Scientists Willing to Look Beyond Berlin Germany Conference Start: 20101013 Conference End: 20101017 Conference Publication: (var pagings)* 2010;15(pp 126-127):13.
47. Yap PL, Niti M, Yap KB, et al. Orthostatic hypotension, hypotension and cognitive status: early comorbid markers of primary dementia? *Dementia & Geriatric Cognitive Disorders* 2008;26(3):239-46. doi: http://dx.doi.org/10.1159/000160955
48. Sonnesyn H, Nilsen DW, Rongve A, et al. High prevalence of orthostatic hypotension in mild dementia. *Dementia and Geriatric Cognitive Disorders* 2009;28(4):November. doi: http://dx.doi.org/10.1159/000247586
49. Campbell AJ, Reinken J. Postural hypotension in old age: Prevalence, associations and prognosis. *Journal of Clinical and Experimental Gerontology* 1985;7(2):1985.
50. Isik AT, Soysal P, Mas M. Orthostatic hypotension and long-term effects of acheis on the orthostatic hypotension in elderly patients with alzheimer disease. *Alzheimer's and Dementia Conference: Alzheimer's Association International Conference 2014 Copenhagen Denmark Conference Start: 20140712 Conference End: 20140717 Conference Publication: (var pagings)* 2014;10(pp P774):July.
51. Regan CO, Kearney PM, Cronin H, et al. Oscillometric measure of blood pressure detects association between orthostatic hypotension and depression in population based study of older adults. *BMC psychiatry* 2013;13(pp 266):2013. doi: http://dx.doi.org/10.1186/1471-244X-13-266
52. Ruwald MH, Hansen ML, Lamberts M, et al. Comparison of incidence, predictors, and the impact of co-morbidity and polypharmacy on the risk of recurrent syncope in patients <85 versus >85 years of age. *American Journal of Cardiology* 2013;112(10):15. doi: http://dx.doi.org/10.1016/j.amjcard.2013.07.041
53. Pepersack T, Gilles C, Petrovic M, et al. Prevalence of orthostatic hypotension and relationship with drug use amongst older patients. *Acta clinica Belgica* 2013;68(2):2013-2Apr.
54. Kobayashi K, Yamada S. Development of a simple index, calf mass index, for screening for orthostatic hypotension in community-dwelling elderly. *Archives of Gerontology and Geriatrics* 2012;54(2):March. doi: http://dx.doi.org/10.1016/j.archger.2011.04.003
55. Di SC, Milazzo V, Bruno G, et al. Prevalence of orthostatic hypotension in a cohort of patients under antihypertensive therapy. *High Blood Pressure and Cardiovascular Prevention Conference: 2013 National Congress of the Italian Society of Hypertension, SIIA 2013 Rome Italy Conference Start: 20131003 Conference End: 20131005 Conference Publication: (var pagings)* 2013;20(3):September. doi: http://dx.doi.org/10.1007/s40292-013-0021-4
56. Rozenfeld S, Bastos Camacho LA, Peixoto VR. Medication as a risk factor for falls in older women in Brazil. *Revista Panamericana de Salud Publica/Pan American Journal of Public Health* 2003;13(6):01.
57. Soysal P, Yay A, Isik AT. Does 25-hydroxyvitamin D deficiency increase orthostatic hypotension risk in the elderly patients? *European Geriatric Medicine Conference: 10th International Congress of the European Union Geriatric Medicine Society - Geriatric Medicine Crossing Borders, EUGMS 2014 Rotterdam Netherlands Conference Start: 20140917 Conference End: 20140919 Conference* 2014;5(pp S120):September.
58. Veronese N, Bolzetta F, De RM, et al. Serum 25-hydroxyvitamin D and orthostatic hypotension in old people: The Pro.V.A. study. *Hypertension* 2014;64(3):September. doi: http://dx.doi.org/10.1161/HYPERTENSIONAHA.114.03143

For peer review only - http://bmjopen.bmj.com/site/about/guidelines.xhtml

59. O'Connell MDL, Savva GM, Fan CW, et al. Orthostatic hypotension, orthostatic intolerance and frailty: The Irish Longitudinal Study on Aging-TILDA. *Archives of Gerontology and Geriatrics* 2015;60(3):01. doi: http://dx.doi.org/10.1016/j.archger.2015.01.008
60. Rockwood MR, Howlett SE, Rockwood K. Orthostatic hypotension (OH) and mortality in relation to age, blood pressure and frailty. *Archives of Gerontology & Geriatrics* 2012;54(3):e255-e60. doi: http://dx.doi.org/10.1016/j.archger.2011.12.009
61. Lagro J, Laurenssen NCW, Schalk BWM, et al. Diastolic blood pressure drop after standing as a clinical sign for increased mortality in older falls clinic patients. *Journal of Hypertension* 2012;30(6):June. doi: http://dx.doi.org/10.1097/HJH.0b013e328352b9fd
62. Madhavan G, Goddard AA, McLeod KJ. Prevalence and Etiology of Delayed Orthostatic Hypotension in Adult Women. *Archives of Physical Medicine and Rehabilitation* 2008;89(9):September. doi: http://dx.doi.org/10.1016/j.apmr.2008.02.021
63. Weiss A, Beloosesky Y, Grinblat J, et al. Seasonal changes in orthostatic hypotension among elderly admitted patients. *Aging Clinical and Experimental Research* 2006;18(1):February.
64. Pathak A, Lapeyre-Mestre M, Montastruc J-L, et al. Heat-related morbidity in patients with orthostatic hypotension and primary autonomic failure. *Movement Disorders* 2005;20(9):September. doi: http://dx.doi.org/10.1002/mds.20571
65. Ooi WL, Barrett S, Hossain M, et al. Patterns of orthostatic blood pressure change and their clinical correlates in a frail, elderly population. *Journal of the American Medical Association* 1997;277(16):23.
66. Ward C, Kenny RA. Reproducibility of orthostatic hypotension in symptomatic elderly. *American Journal of Medicine* 1996;100(4):April. doi: http://dx.doi.org/10.1016/S0002-9343%2897%2989517-4
67. Weiss A, Grossman E, Beloosesky Y, et al. Orthostatic hypotension in acute geriatric ward: Is it a consistent finding? *Archives of Internal Medicine* 2002;162(20):15. doi: http://dx.doi.org/10.1001/archinte.162.20.2369
68. Youde JH, Manktelow B, Ward-Close S, et al. Measuring postural changes in blood pressure in the healthy elderly. *Blood Pressure Monitoring* 1999;4(1):1999.

For peer review only

For peer review only - http://bmjopen.bmj.com/site/about/guidelines.xhtml

Medline and Embase Search Strategy

Date of search 20th October 2015

	**Searches**	**Results**
1	postural hypotension.mp. [mp=ti, ab, hw, tn, ot, dm, mf, dv, kw, nm, kf, px, rx, an, ui]	3109
2	orthostatic hypotension.mp. [mp=ti, ab, hw, tn, ot, dm, mf, dv, kw, nm, kf, px, rx, an, ui]	22694
3	1 or 2	21615
4	prevalence.mp. [mp=ti, ab, hw, tn, ot, dm, mf, dv, kw, nm, kf, px, rx, an, ui]	1202953
5	3 and 4	1678
6	limit 5 to humans	1565
7	limit 6 to aged <65+ years> [Limit not valid in Ovid MEDLINE(R),Ovid MEDLINE(R) In-Process; records were retained]	661
8	remove duplicates from 7	470

For peer review only

TRIPOD Checklist: Prediction Model Development and Validation

Section/Topic	Item		Checklist Item	Page
**Title and abstract**				
Title	1	D;V	Identify the study as developing and/or validating a multivariable prediction model, the target population, and the outcome to be predicted.	1
Abstract	2	D;V	Provide a summary of objectives, study design, setting, participants, sample size, predictors, outcome, statistical analysis, results, and conclusions.	3
**Introduction**				
Background and objectives	3a	D;V	Explain the medical context (including whether diagnostic or prognostic) and rationale for developing or validating the multivariable prediction model, including references to existing models.	4
	3b	D;V	Specify the objectives, including whether the study describes the development or validation of the model or both.	4
**Methods**				
Source of data	4a	D;V	Describe the study design or source of data (e.g., randomized trial, cohort, or registry data), separately for the development and validation data sets, if applicable.	4
	4b	D;V	Specify the key study dates, including start of accrual; end of accrual; and, if applicable, end of follow-up.	4
Participants	5a	D;V	Specify key elements of the study setting (e.g., primary care, secondary care, general population) including number and location of centres.	4
	5b	D;V	Describe eligibility criteria for participants.	4
	5c	D;V	Give details of treatments received, if relevant.	N/A
Outcome	6a	D;V	Clearly define the outcome that is predicted by the prediction model, including how and when assessed.	5
	6b	D;V	Report any actions to blind assessment of the outcome to be predicted.	5
Predictors	7a	D;V	Clearly define all predictors used in developing or validating the multivariable prediction model, including how and when they were measured.	5 & table 1
	7b	D;V	Report any actions to blind assessment of predictors for the outcome and other predictors.	5
Sample size	8	D;V	Explain how the study size was arrived at.	6
Missing data	9	D;V	Describe how missing data were handled (e.g., complete-case analysis, single imputation, multiple imputation) with details of any imputation method.	N/A
Statistical analysis methods	10a	D	Describe how predictors were handled in the analyses.	5
	10b	D	Specify type of model, all model-building procedures (including any predictor selection), and method for internal validation.	5
	10c	V	For validation, describe how the predictions were calculated.	5
	10d	D;V	Specify all measures used to assess model performance and, if relevant, to compare multiple models.	5
	10e	V	Describe any model updating (e.g., recalibration) arising from the validation, if done.	N/A
Risk groups	11	D;V	Provide details on how risk groups were created, if done.	N/A
Development vs. validation	12	V	For validation, identify any differences from the development data in setting, eligibility criteria, outcome, and predictors.	None table 2
**Results**				
Participants	13a	D;V	Describe the flow of participants through the study, including the number of participants with and without the outcome and, if applicable, a summary of the follow-up time. A diagram may be helpful.	5
	13b	D;V	Describe the characteristics of the participants (basic demographics, clinical features, available predictors), including the number of participants with missing data for predictors and outcome.	table 2
	13c	V	For validation, show a comparison with the development data of the distribution of important variables (demographics, predictors and outcome).	table 2
Model development	14a	D	Specify the number of participants and outcome events in each analysis.	5
	14b	D	If done, report the unadjusted association between each candidate predictor and outcome.	table 3
Model specification	15a	D	Present the full prediction model to allow predictions for individuals (i.e., all regression coefficients, and model intercept or baseline survival at a given time point).	table 4
	15b	D	Explain how to the use the prediction model.	6
Model performance	16	D;V	Report performance measures (with CIs) for the prediction model.	6 table 5
Model-updating	17	V	If done, report the results from any model updating (i.e., model specification, model performance).	6
**Discussion**				
Limitations	18	D;V	Discuss any limitations of the study (such as nonrepresentative sample, few events per predictor, missing data).	6-7
Interpretation	19a	V	For validation, discuss the results with reference to performance in the development data, and any other validation data.	6-7
	19b	D;V	Give an overall interpretation of the results, considering objectives, limitations, results from similar studies, and other relevant evidence.	6-7
Implications	20	D;V	Discuss the potential clinical use of the model and implications for future research.	8
**Other information**				
Supplementary information	21	D;V	Provide information about the availability of supplementary resources, such as study protocol, Web calculator, and data sets.	N/A
Funding	22	D;V	Give the source of funding and the role of the funders for the present study.	14

For peer review only

TRIPOD Checklist: Prediction Model Development and Validation

*Items relevant only to the development of a prediction model are denoted by D, items relating solely to a validation of a prediction model are denoted by V, and items relating to both are denoted D;V. We recommend using the TRIPOD Checklist in conjunction with the TRIPOD Explanation and Elaboration document.

For peer review only
